# Unfolding the Determinants of COVID-19 Vaccine Acceptance in China

**DOI:** 10.2196/26089

**Published:** 2021-01-15

**Authors:** Fulian Yin, Zhaoliang Wu, Xinyu Xia, Meiqi Ji, Yanyan Wang, Zhiwen Hu

**Affiliations:** 1 Communication University of China Beijing China; 2 Zhejiang Gongshang University Hangzhou China

**Keywords:** COVID-19 vaccines, COVID-19 vaccination, affordability, efficacy, risk communication, evidence communication, social media, COVID-19, vaccine, communication, risk, acceptance, China, opinion, strategy, promotion

## Abstract

**Background:**

China is at the forefront of global efforts to develop COVID-19 vaccines and has five fast-tracked candidates at the final-stage, large-scale human clinical trials testing phase. Vaccine-promoting policymaking for public engagement is a prerequisite for social mobilization. However, making an informed and judicious choice is a dilemma for the Chinese government in the vaccine promotion context.

**Objective:**

In this study, public opinions in China were analyzed via dialogues on Chinese social media, based on which Chinese netizens’ views on COVID-19 vaccines and vaccination were investigated. We also aimed to develop strategies for promoting vaccination programs in China based on an in-depth understanding of the challenges in risk communication and social mobilization.

**Methods:**

We proposed a novel behavioral dynamics model, SRS/I (susceptible-reading-susceptible/immune), to analyze opinion transmission paradigms on Chinese social media. Coupled with a meta-analysis and natural language processing techniques, the emotion polarity of individual opinions was examined in their given context.

**Results:**

We collected more than 1.75 million Weibo messages about COVID-19 vaccines from January to October 2020. According to the public opinion reproduction ratio (*R_0_*), the dynamic propagation of those messages can be classified into three periods: the ferment period (*R_01_*=1.1360), the revolution period (*R_02_*=2.8278), and the transmission period (*R_03_*=3.0729). Topics on COVID-19 vaccine acceptance in China include price and side effects. From September to October, Weibo users claimed that the vaccine was overpriced, making up 18.3% (n=899) of messages; 38.1% (n=81,909) of relevant topics on Weibo received likes. On the contrary, the number of messages that considered the vaccine to be reasonably priced was twice as high but received fewer likes, accounting for 25.0% (n=53,693). In addition, we obtained 441 (47.7%) positive and 295 (31.9%) negative Weibo messages about side effects. Interestingly, inactivated vaccines instigated more heated discussions than any other vaccine type. The discussions, forwards, comments, and likes associated with topics related to inactivated vaccines accounted for 53% (n=588), 42% (n=3072), 56% (n=3671), and 49% (n=17,940), respectively, of the total activity associated with the five types of vaccines in China.

**Conclusions:**

Most Chinese netizens believe that the vaccine is less expensive than previously thought, while some claim they cannot afford it for their entire family. The findings demonstrate that Chinese individuals are inclined to be positive about side effects over time and are proud of China’s involvement with vaccine development. Nevertheless, they have a collective misunderstanding about inactivated vaccines, insisting that inactivated vaccines are safer than other vaccines. Reflecting on netizens’ collective responses, the unfolding determinants of COVID-19 vaccine acceptance provide illuminating benchmarks for vaccine-promoting policies.

## Introduction

### Background

Vaccines have been proven to be an extremely effective means of dealing with epidemics in the past [1]. However, over the past decades, the antivaccine or antivaccination movement has taken root in Europe and the United States [2,3]. The antivaccine movement, which encourages vaccine hesitancy, has emerged as a significant public health problem, topping the list of threats to global health [4]. For example, it fueled the contagious measles outbreak of 2019 [5,6]. In addition, antivaccination misinformation spreads more quickly than positive counterparts [7]. Immediately after declaring COVID-19 as a pandemic, numerous conspiracy theories were shared through social media [8-10]. In Pakistan, for example, two well-known political figures expressed anti–COVID-19 vaccine sentiments to the local community and further encouraged vaccine hesitancy [11]. Neil et al [12] proposed a heuristic map of COVID-19 vaccines’ online contentions, which revealed a multisided landscape of unprecedented intricacies about vaccines. The reasons for vaccine refusal are complex and vary by geographical and sociocultural contexts.

Many studies have shown that even vaccinated individuals may have substantial doubts and concerns regarding vaccination [13-15]. Many experts believe that vaccination programs are threatened by growing concerns in the population regarding vaccines’ safety and efficacy [16-18]. According to previous estimates, less than 5%-10% of individuals have strong antivaccination convictions [19]. However, a more significant proportion could be categorized as being vaccine hesitant [20]. Vaccine hesitancy, defined as individual-level reluctance to receive vaccines, may be fueled by a spectrum of held views regarding vaccination spanning from cautious acceptors to outright deniers [21-23]. Amin et al [24] proposed that values-based messages appeal to core morality, influencing individuals’ attitudes on topics like vaccination. They showed via two correlational studies that harm and fairness are not significantly associated with vaccine hesitancy, but purity and liberty are. In addition, politics and public trust may affect public perceptions of vaccine risks. Larson [25] discusses the following aspects: some risks of vaccines, such as side effects, provoke anxiety, reluctance, and rejection of vaccination; when vaccines are regulated, and sometimes mandated, by the government, it is resisted by those who feel their freedom is being imposed upon. Those who do not trust the government sometimes extend their distrust to vaccines produced by pharmaceutical companies, which will generate profits and incite public concern about the motives of vaccine production. By examining the antivaccine situation in Texas, United States, Martin [26] also concluded that the antivaccine community, at large, believes that vaccines are a tool used for government control that makes big pharmaceutical companies wealthy and have side effects that can cause lasting damage. Among the barriers to universal vaccination, misinformation regarding the benefits, medicinal composition, and adverse effects of vaccination limits individuals’ understanding and overall buy-in [27]. Vaccine safety concerns continue to be an essential driver of decreasing vaccine uptake in most contexts [28-30]. Several reports indicate that people’s opinions of vaccination have a significant influence on a vaccine’s development and marketing.

COVID-19 has been demonstrated to have high human-to-human transmissibility [31-33]. The ability of SARS-CoV-2 to infect people through asymptomatic carriers is difficult to detect, making the disease a confounding public health challenge [34-36]. Therefore, vaccine development studies have been carried out by the research teams of various companies and universities worldwide [37,38]. Among them, Chinese research on COVID-19 vaccines is a special case, which covers almost all types of vaccines. However, COVID-19 vaccine development has incited heated discussions [39]. Different countries have varying attitudes toward vaccine development. For example, the Japanese government is considering free COVID-19 vaccination for all residents when it becomes available [26]. In California, United States, some individuals carried placards with antivaccine slogans at rallies to protest against the lockdown. Subsequently, antivaccine movements have also taken place in London, United Kingdom, and other cities [40,41]. In China, although more than 90,000 families have been affected by the epidemic [42], no such movement has taken place. Yet, it does not mean that there is no contention about COVID-19 vaccines and vaccination. Hence, it is of great importance to uncover Chinese people’s collective propensities in social dialogues and to aid authorities in making reasonable and informed decisions.

There will be considerable variation by country in terms of COVID-19 vaccine acceptance [43]. To this end, this country-specific study aims to explore the paradigm of public engagement about COVID-19 vaccination to develop practical strategies of preparedness in order to mitigate the pandemic in China. This study investigates the trending topics related to COVID-19 vaccines on Weibo, and obtains public opinions and propensities related to COVID-19 vaccines, such as vaccine price and side effects.

### Study Objectives

We aim to examine what Chinese netizens are concerned about in terms of COVID-19 vaccines and vaccination by profiling pertinent topics on the microblogging platform Weibo. We took random samples of more than 10 million Weibo messages from January to October 2020 to address the following research issues: the affordability of the COVID-19 vaccine candidates; the efficacy of the COVID-19 vaccine candidates; and propensities concerning COVID-19 vaccination. We also aimed to unveil the underlying motives behind these public appeals and explore potential strategies of preparedness for health and risk communication.

## Methods

### Data Collection

Weibo is thought of as a natural experiment that profiles social responses to Chinese public health preparedness. As the leading Chinese social media characterized by heterogeneous communities, it is a crucial public opinion platform in China. As of December 2018, Weibo had 462 million active users per month, which has increased by more than 70 million for 3 consecutive years, and had 200 million active users with 130 million words posted per day [44]. Chinese netizens regard this platform as a preferred outlet for expressing their demands and appeals [45]. More and more messages were posted, read, forwarded, and commented on than any other platform. Clusters of messages can be found on different topics marked by the hashtag symbol (#), which groups similar content. By organizing the same information into a topic, users can quickly find what they want to understand or express, thus resulting in large-scale participation. This mechanism is also a routine way of compiling comprehensive reflections of peoples’ opinions.

In this study, we retrieved more than 1.75 million Weibo messages with approximately 21.17 billion links posted worldwide from January to October 2020. In addition, we classified the reliability of the messages being circulated. The messages were in 108 languages from around the world, but because of our data filtering and enrichment procedures, the largest fraction of analyzed messages point to Chinese-language sources (Simplified Chinese and Traditional Chinese). Additionally, for each message, verification was performed by Sina Corporation to clearly identify accounts of public interest and certify their authenticity, according to China’s real-name verification policy for the use of microblogs. The findings reported in this study mainly captured the social behaviors of the Chinese-speaking portion of Weibo users, including domestic Chinese and those living abroad.

We utilized natural language processing to screen all Weibo topics about COVID-19 vaccines from the end of January to the beginning of October and obtained 989 topics. Of those, the typical, pertinent, and clustered topics are highlighted in Table 1.

**Table 1:** Topics related to the COVID-19 vaccine, with metadata on topic name, reading quantity, and date.

Topic name	Readers (million), n	Date
#COVID-19 vaccine could be available in early 2021#	820	February 9
#Wei Chen’s Team conducts the Phase I clinical trial of COVID-19 vaccine#	243.8	March 20
#When will the COVID-19 vaccine be available#	50.9	April 14
#China’s COVID-19 vaccine has entered Phase II clinical trial#	33	April 14
#The first participant of the COVID-19 vaccine has not yet shown adverse reactions#	21	April 14
#The world’s first COVID-19 inactivated vaccine#	41.2	April 19
#What is the COVID-19 inactivated vaccine#	25.2	April 21
#Chinese first COVID-19 inactivated vaccine entered Phase II clinical trial#	4566.4	April 24
#Phase I clinical trial of the first Chinese COVID-19 vaccine has good results#	934.5	May 22
#The safety and effectiveness of the COVID-19 inactivated vaccine have been verified#	76.9	May 29
#More than 2000 people received the COVID-19 inactivated vaccine injection#	479.6	May 30
#COVID-19 inactivated vaccine is expected to be available at the end of this year or early next year#	162.1	May 30
#China developed another kind of COVID-19 inactivated vaccine#	2000	June 9
#World’s first COVID-19 inactivated vaccine participant produces antibodies#	39.4	June 17
#Chinese COVID-19 vaccine will be launched as early as 2021#	37.9	June 18
#CNBG’s COVID-19 inactivated vaccine is not affected by virus mutation#	1276.2	June 19
#Chinese three COVID-19 vaccines complete Phase II clinical trials#	18,000	June 20
#Domestic COVID-19 inactivated vaccine launches international clinical phase Ⅲ trial#	84.2	June 23
#COVID-19 inactivated vaccine production workshop is completed in Wuhan#	7388.4	July 2
#WHO requires the protection period of the COVID-19 vaccine to be at least six months#	56.8	July 3
#Chinese COVID-19 vaccine Phase 2 clinical trial achieves good results#	131	July 21
#COVID-19 vaccine may be available at the end of the year#	345.4	July 22
#World first officially releases Phase II clinical data of COVID-19#	238.3	July 23
#The price of the COVID-19 vaccine will not exceed $40#	888.9	July 29
#Russian COVID-19 vaccine will be free of charge#	20,000	August 1
#The first COVID-19 inactivated vaccine workshop passed safety inspection#	44.9	August 5
#Beijing COVID-19 inactivated vaccine production workshop can be put into production at any time#	45.5	August 5
#Half of the COVID-19 vaccines in Phase III clinical trials come from China#	150.6	August 7
#Gates required the COVID-19 vaccine to be priced below $3#	963.7	August 8
#Research Institute refutes rumors of COVID-19 vaccine 498 yuan an injection#	127.4	August 13
#COVID-19 vaccine not yet be available#	3	August 13
#COVID-19 inactivated vaccine two injections less than one thousand yuan#	3081.6	August 18
#Domestic COVID-19 inactivated vaccine is expected to be available at the end of December#	131.6	August 18
#Russian second COVID-19 vaccine starts Phase 2 clinical trial#	70.1	August 18
#COVID-19 vaccine two injections 1000 yuan is too expensive#	24.2	August 19
#The price of COVID-19 vaccine can only be based on cost#	17,000	August 23
#How to price the COVID-19 vaccine#	254.2	August 23
#CNBG declared COVID-19 vaccine is likely to be available at the end of this year#	354.6	August 23
#National Health Commission claimed that the price of COVID-19 vaccine was lower than two injections of 1,000 yuan#	65.9	August 23
#Domestic COVID-19 inactivated vaccine first appears#	19,000	September 5
#Domestic COVID-19 inactivated vaccine appears in CIFTIS#	5766.4	September 6
#The COVID-19 vaccine will be priced based on factors such as consumers’ ability to pay#	134.5	September 7
#Domestic COVID-19 inactivated vaccine inoculates hundreds of thousands of people with zero infection#	2102.4	September 11
#Oxford vaccine volunteers have side effects#	769.1	September 9
#The COVID-19 vaccine produced by Pfizer in America has side effects#	17,000	September 16
#The COVID-19 inactivated vaccine is expected to be available at the end of the year#	4319.2	September 8
#The COVID-19 vaccine price in China will be within the scope of public acceptance#	11,000	September 2
#The COVID-19 inactivated vaccine is only one kilometer away from success#	23,000	September 2
#Two injections 600 yuan for domestic COVID-19 vaccine #	7737	September 3
#The price of domestic COVID-19 vaccine is released#	4413.4	September 2
#Four COVID-19 vaccines in China enter Phase III clinical trials#	428.6	September 2
#China formally joins COVAX#	23,000	October 9

### Behavioral Dynamics Model

**Figure 1:**
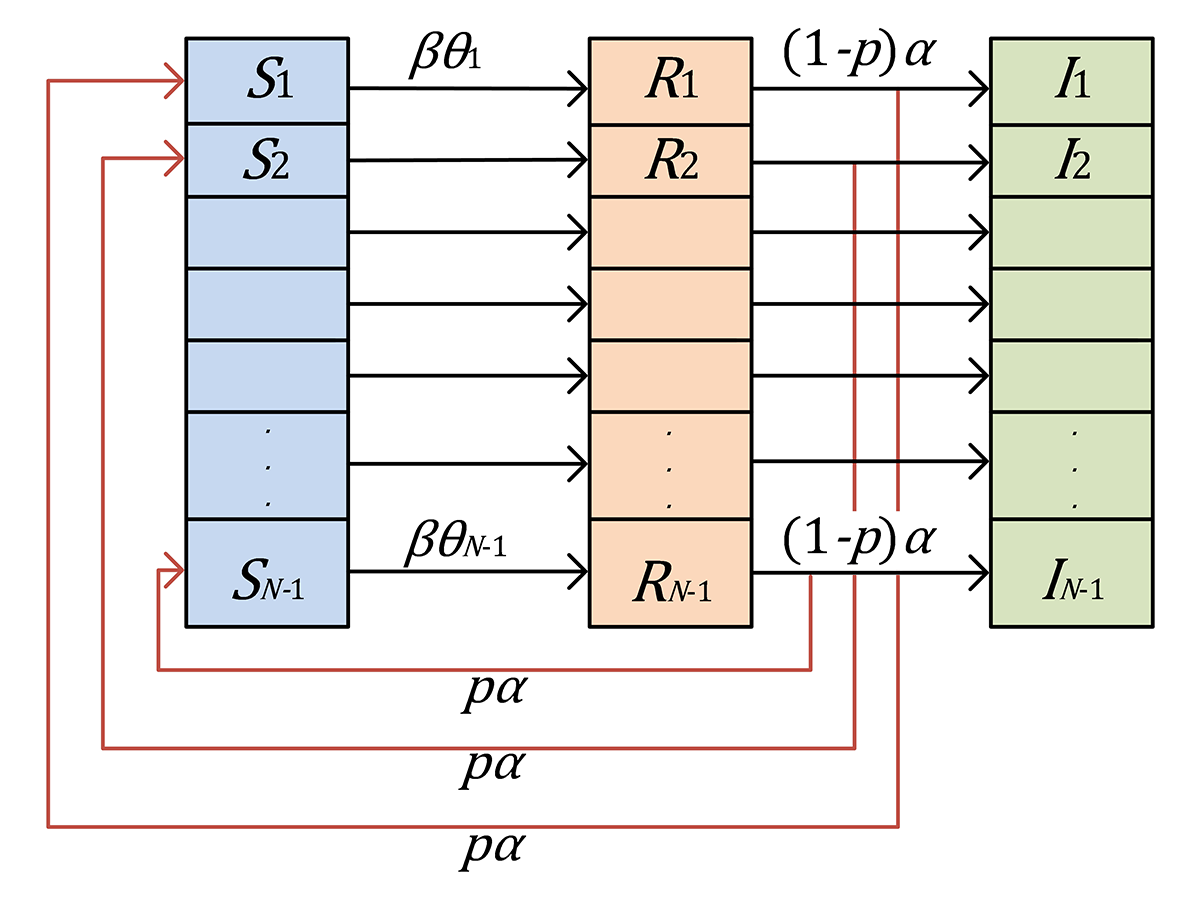
Inspired by infectious disease models, a network model diagram of information dissemination is proposed to simulate the transmission of information among the susceptible state (S), the reading state (R), and the immunized state (I).

On social media, the propagation and inline influence of various topics are involved. To track the derivative development of topics related to COVID-19 vaccines in China, we propose the dynamics model SRS/I (susceptible-reading-susceptible / immune) based on a complex network to investigate the landscape of public opinion transmission (Figure 1) [46]. The SRS/I model promises to profile the collective propensities of different populations in terms of different topics across various times on social media.

We considered a reading population of Weibo users for topics about the vaccine, stratified in terms of three distinct stages: the susceptible state (S), in which users are unaware of but susceptible to the information about the event; the reading state (R), in which users have actively read information to influence other users; and the immunized state (I), in which users have read the information and can trigger a new round of reading activities on the same topic. We obtain the following SRS/I reading dynamics model of vaccines topics:

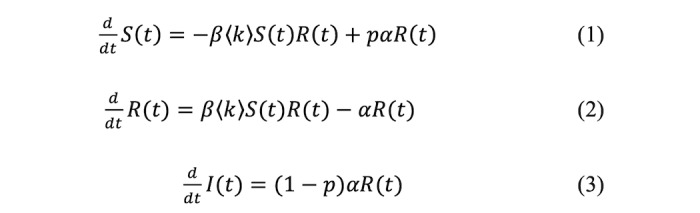



where *β* refers to the average exposure rate of a susceptible user who can read topics about the vaccine. Since an active reading user will contact an average number of *βN* users per unit time and the probability of a contacted user being a susceptible user is *S(t)/N*, the number of new reading users is *β*〈*k*〉*N*(*S(t)/NR(t) = β*〈*k*〉*S(t)R(t)*. Users can become inactive to the same topic with an average inactive rate *α,* with 1/*α* being the average duration where an *R*-user remains active in reading. The average number of inactive users will be *αR*(*t*) per unit time, among which *pαR*(*t*) will re-enter the susceptible state where exposure to another Weibo message within the same topic can initiate a new round of reading, and (*1*–*p*)*αR*(*t*) will proceed to the immunized state directly, in which *p* reflect the re-entering probability for a reading user who can trigger a new round of reading activities on the same topic. *θ* is a parameter related to the topics’ dissemination network of topics pertaining to vaccines. In this paper, we only considered the average degree 〈*k*〉.

We extended the basic *R_0_* [47] of epidemiology to the field of information transmission. In our SRS/I dynamics model, we defined the public opinion *R_0_* as a measure of the potential impacts of topics in the initial propagation stage, which is given by *R'*(0) *=* (*β*〈*k*〉*S*_0_ – *α*)*R*(*0*), and the outbreak will never grow since *R'*(0) *=* (*β*〈*k*〉*S*_0_ – *α*)*R*(*0*) < 0 due to the decrease of *S*. Then we deduce:

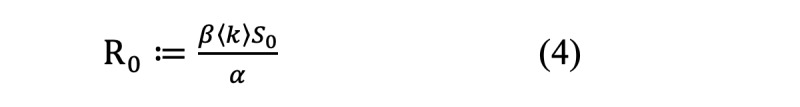



To further explore Chinese opinion about COVID-19 vaccines, we collected metadata, which includes the names and reading quantity of related topics from the end of January to the beginning of October, through an application programming interface provided by Weibo. Table 1 shows several specific topics related to COVID-19 vaccines with their posted date and reading quantity. The date can aid in identifying the continuity of topics over time, while the reading quantity could reflect the collective interest of the population.

### The Landscape of Public Opinion Transmission

**Figure 2:**
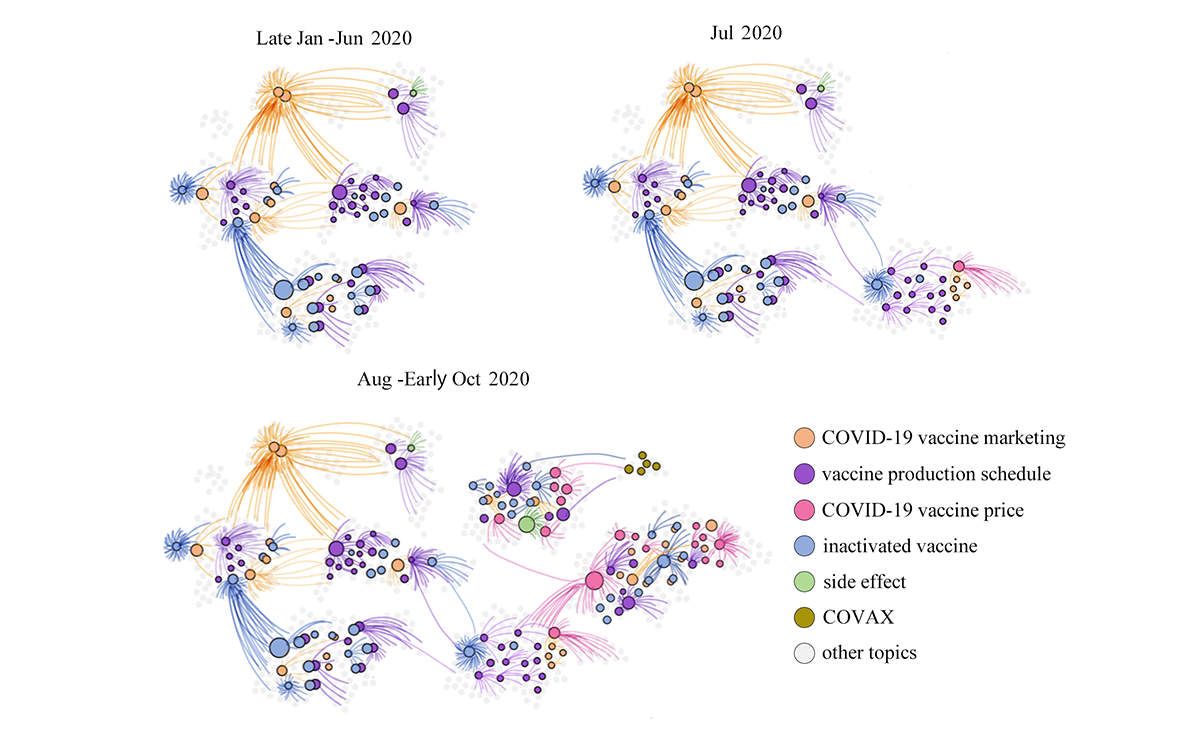
The landscape of public opinion transmission on vaccine-related topics. The colored dots represent the seven main topics. The lines between them represent mutual influence, and the density of the lines represents the degree of influence.

After numerical fitting and calculation, according to the basic *R_0_*, we found that a paradigm shift emerged in July 2020. The *R_0_* value becomes large at this time, implying that Chinese netizens had become more interested in vaccines. Therefore, we divided the transmission process of vaccine-related topics into three periods: ferment period (stage 1, the end of January to June), evolution period (stage 2, July), and transmission period (stage 3, August to the beginning of October) (Figure 2). Their public opinion reproduction ratio is specifically expressed as *R_01_*, *R_02_*, and *R_03_*, where *R_01_*=1.1360, *R_02_*=2.8278, and *R_03_*=3.0729. It is evident that *R_03_* is larger than *R_01_* and *R_02_* This is consistent with the fact that the topics in stage 3 have been disseminated more widely than those in stages 1 and 2. In stage 3, two sensitive topics came to our attention: vaccines’ price and vaccines’ side effects. Topics about vaccines’ side effects (labels 44 and 45 in the Multimedia Appendix 1) reached more than 17,000,000,000 readers while topics about vaccine pricing (labels 24 and 25 in the Multimedia Appendix 1) have emerged in an endless stream and have been widely read. So, they incite continuous attention from Chinese netizens.

As shown in Figure 2, we obtained the metadata of 989 topics on Weibo. We considered the duration and content of topics that Chinese people are interested in. Topics were divided into seven categories based on content. We found topics connected with the same specific event are inline as time goes by. Namely, one topic, whether it has a strong positive or negative sentiment or is just a general announcement, may affect the sensitivity of Chinese netizens to the COVID-19 vaccine, thereby involving the population to facilitate the creation of new topics or a derivation of old ones.

### Price Acceptance

We collected all messages and their likes on all price-related topics mentioned above. We set keywords and used the following formula to calculate the price tendency. After tokenization and the word extraction process, we obtain *M* words, which are synonyms for “expensive,” expressed as (*E*_1_, *E*_2_,…,*E_M_*). By querying in the dictionary (Multimedia Appendix 2), we denoted the weight of the *i_th_* word as *a*_i_. Hence, the score of “expensive” is computed as:

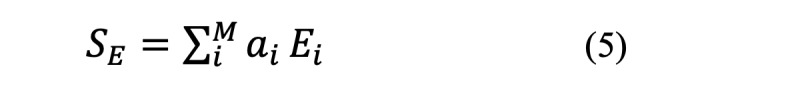



Similarly, we obtained *N* words, synonyms for “cheap,” expressed as (*C_1_*, *C_2_*,*…*,*C_N_*). After querying in the dictionary, the weight of the *i_th_* word *a*_i_ was obtained. The score of “cheap” is expressed as:

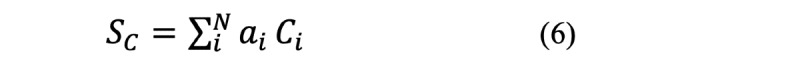



The final expression of the price tendency score can be calculated as:

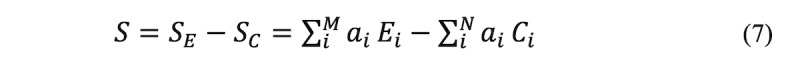



When *S*>0, the population regards the price as high, and when *S*<0, the collective attitudes toward price are acceptable.

### Sentiment Polarity of Side Effects

We analyze the sentiment of 925 messages on topics related to side effects based on the general Chinese lexicon HowNet [48]. For the text sequence *x*={*w_1_*,… …,*w_k_*,… …,*w_K_*}, *w_k_* indicates that the *k*-th word in sequence *x*, and *K* is the total word number for the sequence *x*. Then we obtained the corresponding sentimental values *Sen_x_*={*S_U_*(*w_1_), … …, S_U_*(*w_k_*), … …, *S_U_*(*w_K_*)} of each word in *x* by sentiment lexicon rescores to synthesize the final sentence sentiment value *S_x_*:

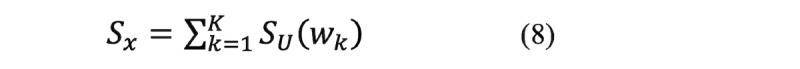



where *S_U_*(*w_k_*) is the sentiment value for the *k*-th word in sequence *x* calculated by the sentiment lexicon we used, and *S_x_* is the sentiment value of the sequence *x*. We turned *S_x_* into polarity *T_x_* as follows to easily judge the performance of our sentiment classification task:

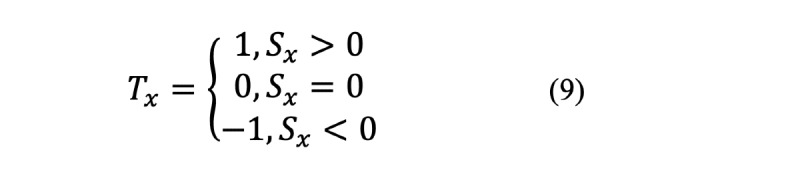



where we count the sum of *T_x_* of each case, denoted by *NP*, *NN*, and *ZN*, respectively. We further calculated the proportion of each sentiment tendency in all corpora:

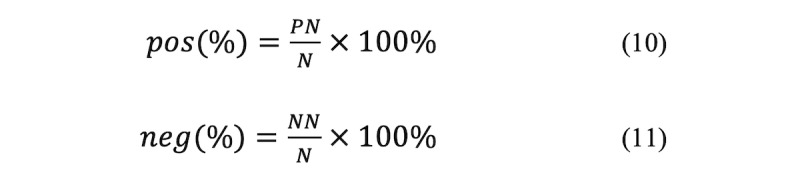



where *N* is the total number of corpora, *pos*(%) and *neg*(%) are represented as positive and negative sentiment proportions, respectively.

In the sampling inspection, due to the Chinese language’s diversity, we validated the results of the sentiment lexicon–based method. We strictly followed the requirements of the double-blind experiment and invited three groups (A, B, and C) who had been trained to classify these Weibo messages by emotion. When the emotion judged by groups A and B was consistent, we took it as the correct result. When the judgment between groups A and B was inconsistent, group C made the final judgment. We marked positive Weibo messages as 1, and negative ones as –1, using the equations 10 and 11 to calculate manual labeling.

## Results

### Weibo’s Attitude Influence Map

**Figure 3:**
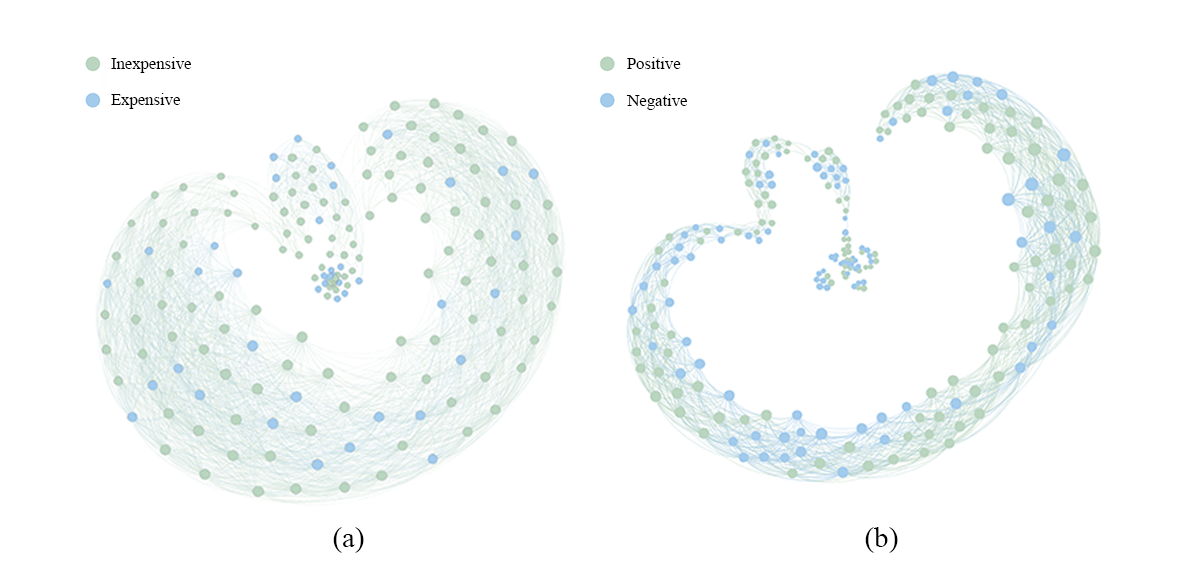
Weibo's attitude contagion map: (A) price and (B) side effects. Points represent Weibo messages that appear in the figure in clockwise order, ending with the point at the center of the figure.

In the case of affordability and efficacy, we chose two topics—#The COVID-19 vaccine price can only be based on cost# (n=169 messages) and #The COVID-19 vaccine produced by Pfizer in America has side effects# (n=220 messages). We utilized the SRS/I model to draw the messages’ attitude influence map (Figure 3). The colors represent the different attitudes. A topic is composed of individual Weibo messages, which have a content connection and time-sequence relationship. At first, the first Weibo message had the ability to promote subsequent publishing and dissemination. In Figure 3, we used points to represent Weibo messages, which appear clockwise in chronological order. In terms of price (Figure 3A), most people thought the pricing of vaccines was inexpensive. Nevertheless, it is worth noting that many still feel the pricing to be expensive, as seen by Weibo messages posted at various time periods. In terms of side effects (Figure 3B), positive Weibo messages dominated initially, but over time, positive and negative views alternated in being the majority, and the two views clashed during this time. Overall, positive dominates.

### Affordability: The Price of Vaccines

**Figure 4:**
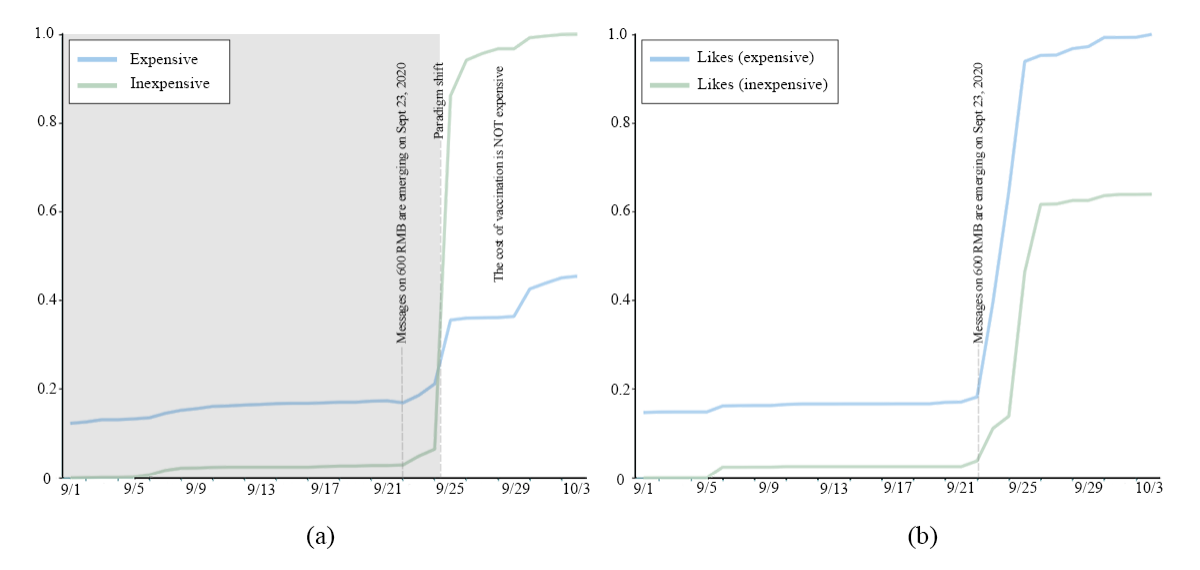
The paradigm shift in price tendency.

We selected metadata during the period when the paradigm of price tendency dramatically changed and obtained 4925 related messages on Weibo. The final result is normalized (Figure 4). Figure 4A shows that the collective attitudes toward COVID-19 vaccine pricing suddenly changed after September 23 from expensive to inexpensive. However, the population’s likes displayed the opposite tendency, as shown in Figure 4B.

On Weibo, the topic of COVID-19 vaccine pricing emerged in July 2020 and attracted hundreds of millions of onlookers in China. However, the number of messages that specifically address pricing is not very large.

In August, the Chinese government claimed that the Chinese vaccine price would not exceed 1000 RMB (US $154). Therefore, the expected price was about 1000 RMB, and most people thought it was too expensive. Due to the one-child policy, young people may need to pay for their extended family. Although they believe that the cost of production is high, the cost of vaccination is not a small expenditure for ordinary families. After the Chinese COVID-19 vaccine fee was announced on September 23—600 RMB (US $90) for 2 shots—the number of relevant messages began to increase significantly (label 49 in Multimedia Appendix 1). As shown in Figure 4A, it is worth noting that the public had their own speculations about the prices of Chinese vaccines before this announcement. The public’s attitudes reversed after this tipping point, and most people found the official price to be acceptable. Most people clicked “like” or posted Weibo messages to endorse previous views on Weibo rather than posting similar messages themselves. As shown in Figure 4B, the collective emotion polarity did not reverse with the increase in likes.

Certainly, the official price is still too expensive for some netizens. Comparatively speaking, citizens in developed countries have their own universal health insurance, or some countries promise to bear the costs of vaccinations. As a case in point, the Japanese and Russian governments have promised to pay to vaccinate its citizens (labels 25 and 52 in the Multimedia Appendix 1, respectively). Therefore, some Chinese netizens naturally hope that China will follow suit. The patterns in Figure 3A also confirm the above findings.

### Efficacy: Side Effects of Vaccines

**Figure 5:**
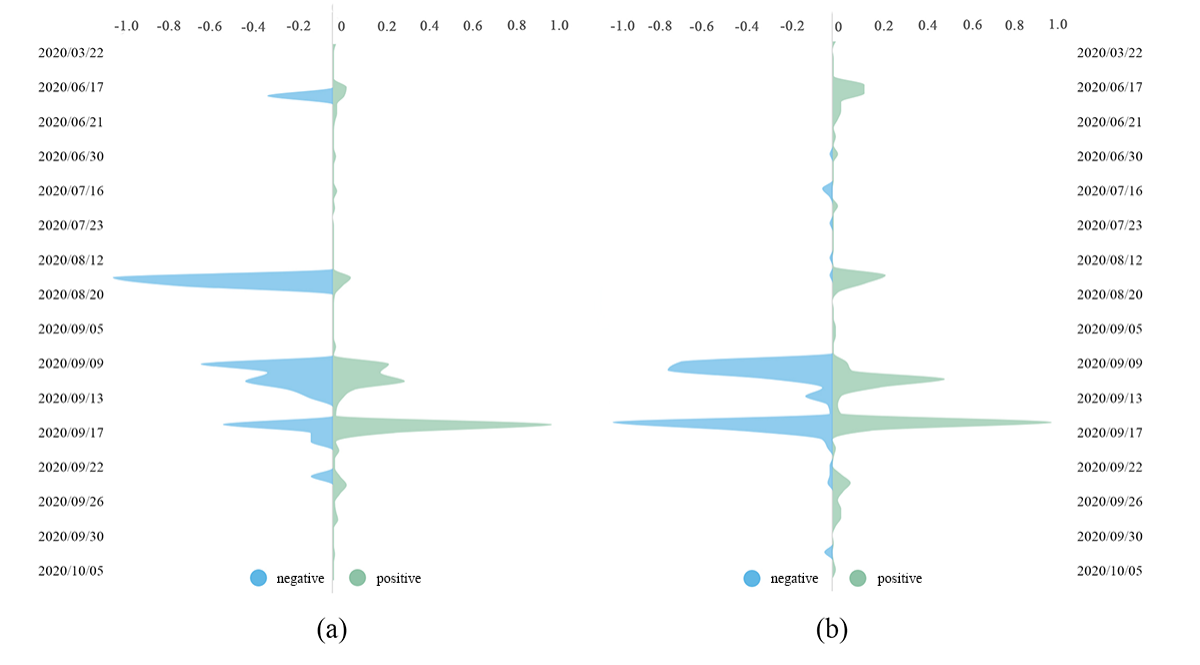
The collective attitudes toward side effects: (A) the results of the lexicon-based method and (B) the final results coupled with manual labeling.

We plotted the lexicon-based sentiment classification results and the manually labeled sentiment classification method in Figure 5. Generally speaking, Chinese people are optimistic about vaccines. Interestingly, Chinese netizens seem to be accustomed to expressing their positive opinions in diverse ways. These utterances may undermine the accuracy rate of lexicon-based sentiment analysis, especially for negative sentiments (Figure 5). Potential uncertainties may cause a miscalculation of the accuracy rate of emotion-cause pair extraction and identification of ironic contexts. For example, comparing Figure 5A with Figure 5B, Chinese netizens tend to express their understanding of vaccine side effects using irony. In one Weibo post, for instance, a user wrote, “I think most people cannot even understand the title,” while another wrote, “if you have a little common sense in pharmacology… Is it weird to have an adverse event (AE)? How can it be on the hot search?” These Weibo messages’ authors believe that it is common sense for drugs to have side effects. They have a positive attitude toward side effects but scoff at those who have opposing opinions. However, these Weibo messages were judged neutral by the machine. There were also some Weibo messages judged to be negative, although their attitude toward side effects is positive. For example, one message read, “Even taking vitamins will increase the liver’s metabolic burden. All drugs are somewhat toxic. If a drug has no side effects, it must be fake.” They know the potential danger of side effects but think it is within the acceptable range. Thus, their attitude toward vaccines’ side effects is positive. Due to irony, the results of the lexicon-based sentiment classification (Figure 5A) are inaccurate. Therefore, we only used the results obtained by manual labeling to determine Chinese netizens’ attitudes toward side effects.

Figure 5B shows four significant patterns in June, August, and September. The trigger events were #Chinese inactivated vaccine’s Phase I/II trial was complete, and the participants had no adverse reactions# (June 16), #The chairman of Sinopharm said he was vaccinated and no adverse reactions# (August 18), #Oxford vaccine volunteers experienced adverse reactions# (September 9), and #The COVID-19 vaccine produced by Pfizer in America has side effects# (September 16), respectively. The topics were more active (ie, more messages posted) in September. From the perspective of emotion polarity, the messages’ polarity about the side effects of COVID-19 vaccines can directly and positively affect people’s emotion polarity. Over time, positive messages and negative messages became dominant in an alternating manner. In the end, Chinese views on side effects tended to be positive. The possible reason for this finding is that the positive emotions of prevailed messages on side effects result in herd behavior of followers [49]. After such information cascades, the population gradually accepted the existence of side effects and then reached a consensus (Figure 5B). Figure 3B shows similar patterns.

**Figure 6:**
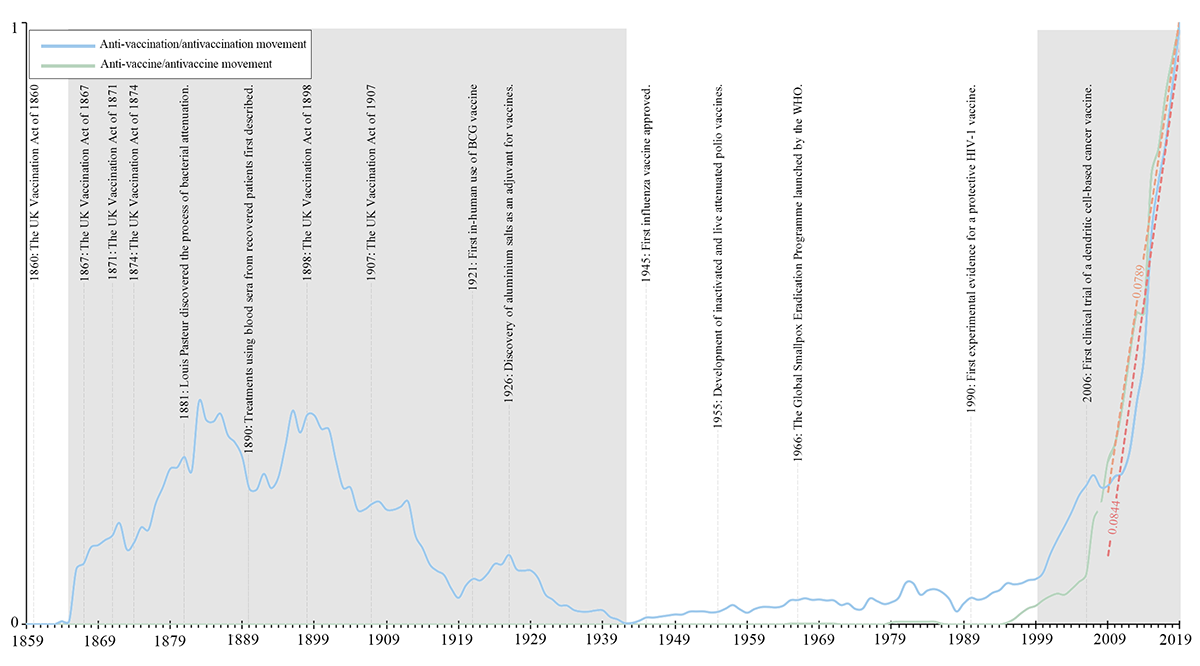
Google Books Ngram Corpus facsimiles of word frequencies for "anti-vaccination/antivaccination movement" and "anti-vaccine/antivaccine movement" in the English corpus from 1859 to 2019. BCG: Bacillus Calmette–Guérin; WHO: World Health Organization.

On the contrary, as *Nature News* reported, in high-income countries such as Europe, citizens’ concerns no longer focus on the price but on safety [49]. Many people believe that vaccines will increase the immune system’s burden, assuming that it exposes themselves to danger. In addition, a variety of other exaggerated rumors have spread on major social media outlets. According to Google Books Ngram Corpus (Figure 6), the co-occurrence of antivaccine and antivaccination movements has shown a clear upward trend in the past decades (the fitted slope of the antivaccination movement is 0.0844 and the antivaccine movement is 0.0789) [50]. The historical events that may have impacted the antivaccine and antivaccination movements in different eras are marked in Figure 6. It also shows that the concern for the safety of early vaccines has resulted in widespread protests. With the development of medical technology, vaccines have gradually become safer, and protests have decreased. Part of the reason for burgeoning movements in recent decades is due to ideological reasons [51]. However, as an exception, China has not experienced such scenarios. In view of this, in China, it is particularly important to collect public opinions via social media and improve policies in a timely manner.

### Hesitancy: Type of Vaccines

Vaccine hesitancy about COVID-19 vaccines is linked to populations who are reluctant or refuse to be vaccinated despite the availability of vaccination services [52]. The beliefs surrounding vaccine hesitancy are dynamic, complex, and context-specific, varying across time, place, and vaccine types, as well as complacency, convenience, and confidence. China is forging ahead in the race to develop COVID-19 vaccine candidates using five potential development routines: inactivated vaccines, adenovirus vector vaccines, vaccines using attenuated influenza viruses as vectors, recombinant protein vaccines, and nucleic acid vaccines (including RNA [ribonucleic acid] vaccines and DNA vaccines) [53]. China’s National Medical Products Administration projected the country’s production capacity of COVID-19 vaccines would reach 610 million doses annually by the end of 2020. Of these, the licensed vaccines for limited rollout would be inactivated vaccines [54].

**Figure 7:**
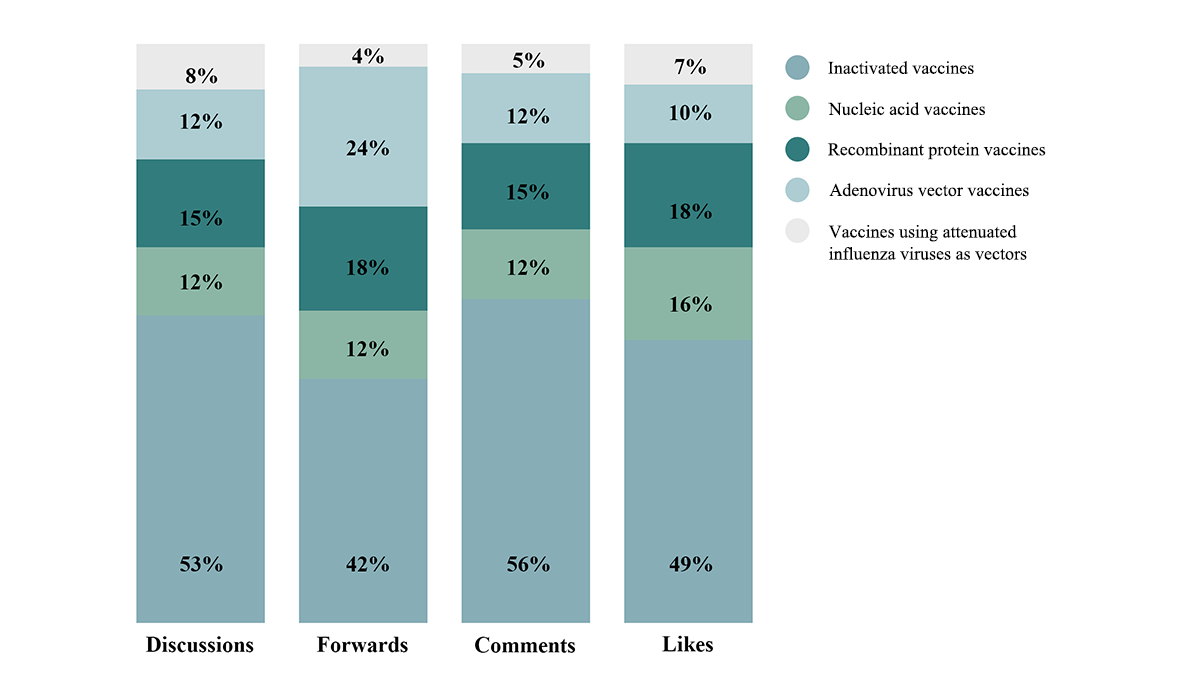
The distribution of netizens’ attention to five different types of China-made vaccines.

Figure 7 shows the proportion of these five vaccines as mentioned by Chinese netizens. We have selected four dimensions—discussions, forwards, comments, and likes—to show the degree of attention on the different vaccines. Evidently, inactivated vaccines had substantially more discussions, forwards, comments, and likes than the other four vaccine types.

Compared with the other vaccines, inactivated vaccines are more acceptable to the public in China. The reason may be that the word “inactivated” in Chinese means “being killed” or “dead”; this gives people a sense of more security. For the same reason, the vaccines using attenuated influenza viruses as vectors, which means “alive influenza virus,” has been rarely mentioned by netizens. It accounts for 8% (87/1117) of mentions (Figure 7 and Multimedia Appendix 3).

## Discussion

### Principal Findings

Using rich random-sampling metadata of Weibo posts (more than 1.75 million messages with approximately 21.17 billion links), we conducted a country-specific study of real-time public awareness and behavioral responses to COVID-19 vaccines and vaccination from January to October 2020 in China. By studying the collective transmission behavior of Chinese netizens (domestic Chinese and those living abroad), this research revealed the paradigm shifts in public demand and events instigating public opinion in the context of the promotion of COVID-19 vaccination.

#### Beyond Affordability

Our findings strongly suggest that the Chinese public is divided in terms of vaccination prices and has differing expectations. Supporters accepted the current official price, whereas opponents claimed that it is unaffordable for their extended family. Arguably, the majority of people think that the price is lower than previously thought. If the Chinese government promises to pay for vaccination, taking 600 RMB per person as an example, the country would bear a financial burden of at least 840 billion RMB, considering its large population size. Therefore, introducing a policy for free COVID-19 vaccination is an extremely difficult decision for the Chinese government to make. If the government simply and directly included COVID-19 vaccines in medical insurance plans, it would impose a financial burden on hospitals, and the costs would eventually pass on to patients [55]. If commercial insurance is subsidized, another problem will arise: Chinese families will tend to insure children, who have a low risk of COVID-19, rather than high-risk elderly people [56,57].

#### Concerns Raised Over Efficacy

Our study is characterized by the striking feature that people’s concerns about side effects tend to be positive over time. News of fast-tracked, China-made vaccine candidates are encouraging and helpful, but scientists urge caution [58]. Admittedly, the volunteers who received either the trial vaccine or placebo were low-risk, healthy adults rather than high-risk populations (eg, obese patients [59,60], patients with autoinflammatory syndrome [61], etc). Many scientists hold that there is uncertainty about the vaccine candidates’ true efficacy when they are extended to the general populations [62]. More observations are needed to test the effectiveness of the vaccine [63,64]. On the contrary, the results of prevailing messages pinpoint that most Chinese people would accept the marginal risks (ie, side effects) with much confidence in the forthcoming vaccines.

#### Cognitive Dissonance Debunked

The literal meaning of the Chinese name for inactivated vaccines may provide a false sense of security. According to our survey, most Chinese people reached a consensus on the safety of inactivated vaccines due to cognitive dissonance. By contrast, people’s expectations for inactivated vaccines were higher than other types of vaccines. There is no scientific evidence supporting the safety of inactivated vaccines compared to the other types. Furthermore, even when the vaccine is proven to be safe and effective, its acceptance will vary by group [65]. The World Health Organization has listed vaccine hesitancy as one of the top 10 threats to global health [66,67]. It is usually caused by the association of moral values in families [24,68], although this is unnecessary [69]. Nevertheless, the biggest problem in China is cognitive dissonance. Being echoed by daily communications, this finding indicates that this false cognitive predisposition may reinforce the tendency to vaccinate using inactivated vaccines and discourage vaccination via other types. Even worse, as vaccines lose their competitive advantages, people may not choose the most suitable one or even lose their right to choose. As rare events often attack the safety of vaccines [70], dissonance can lead to collective misbehavior.

### Limitations

In this study, we retrieved more than 1.75 million Weibo messages in 108 languages; however, only messages in Simplified Chinese and Traditional Chinese were further investigated. These messages may come from domestic Chinese, those living abroad, or even foreign netizens living in China. Therefore, the findings reported in this study reflect COVID-19 vaccine acceptance in the Chinese-speaking population. However, each netizen account is authenticated according to the real-name verification policy of China, and the authenticity of the message can be guaranteed as they almost always come from real people rather than bots. With this in mind, this limitation does not undermine the significance of our findings.

The landscape of public opinion transmission is still ever-changing, especially in terms of price, side effects, etc. As the three major topics are still in the developing stage, a large number of messages and interactions showed up after study completion. However, the paradigm shifts of all the pertinent topics discussed here have been observed. Therefore, this limitation should not undermine the significance of the results either.

In addition, according to the 46th Statistical Report on Internet Development in China released by the China Internet Network Information Center in September 2020 [71] (Multimedia Appendix 4), only 10.3% of Chinese netizens are over 60 years of age. Therefore, to overcome the undersampling of the elderly population in this survey, the determinants of COVID-19 vaccine acceptance among older adults need to be further investigated [43].

### Conclusions

At this critical moment in China, articulating the dynamic social paradigms of public engagement for COVID-19 vaccination is paramount for examining the practical strategies of social mobilization, wherein one sheds light on the other’s significance. We scrutinized collective responses on COVID-19 vaccines and pertinent discourses in sociocultural paradigms to uncover collective propensities and consequences.

As an integral component of preparedness, the contextualized results reported in this study promise to provide illuminating benchmarks to bridge the gaps of health and risk communications. In China, the landscape of public opinion transmission on Chinese social media is unique, featuring a real-name verification policy. Therefore, the online collective propensities on COVID-19 vaccines and vaccination could resonantly echo daily responses in the real world, including those from domestic Chinese and those living abroad. Although there is no need for explicit antivaccine or antivaccination movements, the implicit channel of online public appeals is more vital than ever for improving policies.

The paradigms we identified to be the determinants of COVID-19 vaccine acceptance (eg, public appeals on affordability, efficacy, and preferences) could reframe a heuristic framework for extensive discussions, especially on vaccine-promoting policies in China. Reflecting on the unfolding findings, evidence communication is a heuristic way to nurture trustworthiness [72]. For instance, the government could consider using health insurance to balance state finances with individual expenditure. Making vaccine clinical trial data open and transparent is an effective way to assuage public skepticism. To eliminate strongly held but flawed cognitive predispositions, the government needs to increase the popularization of public science to communicate the side effects of drugs and strengthen publicity for all kinds of vaccines. Moreover, previous studies have indicated that once the vaccines are available, distributing them may become problematic [73-75]. The production capacity of vaccines has also been questioned by scholars. The impending worry is that new rounds of antivaccine and antivaccination movements will sprung up in a backlash of populism [58] and further undermine global efforts to curb the COVID-19 pandemic. Some scholars have turned their attention to disadvantaged groups and believe that the ethical framework needs to be improved to protect these groups’ rights and interests during COVID-19 vaccination [75]. Arguably, affordable and effective vaccines offer hope for ending the pandemic, and open-minded and iterative policies fuel public engagement against the pandemic.

## Appendix

Multimedia Appendix 1 Typical topics related to COVID-19 vaccines, containing information about topic names (with Chinese translation), reading quantity, and date.

Multimedia Appendix 2 Price polarity table.

Multimedia Appendix 3 Discussions, forwards, comments, and likes related to China’s five vaccine types.

Multimedia Appendix 4 Statistical Report on Internet Development in China.

## Abbreviations

AE: adverse event
R_0_: reproduction ratio
RNA: ribonucleic acid
SRS/I: susceptible, reading, susceptible/immunized
