# Supplementary material for: Unfolding the Determinants of COVID-19 Vaccine Acceptance in China
Source: J Med Internet Res. 2021 Jan 15;23(1):e26089. doi: 10.2196/26089 (PMC7813210; doi:10.2196/26089)
Supplement: Multimedia Appendix 1 [file jmir_v23i1e26089_app1.docx]

**Multimedia Appendix 1**

Typical topics related to the COVID-19 vaccines, containing the information about topic name recommendation, reading quantity, and date.

| **Label** | **Topic name recommendation** | **Reading quantity (/million)** | **Date** |
| --- | --- | --- | --- |
| 1 | #新冠肺炎疫苗或在2021年初上市# | 820 | Feb, 9 |
|  | (#COVID-19 vaccines may be listed in early 2021#) |  |  |
| 2 | #陈薇团队新冠疫苗一期临床试验# | 243.8 | Mar, 20 |
|  | (#Wei Chen's Team conducts the Phase I clinical trial of COVID-19 vaccines#) |  |  |
| 3 | #新冠疫苗何时上市# | 50.9 | Apr, 14 |
|  | (#When will the COVID-19 vaccines be available#) |  |  |
| 4 | #我国新冠病毒疫苗进入二期临床试验# | 33 | Apr, 14 |
|  | (#China's COVID-19 vaccines have entered Phase II clinical trial#) |  |  |
| 5 | #首位新冠疫苗受试者尚未收集到不良反应# | 21 | Apr, 14 |
|  | (#The first subject of COVID-19 vaccines has not yet collected adverse reactions#) |  |  |
| 6 | #全球首个新冠灭活疫苗# | 41.2 | Apr, 19 |
|  | (#The world's first COVID-19 inactivated vaccines#) |  |  |
| 7 | #新冠灭活疫苗是个啥# | 25.2 | Apr, 21 |
|  | (#What are the COVID-19 inactivated vaccines#) |  |  |
| 8 | #我国首个新冠灭活疫苗进入二期临床# | 4566.4 | Apr, 24 |
|  | (#China’s first COVID-19 inactivated vaccines entered Phase II clinical trial#) |  |  |
| 9 | #中国首支新冠疫苗一期临床试验结果良好# | 934.5 | May, 22 |
|  | (Phase I clinical trial of the first COVID-19 vaccine in China has good results#) |  |  |
| 10 | #新冠灭活疫苗安全性有效性获验证# | 76.9 | May, 29 |
|  | (#The safety and effectiveness of the COVID-19 inactivated vaccines have been verified#) |  |  |
| 11 | #2000余人接受新冠灭活疫苗注射# | 479.6 | May, 30 |
|  | (#More than 2000 people received the injection of COVID-19 inactivated vaccines#) |  |  |
| 12 | #新冠病毒灭活疫苗预计今年底或明年初上市# | 162.1 | May, 30 |
|  | (#COVID-19 inactivated vaccines are expected to be available at the end of this year or early next year#) |  |  |
| 13 | #中国再添新冠灭活疫苗# | 2000 | Jun, 9 |
|  | (#China developed another kind of COVID-19 inactivated vaccine#) |  |  |
| 14 | #全球首个新冠灭活疫苗受试者产生抗体# | 39.4 | Jun, 17 |
|  | (#Subjects of world's first COVID-19 inactivated vaccine produce antibodies#) |  |  |
| 15 | #中国新冠疫苗最早2021年上市# | 37.9 | Jun, 18 |
|  | (#COVID-19 vaccines in China will be launched as early as 2021#) |  |  |
| 16 | #中国生物新冠灭活疫苗不受病毒变异影响# | 1276.2 | Jun, 19 |
|  | (#CNBG’s COVID-19 inactivated vaccines are not affected by virus mutation#) |  |  |
| 17 | #我国三个新冠疫苗完成二期临床试验# | 18000 | Jun, 20 |
|  | (#Three COVID-19 vaccines in China have completed Phase II clinical trial#) |  |  |
| 18 | #国产新冠灭活疫苗启动国际临床Ⅲ期试验# | 84.2 | Jun, 23 |
|  | (#Domestic COVID-19 inactivated vaccines launches international clinical Phase Ⅲ trial# |  |  |
| 19 | #新冠病毒灭活疫苗生产车间在武汉落成# | 7388.4 | Jul, 2 |
|  | (#Production workshop of COVID-19 inactivated vaccines is completed in Wuhan#) |  |  |
| 20 | #世卫要求新冠疫苗保护期至少6个月# | 56.8 | Jul, 3 |
|  | (#WHO requires the protection period of the COVID-19 vaccines to be at least 6 months#) |  |  |
| 21 | #中国新冠疫苗2期临床试验取得良好结果# | 131 | Jul, 21 |
|  | (#The Phase II clinical trial of COVID-19 vaccines in China achieves good results #) |  |  |
| 22 | #新冠疫苗年底或可上市# | 345.4 | Jul, 22 |
|  | (#COVID-19 vaccines may be available at the end of the year#) |  |  |
| 23 | #全球首次正式发表新冠疫苗Ⅱ期临床数据# | 238.3 | Jul, 23 |
|  | (#World first officially releases Phase II clinical data of COVID-19#) |  |  |
| 24 | #新冠疫苗定价将不超40美元# | 888.9 | Jul, 29 |
|  | (#The price of COVID-19 vaccines will not exceed $40#) |  |  |
| 25 | #俄罗斯新冠疫苗将免费接种# | 20000 | Aug, 1 |
|  | (#COVID-19 vaccines will be free of charge in Russia#) |  |  |
| 26 | #首个新冠灭活疫苗生产车间通过安全检查# | 44.9 | Aug, 5 |
|  | (#The first production workshop of COVID-19 inactivated vaccines passed safety inspection# |  |  |
| 27 | #北京新冠灭活疫苗生产车间可随时投产# | 45.5 | Aug, 5 |
|  | (#Production workshop of COVID-19 inactivated vaccines in Beijing can be put into production at any time#) |  |  |
| 28 | #进入三期临床新冠疫苗半数来自中国# | 150.6 | Aug, 7 |
|  | (#Half of the COVID-19 vaccines in Phase III clinical trial come from China#) |  |  |
| 29 | #盖茨要求新冠疫苗定价低于3美元# | 963.7 | Aug, 8 |
|  | (#Gates required the COVID-19 vaccines to be priced below $3#) |  |  |
| 30 | #研究所辟谣新冠疫苗498元一支# | 127.4 | Aug, 13 |
|  | (#Research Institute refutes the rumor of COVID-19 vaccines that 498 yuan an injection#) |  |  |
| 31 | #新冠疫苗尚未上市# | 3 | Aug, 13 |
|  | (#COVID-19 vaccines not yet be available#) |  |  |
| 32 | #新冠灭活疫苗两针不到一千元# | 3081.6 | Aug, 18 |
|  | (#Two injections of COVID-19 inactivated vaccines cost less than one thousand yuan#) |  |  |
| 33 | #国产新冠灭活疫苗预计12月底上市# | 131.6 | Aug, 18 |
|  | (#Domestic COVID-19 inactivated vaccines are expected to be available at the end of December#) |  |  |
| 34 | #俄第二款新冠疫苗开始2期临床试验# | 70.1 | Aug, 18 |
|  | (#Second COVID-19 vaccine starts Phase II clinical trial in Russia #) |  |  |
| 35 | #新冠疫苗两针1000元太贵# | 24.2 | Aug, 19 |
|  | (#Two injections of COVID-19 vaccines cost one thousand yuan, which is too expensive#) |  |  |
| 36 | #新冠疫苗只能以成本作为定价依据# | 17000 | Aug, 23 |
|  | (#The price of COVID-19 vaccines can only be based on cost#) |  |  |
| 37 | #新冠疫苗如何定价# | 254.2 | Aug, 23 |
|  | (#How to price COVID-19 vaccines#) |  |  |
| 38 | #中国生物称新冠疫苗很可能年底上市# | 354.6 | Aug, 23 |
|  | (#CNBG declared COVID-19 vaccines were likely to be available at the end of the year#) |  |  |
| 39 | #卫健委称新冠疫苗价格比两针一千元低# | 65.9 | Aug, 23 |
|  | (National Health Commission claimed that the price of COVID-19 vaccines was lower than two injections of one thousand yuan#) |  |  |
| 40 | #国产新冠灭活疫苗实物首次亮相# | 19000 | Sept, 5 |
|  | (#Domestic COVID-19 inactivated vaccines first appears#) |  |  |
| 41 | #国产新冠灭活疫苗实物亮相服贸会# | 5766.4 | Sept, 6 |
|  | (#Domestic COVID-19 inactivated vaccines appears in CIFTIS#) |  |  |
| 42 | #新冠疫苗根据消费者支付能力等因素定价# | 134.5 | Sept, 7 |
|  | (#COVID-19 vaccines are priced based on factors such as consumers' ability to pay# |  |  |
| 43 | #国产新冠灭活疫苗接种数十万人零感染# | 2102.4 | Sept, 11 |
|  | (#Domestic COVID-19 inactivated vaccines inoculate hundreds of thousands of people with zero infection#) |  |  |
| 44 | #牛津疫苗志愿者出现副作用# | 769.1 | Sept, 9 |
|  | (#Volunteers of Oxford vaccines have side effects#) |  |  |
| 45 | #美辉瑞公司新冠疫苗出现副作用# | 17000 | Sept, 16 |
|  | (#COVID-19 vaccines produced by Pfizer in America have side effects#) |  |  |
| 46 | #新冠灭活疫苗有望年底上市# | 4319.2 | Sept, 18 |
|  | (#COVID-19 inactivated vaccines are expected to be available at the end of the year#) |  |  |
| 47 | #新冠疫苗中国定价会在大众接受范围内# | 11000 | Sept, 20 |
|  | (#Pricing of COVID-19 vaccines in China will be within the scope of public acceptance#) |  |  |
| 48 | #新冠灭活疫苗离成功只差最后一公里# | 23000 | Sept, 23 |
|  | (#COVID-19 inactivated vaccines are only one kilometer away from success#) |  |  |
| 49 | #国产新冠疫苗两针600元# | 7737 | Sept, 23 |
|  | (#Two injections of domestic COVID-19 vaccines cost six hundred yuan#) |  |  |
| 50 | #国内新冠疫苗定价出炉# | 4413.4 | Sept, 23 |
|  | (#Pricing of domestic COVID-19 vaccines is released#) |  |  |
| 51 | #中国4个新冠疫苗进入三期临床试验# | 428.6 | Sept, 25 |
|  | (#Four COVID-19 vaccines in China enter Phase III clinical trial#) |  |  |
| 52 | #日本决定免费为国民接种新冠疫苗# | 1406.8 | Oct, 2 |
|  | #Japan has decided to vaccinate its citizens against COVID-19 for free# |  |  |
| 53 | #中国正式加入新冠肺炎疫苗实施计划# | 23000 | Oct, 9 |
|  | (#China formally joins COVAX#) |  |  |
