# Supplementary material for: Unfolding the Determinants of COVID-19 Vaccine Acceptance in China
Source: J Med Internet Res. 2021 Jan 15;23(1):e26089. doi: 10.2196/26089 (PMC7813210; doi:10.2196/26089)
Supplement: Multimedia Appendix 2 [file jmir_v23i1e26089_app2.docx]

|  | Key words in Chinese | Key words in English | The weight |
| --- | --- | --- | --- |
| Synonym for “Expensive” | 贵 | Expensive | 0.5 |
|  | 不接受 | Not accept | 1 |
|  | 买不起 | Can not afford | 0.6 |
|  | （价格）高 | (Price) high | 0.2 |
|  | 不菲 | Expensive | 0.8 |
|  | 昂贵 | Expensive | 1 |
| Synonym for “Cheap” | 不贵 | Not expensive | -0.5 |
|  | 便宜 | Cheap | -1 |
|  | 接受 | Accept | -0.2 |
|  | （价格）不高 | (Price) not high | -0.4 |
|  | 廉价 | Cheap | -1 |
|  | 划算 | Worth to buy | -0.8 |

**Price polarity table**
