# Supplementary material for: Unfolding the Determinants of COVID-19 Vaccine Acceptance in China
Source: J Med Internet Res. 2021 Jan 15;23(1):e26089. doi: 10.2196/26089 (PMC7813210; doi:10.2196/26089)
Supplement: Multimedia Appendix 3 [file jmir_v23i1e26089_app3.docx]

**Discussions, forwards, comments, likes related to China’s five vaccine types.**

|  | Inactivated vaccines | Adenovirus vector vaccines | Recombinant protein vaccines | Nucleic acid vaccines | vaccines using attenuated influenza viruses as vectors |
| --- | --- | --- | --- | --- | --- |
| Discussions (n/N) | 588/1117  (53%) | 139/1117  (12%) | 165/1117  (15%) | 138/1117  (12%) | 87/1117  (8%) |
| Forwards (n/N) | 3072/7332  (42%) | 1755/7332  (24%) | 1300/7332  (18%) | 919/7332  (12%) | 286/7332  (4%) |
| Comments  (n/N) | 3671/6547  (56%) | 808/6547  (12%) | 970/6547  (15%) | 791/6547  (12%) | 307/6547  (5%) |
| Likes  (n/N) | 17940/37068  (49%) | 3630/37068  (10%) | 6790/37068  (18%) | 6012/37068  (16%) | 2696/37068  (7%) |
