# Supplementary material for: Unfolding the Determinants of COVID-19 Vaccine Acceptance in China
Source: J Med Internet Res. 2021 Jan 15;23(1):e26089. doi: 10.2196/26089 (PMC7813210; doi:10.2196/26089)
Supplement: Multimedia Appendix 4 [file jmir_v23i1e26089_app4.pdf]

Statistical Report on Internet Development in China

(September 2020)

CNNIC
中国互联网络信息中心
CHINA INTERNET NETWORK INFORMATION CENTER

Preface

In 1997, China's competent departments authorized China Internet Network Information Center (CNNIC) to organize relevant Internet entities to jointly carry out the Statistical Survey on Internet Development in China and regularly release the *Statistical Report on Internet Development in China* (hereinafter referred to as the "Report") at the beginning and middle of each year. Ever since then, CNNIC has published 45 reports. The Report has reflected the process of building up China's strength in cyberspace through core data. It has provided an important reference for Chinese government departments, domestic and international industry institutions, experts, and scholars to understand the development of China's Internet and formulate relevant policies.

The year of 2020 marks the closing year for finishing building a moderately prosperous society in all respects and winning the battle against poverty, the final year of the 13th Five-Year Plan, and the preparation year of the 14th Five-Year Plan. In the first half of 2020, China's Internet industry demonstrated tremendous vitality and resilience, overcoming the impact and difficulties brought about by the COVID-19 epidemic. Remarkable progress was made in digital infrastructure, digital economy, digital benefits for the people, and digital governance. The Internet industry has become an important pillar of China to address new challenges and build a new economy. As a faithful recorder of implementing the national strategy for cyber development, CNNIC has followed the development of China's Internet, expanding the scope of research and subdividing research areas. The Report focuses on the five aspects of basic Internet development, size and structure of Internet users, development of Internet applications, development of e-government, and Internet security. From a multi-pronged perspective, CNNIC has worked to comprehensively demonstrate the development of China's Internet in the first half of 2020 through all-round data.

Here, we hereby express our heartfelt thanks to the Office of the Central Cyberspace Affairs Commission, the Ministry of Industry and Information Technology of PRC, the National Bureau of Statistics of China, the Central Committee of the Communist Youth League and other departments and units for their guidance and support for the Report. We would also like to express our sincere thanks to the E-Government Research Center of the Party School of the CPC Central Committee (National Academy of Governance), other institutions and Internet users that have supported this statistical survey on the Internet development.

China Internet Network Information Center (CNNIC)
September 2020

CONTENTS

SUMMARY 1

CHAPTER ONE BASIC INTERNET DEVELOPMENT 3

I. Basic Internet Resources 3

(I) An Overview of Basic Internet Resources 3

(II) IP Address 3

(III) ccTLDs 4

II. Application of Internet Resources 5

(I) Websites 5

(II) Mobile Internet Access Traffic 6

(III) Number and Category of Apps 6

III. Internet Access Environment 8

(I) Internet Access Devices 8

(II) Online Duration 8

(III) Proportion of broadband subscribers of 100Mbps and above 10

(IV) Scale and Proportion of Fiber Broadband Users 11

(V) Number of Cellular IoT Terminal Users 11

CHAPTER TWO SIZE AND STRUCTURE OF INTERNET USERS 13

I. The Size of Internet Users 13

(I) Overall Size of Internet Users 13

(II) The Size of Internet Users in Urban and Rural Areas 15

(III) Achievements in Internet-based poverty alleviation 15

(IV) The Size of Non-netizen 18

II. The Attribute Structure of Internet Users 19

(I) Gender Structure 19
(II) Age Structure 20
(III) Educational Background 20
(IV) Occupational Structure 21
(V) Income Structure 22

CHAPTER THREE THE DEVELOPMENT OF INTERNET APPLICATIONS 25

I. Overview of Internet Applications 25
II. Basic Apps 27
(I) Instant messaging 27
(II) Search engine 29
(III) Online news 32
(IV) Social networking applications 33
(V) Telecommuting Apps 35
III. Business Transaction Applications 37
(I) Online shopping 37
(II) Online meal ordering 39
(III) Online payment 41
IV. Online Entertainment Applications 43
(I) Online games 43
(II) Online video 46
(III) Live streaming 48
V. Public Service Applications 50
(I) Online Car-hailing Services 50
(II) Online education 52
(III) Online medical services 55

CHAPTER FOUR DEVELOPMENT OF E-GOVERNMENT 58

I. Development of E-government Services 58

II. Development of Government Websites 59

I. General Situation of Government Websites by Province 59

II. Number of Government Websites by Administrative Level 61

III. Number of Columns on Government Websites by Administrative Level 61

IV. The number of articles updated on the homepages of government websites at all administrative levels 62

III. The Development of New Government Media..63

(I) The Development of Government Service Search..63

(II) Development of Government Microblogs 64

(III) Development of Zhengwutoutiao and Douyin Accounts 66

CHAPTER FIVE THE INTERNET SECURITY 69

I. Cyber Incidents 69

(I) Proportion of Types of Cybersecurity Problems..69

(II) Proportion of Types of Internet Fraud...69

II. Website security incidents and information system vulnerabilities 70

(I) Number of Websites Tampered with by Hackers in China 70

(II) The Number of Websites Implanted with Backdoor Malware in China 71

(III) The Number of Information System Vulnerabilities..73

III. Reporting and Handling of Cybersecurity Incidents................................74

(I) Number of Reported Cybersecurity Incidents Received by CNCERT 74

(II) Number of Reports Received by China’s Network Reporting Departments at all levels 74

Summary

◇ As of June 2020, China had 940 million netizens, up by 36.25 million over March 2020[1], and its Internet penetration had reached 67.0%, up 2.5 percentage points over March 2020.

◇ Up to June 2020, the number of mobile Internet users in China had reached 932 million, up 35.46 million over March 2020. The proportion of China's netizens accessing the Internet via their mobile phones had amounted to 99.2%, roughly unchanged from March 2020.

◇ As of June 2020, the size of rural Internet users was 285 million or 30.4% of China's total netizen population, up 30.63 million over March 2020, while that of urban netizens had reached 654 million or 69.6% of China's total, up 5.62 million from March 2020.

◇ As of June 2020, the proportions of Chinese netizens accessing the Internet through mobile phones, desktop computers, laptops computers, TVs and tablet computers were 99.2%, 37.3%, 31.8%, 28.6% and 27.5%, respectively.

◇ Up to June 2020, the number of IPv6 addresses in China had reached 50,903 blocks/32.

◇ As of June 2020, the number of the country code top-level domain (ccTLD) ".CN" stood at 23.04 million, up 2.8% from the end of 2019.

◇ Up to June 2020, the user size of instant messaging was 931 million or 99.0% of China's total netizen population, up 34.66 million over March 2020; the number of mobile instant messaging users had reached 930 million, up 40.24 million from March 2020, making up 99.8% of mobile Internet users.

◇ As of June 2020, the user size of search engine was 766 million or 81.5% of China's total netizen population, up 15.39 million over March 2020; the number of mobile search engine users had reached 761 million, up 15.42 million from March 2020, accounting for 81.6% of mobile Internet users.

◇ Up to June 2020, the user size of online shopping was 749 million or 79.7% of China's total netizen population, up 39.12 million over March 2020; the number of mobile shopping users had

[1] Due to the COVID-19 epidemic, the deadline for telephone survey of the 45th Report was March 15, 2020, so the data collection ended in March 2020.

amounted to 747 million, up 39.47 million from March 2020, taking up 80.1% of mobile Internet users.

◇ As of June 2020, the user size of online payment was 805 million or 85.7% of China's total netizen population, up 37.02 million over March 2020; the number of mobile payment users stood at 802 million, up 36.64 million from March 2020, representing 86.0% of mobile Internet users.

◇ Up to June 2020, the user size of online video (including video clips) in China had reached 888 million, up 37.77 million from March 2020, making up 94.5% of all Internet users. The number of video clip users amounted to 818 million, up 44.61 million from March 2020, accounting for 87.0% of overall Internet users.

◇ As of June 2020, the user size of live streaming in China had reached 562 million, up 2.48 million from March 2020, taking up 59.8% of all Internet users. Specifically, the user size of live-stream e-commerce was 309 million, up 44.30 million from March 2020, accounting for 32.9% of overall Internet users.

◇ Up to June 2020, the number of users of China's e-government services was 773 million, up 78.89 million from March 2020, making up 82.2% of all Internet users.

◇ As of June 2020, 381 million Internet users or 40.5% of all netizens had received online education services in China.

◇ Up to June 2020, 276 million Internet users or 29.4% of all netizens had received online medical services in China.

◇ As of June 2020, the number of e-commuting users in China amounted to 199 million, or 21.2% of overall Internet users.

Chapter One Basic Internet Development

I. Basic Internet Resources

(I) An Overview of Basic Internet Resources

Up to June 2020, China had 38,907 million IPv4 addresses and 50,903 blocks/32 of IPv6 addresses. The number of China's ccTLD ".CN" was 23.04 million, up 2.8% from the end of 2019.

Table 1 Comparison — Basic Internet Resources from Dec. 2019 to Jun. 2020

	Dec. 2019	Jun. 2020	Semi-annual increment	Semi-annual growth rate
IPv4[2]	387,508,224	389,067,008	1,558,784	0.4%
IPv6[3] (block/32)	50,877	50,903	26	0.1%
Domain names ending with ".CN"	22,426,900	23,044,376	617,476	2.8%

(II) IP Address

Up to June 2020, the number of IPv6 addresses had amounted to 50,903 blocks/32, up 0.1% over the end of 2019.

[2]The data cover Hong Kong, Macao and Taiwan.
[3]The data cover Hong Kong, Macao and Taiwan.

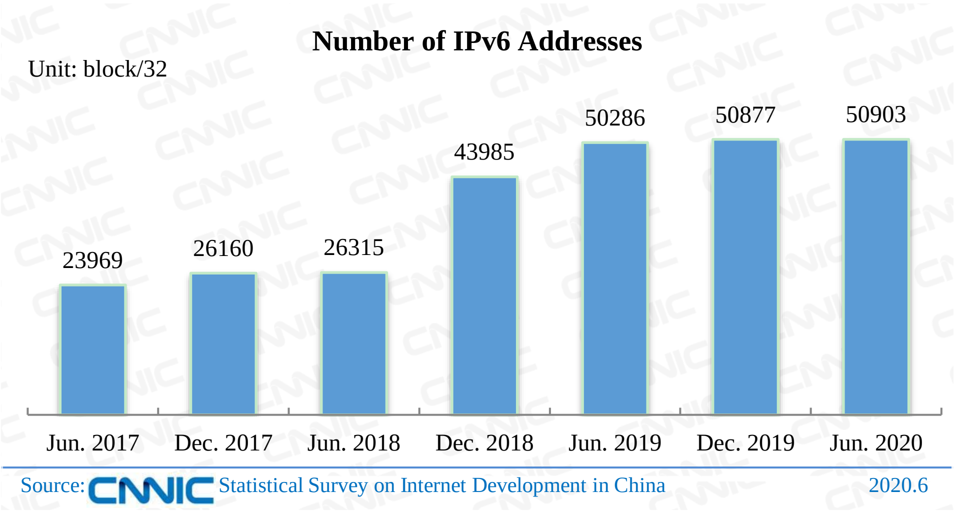

Figure 1 Number of IPv6 Addresses[4]

Up to June 2020, the number of IPv4 addresses was registered at 389.07 million.

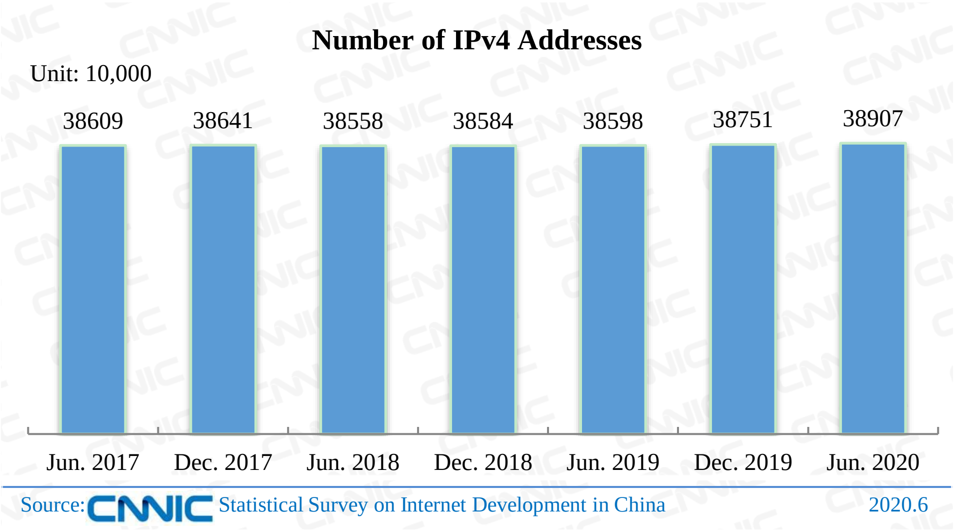

Figure 2 Number of IPv4 Addresses[5]

(III) ccTLDs

As of June 2020, the number of domain names ending with ".CN" in China was 23.04 million, up 2.8% from the end of 2019, while that of domain names ending with ".中国" was 1.7 million.

[4]The data in Figure 2 cover Hong Kong, Macao and Taiwan.
[5]The data in Figure 2 cover Hong Kong, Macao and Taiwan.

Table 2 Number of Domain Names Ending with ".CN" by Category

	Number	Proportion in total ".CN" domain names
.CN	20,046,906	87.0%
.COM.CN	2,359,443	10.2%
.NET.CN	329,090	1.4%
.ORG.CN	160,670	0.7%
.ADM.CN	108,903	0.5%
.GOV.CN	19,373	0.1%
.AC.CN	13,468	0.1%
.EDU.CN	6,348	0.0%
OTHERS	175	0.0%
Total	23,044,376	100.0%

II. Application of Internet Resources

(I) Websites

As of June 2020, there were 4.68 million websites[6] in China, down 5.8% from the end of 2019.

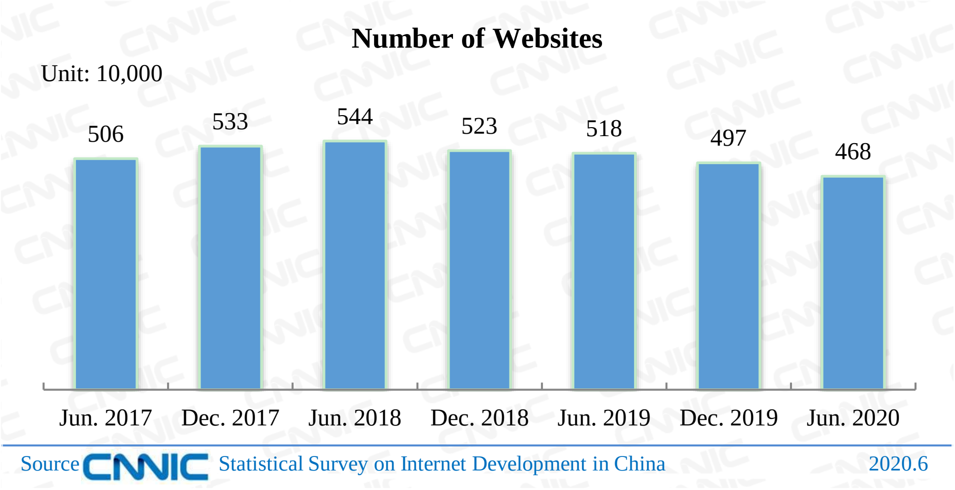

Figure 3 Number of Websites[7]

Up to June 2020, China had 3.19 million websites with domain names ending with ".CN", down 6.3% from the end of 2019.

[6] The websites whose domain name registrants are within the territory of the P.R.C.
[7] The number of websites does not include that of those ending with ".EDU.CN".

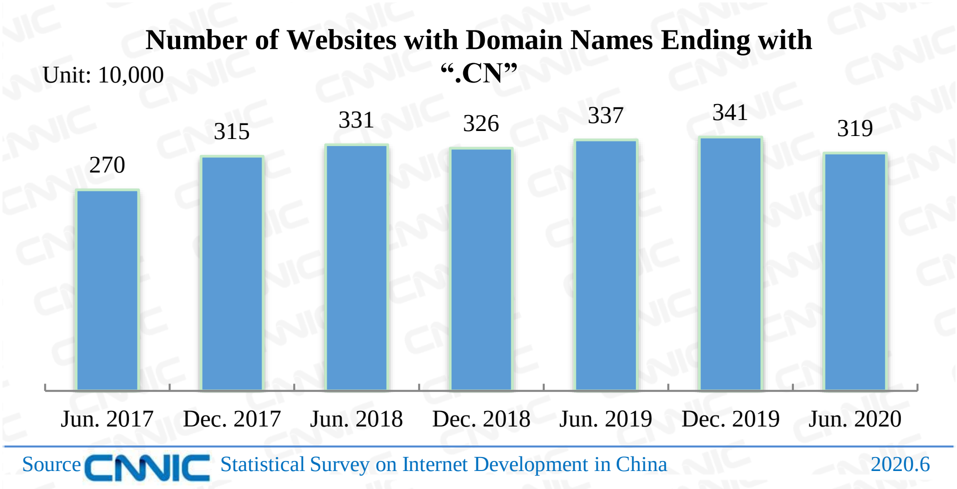

Figure 4 Number of Websites with Domain Names Ending with ".CN"[8]

(II) Mobile Internet Access Traffic

From January to June 2020, the cumulative mobile Internet traffic totaled 74.5 billion GB, up 34.5% year on year.

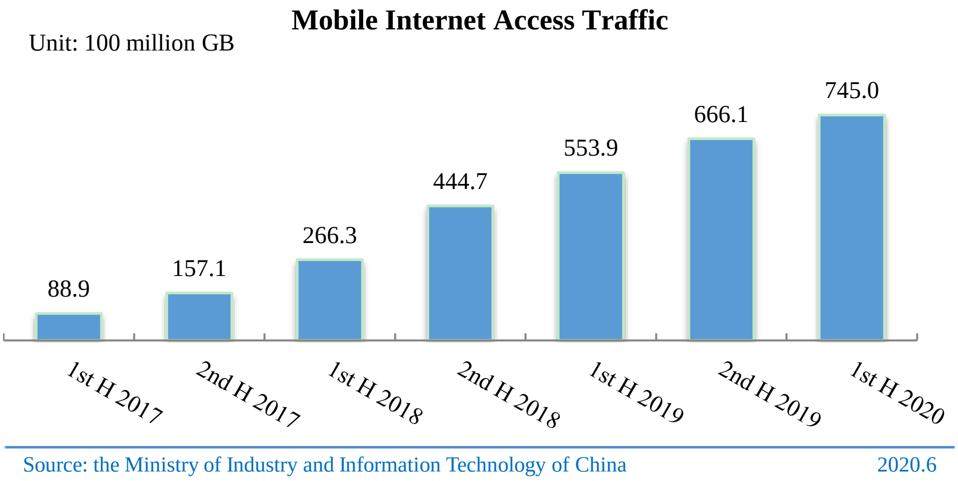

Figure 5 Mobile Internet Access Traffic

(III) Number and Category of Apps

As of June 2020, the number of Apps (Application, or mobile Internet application) monitored in China's domestic market was 3.59 million, down 80,000 or 2.2% from the end of 2019.

[8]The number of websites ending with ".CN" does not include that of those ending with ".EDU.CN".

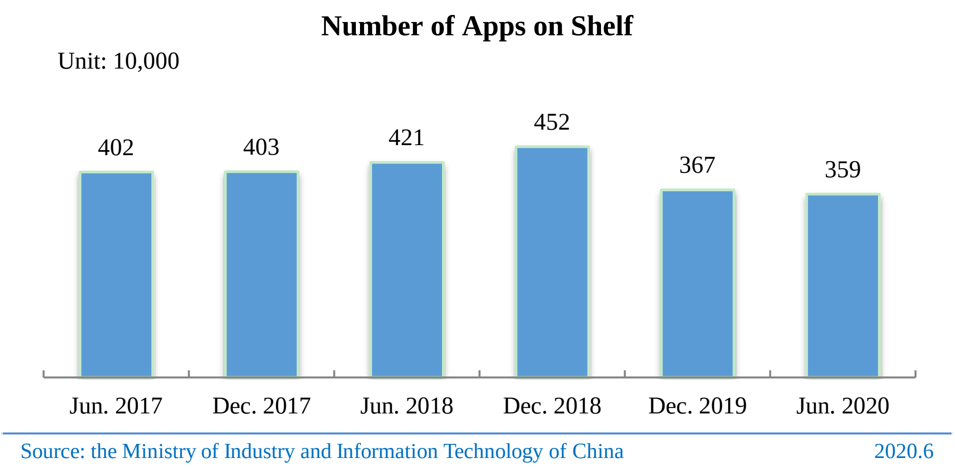

Figure 6 Number of Apps on Shelf[9]

As of June 2020, the proportion of Apps in the top four categories (games, daily tools, e-commerce, and consumer services) by mobile App size accounted for 58.6% of the total. Specifically, the number of game Apps reached 925,000, accounting for 25.8% of all Apps, an increase of 16,000 compared to the end of 2019; that of daily tools, e-commerce and consumer service Apps reached 508,000, 365,000 and 305,000 respectively and ranked second, third and fourth in the scale of mobile Apps, representing 14.1%, 10.2% and 8.5% of all respectively; and other Apps such as social communication and education made up 41.4% of the total.

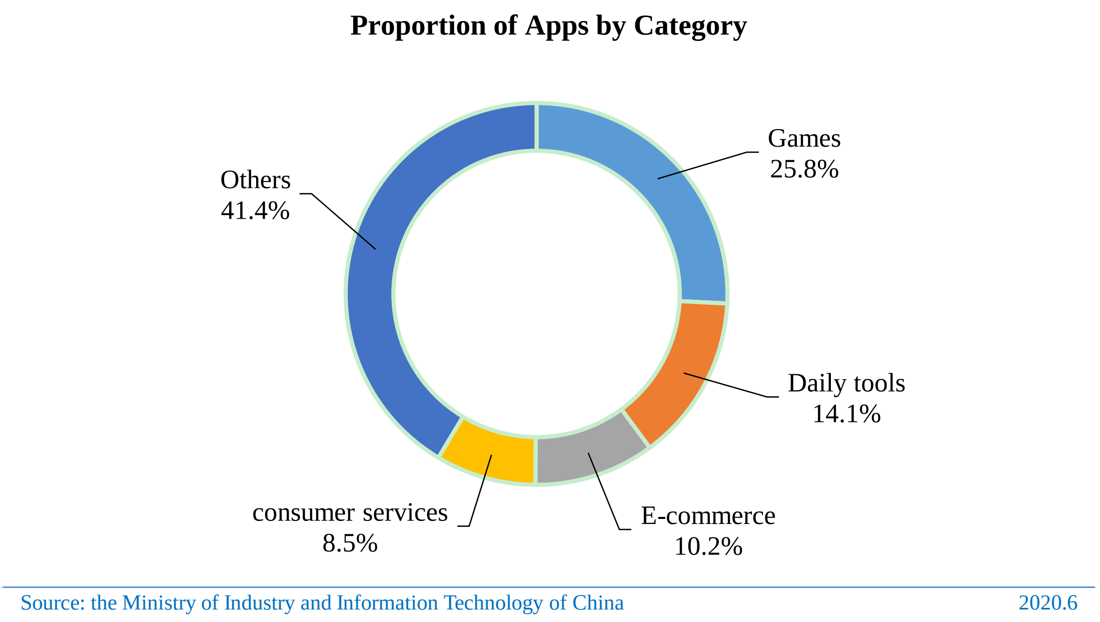

Figure 7 Proportion of Apps by Category

[9] On shelf: from 2019 onwards, the method of monitoring data would be shifted from a cumulative strategy (i.e. the statistics are calculated cumulatively) to an on-shelf strategy (i.e. the statistics are only for on-shelf) to more accurately reflect the mobile App market dynamics.

III. Internet Access Environment

(I) Internet Access Devices

As of June 2020, the proportion of Chinese Internet users using mobile phones to access the Internet reached 99.2%, basically unchanged from March 2020; the proportions of netizens using desktop PCs, laptops, TVs and tablet PCs to do so were 37.3%, 31.8%, 28.6% and 27.5% respectively.

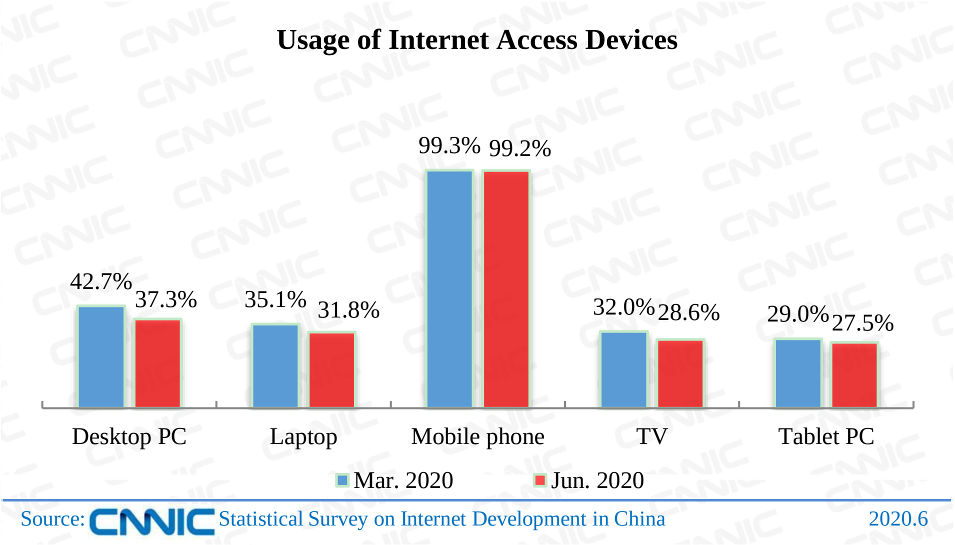

Figure 8 Usage of Internet Access Devices

(II) Online Duration

1. Per Capita Weekly Online Duration of Internet Users

As of June 2020, the per capita weekly online duration[10] of China's Internet users was 28.0 hours, down 2.8 hours over March 2020.

[10]Per capita weekly online duration refers to the average daily number of hours of accessing the Internet multiplied by 7 days in a week in the past six months.

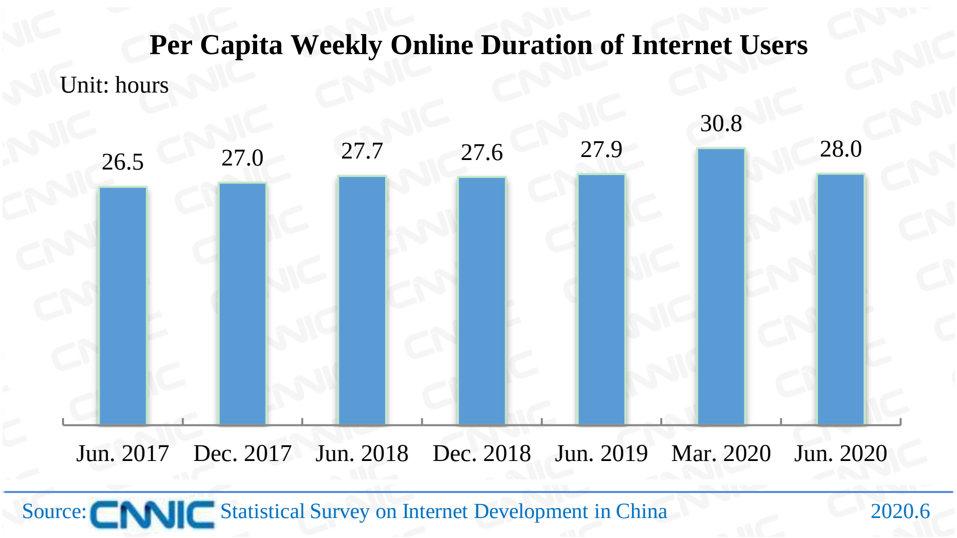

Figure 9 Per Capita Weekly Online Duration of Internet Users

2. Proportion of Usage Duration by Type of Application

In June 2020, among all Apps frequently used by mobile Internet users, instant messaging Apps were used for the longest duration, accounting for 13.7%; online video, online audio[11], video clip, online music and live streaming Apps ranked from the second to sixth in terms of usage duration, making up 12.8%, 10.9%, 8.8%, 8.1% and 7.3%, respectively.

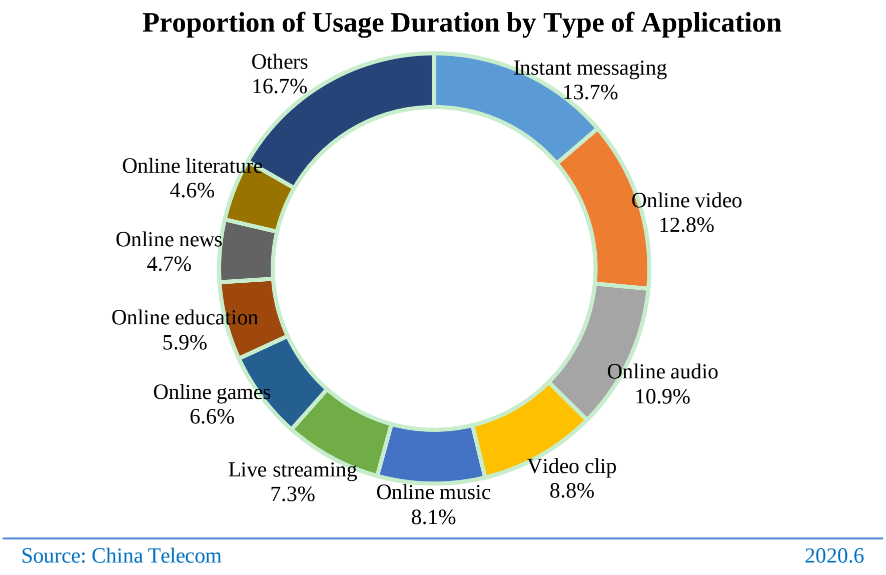

Figure 10 Proportion of Usage Duration by Type of Application[12]

---

[11] Online audio refers to the type of mobile Internet applications that can provide audio programs such as network radio stations.

[12] Source: China Telecom. The indicators are based on mobile phone users' online log data and China Telecom's App tag data in June. The total average daily duration of each type of Apps is calculated by building a data model.

3. Distribution of Usage Periods by Type of Application

In June 2020, of the six types of Apps commonly used by mobile phone users, the usage periods of instant messaging, online shopping and online news Apps were evenly distributed, accounting for over 75% of the total usage period from 8: 00 to 21: 00. Online meal ordering had obvious time characteristics, with peak usage ranging from 11: 00 to 12: 00 and from 17: 00 to 20: 00, making up 53.8% of the total duration. Video clips peaked from 11: 00 to 13: 00 and 17: 00 to 22: 00 respectively, accounting for 53.7% of the total duration. Live streaming was even more obvious in concentrated periods, with the usage period between 18: 00 and 23: 00, accounting for over 40% in total.

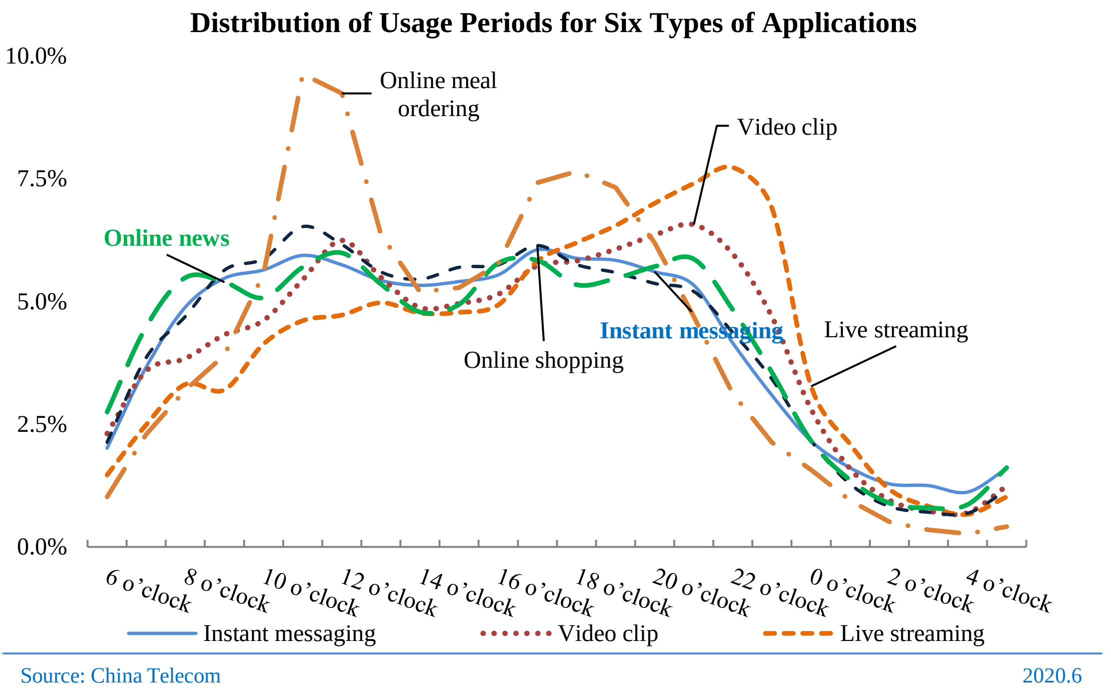

Figure 11 Distribution of Usage Periods[13] for Six Types of Applications

(III) Proportion of broadband subscribers of 100Mbps and above

As of June 2020, the number of fixed broadband subscribers with the access speed of 100Mbps and above accounted for 86.8% of the total.

[13] Distribution of usage period refers to the period distribution of usage duration of Apps in all fields. For example, if a user uses an instant messaging App for 15 minutes or 0.25 hours during the period from 6 o'clock to 7 o'clock, then the duration of using the application is 4 hours throughout the entire day. The calculation method is 0.25/4.

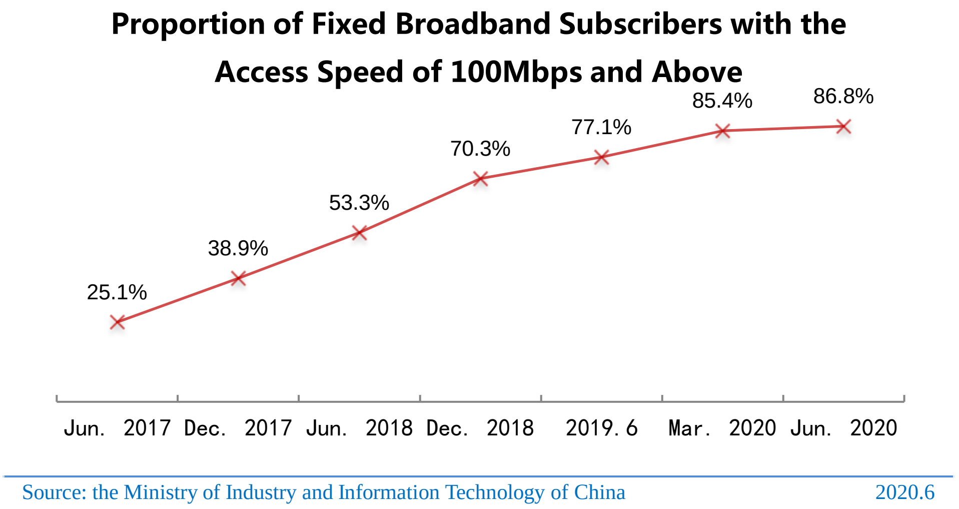

Figure 12 Proportion of Fixed Broadband Subscribers with the Access Speed of 100Mbps and Above

(IV) Scale and Proportion of Fiber Broadband Users

As of June 2020, the number of FTTH/O[14] users had reached 433.58 million, accounting for 93.2% of all Internet broadband subscribers, up 0.3 percentage points from the end of 2019.

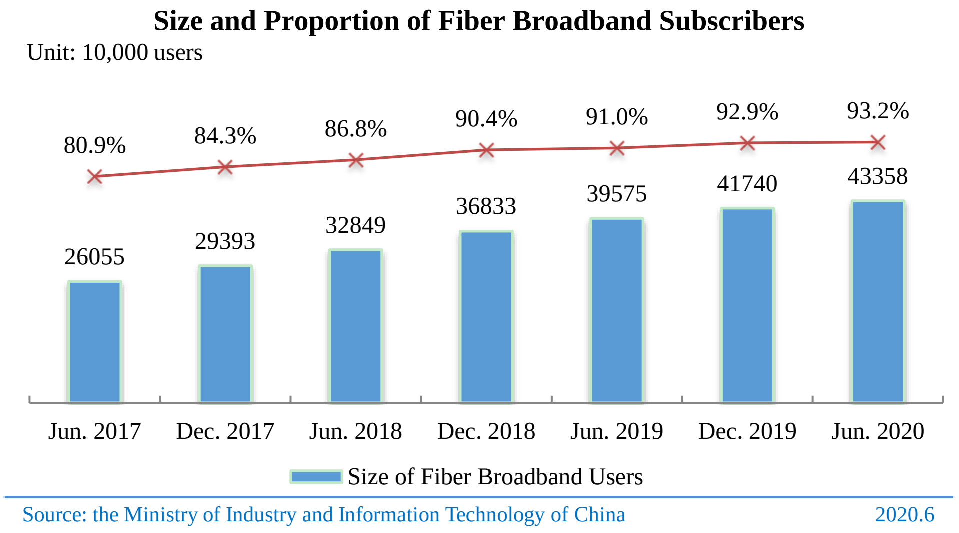

Figure 13 Size and Proportion of Fiber Broadband Subscribers

(V) Number of Cellular IoT Terminal Users

[14] FTTH/O refers to FTTH and FTTO. FTTH means Fiber to the home. FTTO stands for Fiber to the office.

As of June 2020, the three basic telecom companies developed 1.106 billion cellular IoT terminal[15] users, a net increase of 78.12 million from the end of 2019. Terminal users specializing in smart manufacturing, smart transportation and smart public utilities accounted for 21.1%, 18.2% and 21.4%, respectively.

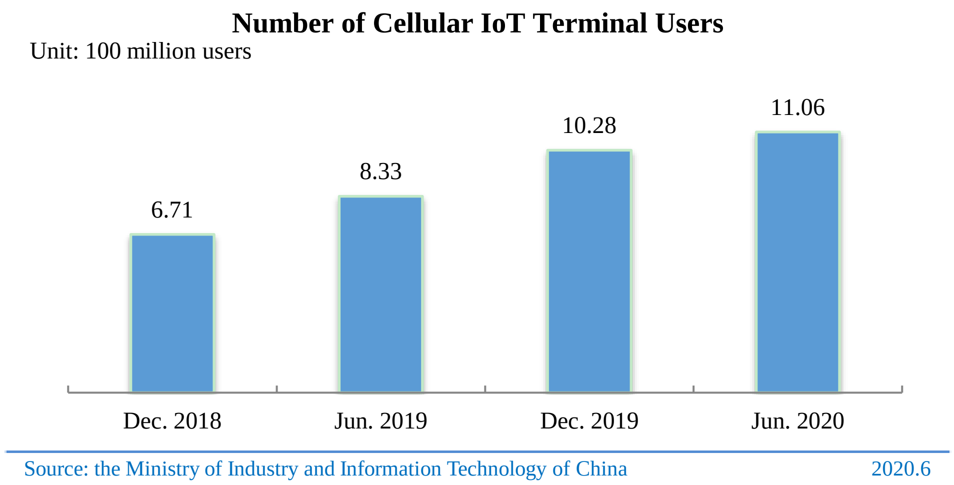

Figure 14 Number of Cellular IoT Terminal Users

[15]Cellular IoT terminal: IoT terminal accesses the GSM network (such as the GPRS network of China Mobile), integrates with the 2G mobile communication module, with a SIM card inserted into it, and exchanges data with background through GPRS network. Cellular IoT includes Narrowband Internet of Things (NB-IOT), Enhanced Machine Type Communication (eMTC), and others.

Chapter Two Size and Structure of Internet Users

I. The Size of Internet Users

(I) Overall Size of Internet Users

As of June 2020, China had 939.84 million netizens, up 36.25 million over March 2020, and its Internet penetration had reached 67.0%, up 2.5 percentage points over March 2020.

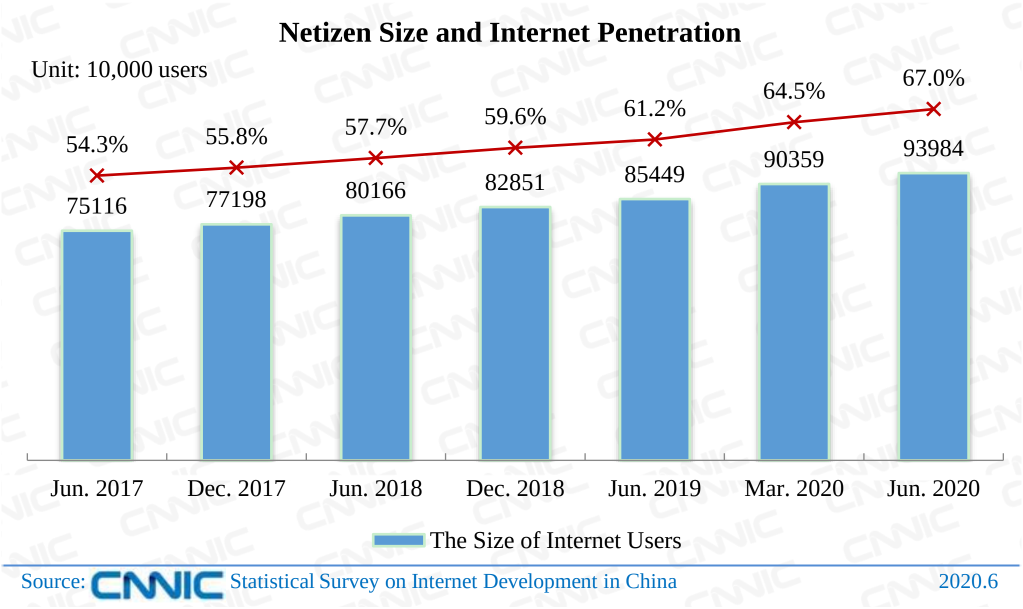

Figure 15 Netizen Size and Internet Penetration

Up to June 2020, the number of mobile Internet users in China had reached 932.36 million, up 35.46 million over March 2020. The proportion of Chinese netizens accessing the Internet via their mobile phones had amounted to 99.2%, roughly unchanged from March 2020.

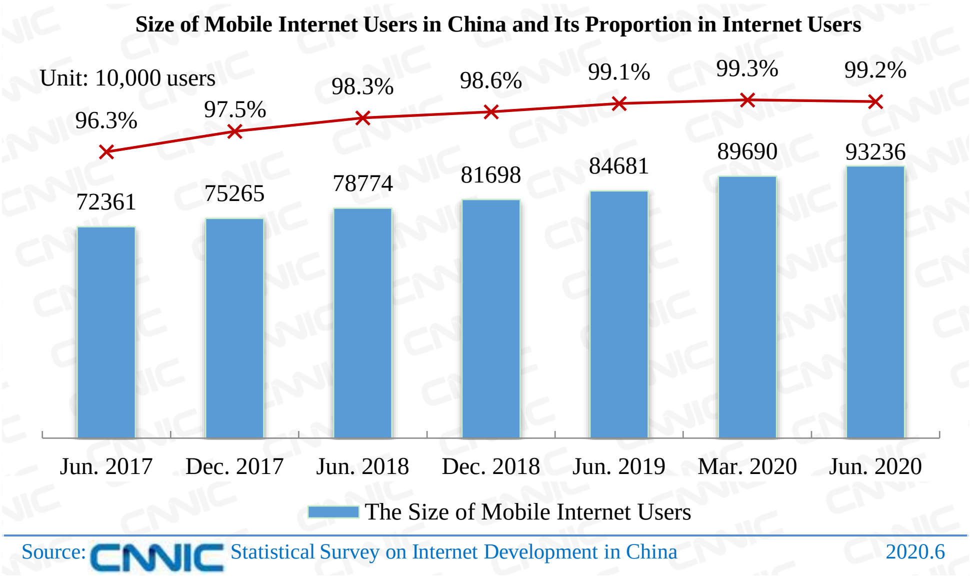

Figure 16 Size of Mobile Internet Users and Its Proportion in Internet Users

Since the 18th CPC National Congress, China's IT application has been developed by leaps and bounds, under the guidance of General Secretary Xi Jinping's important thoughts on a strong Internet nation. New infrastructure such as 5G and the industrial Internet has been rolled out, urban and rural broadband access has continued to improve, and Internet applications have continued to be enriched and refined. All these efforts have laid a solid foundation for China to respond effectively and timely to the COVID-19 epidemic. In the first half of 2020, China saw network infrastructure, the size of Internet users, Internet penetration record high despite the impact of adverse factors such as the epidemic. Up to June 2020, the number of Internet broadband access ports nationwide reached 931 million, up 3.1% year-on-year, with a net increase of 15.66 million from the end of 2019[16]. The number of Internet users reached 940 million, with an increase of 36.25 million from March 2020. The Internet penetration was 67.0%, up 2.5 percentage points over March 2020. The number of rural Internet users reached 285 million or 30.4% of all Internet users, up 30.63 million from March 2020. The gap between urban and rural Internet penetration was narrowed by 6.3 percentage points.

Against the backdrop of the ongoing COVID-19 pandemic and the complex international situation, China has been committed to fostering a development paradigm with domestic circulation as the mainstay and domestic and international circulations reinforcing each other, thanks to its increasingly sophisticated network infrastructure, a huge size of Internet users, and rich comprehensive Internet applications. All the advantages have empowered China to do a good job of keeping employment, the financial sector, foreign trade, foreign and domestic investments, and expectations stable as well as ensuring security in job, basic living needs, operations of market entities, food and energy security, stable industrial and supply chains, and the normal functioning of primary-level governments. By doing so, China will finish building a moderately prosperous society in all respects and ensure the successful completion of the 13th Five-Year Plan.

[16]Source: the *Economic Operation of Communication Industry in the First Half of 2020* by the Ministry of Industry and Information Technology of China.

(II) The Size of Internet Users in Urban and Rural Areas

As of June 2020, the size of rural Internet users was 285 million or 30.4% of China's total netizen population, up 30.63 million over March 2020, while that of urban Internet users had reached 654 million or 69.6% of China's total netizen population, up 5.62 million from March 2020.

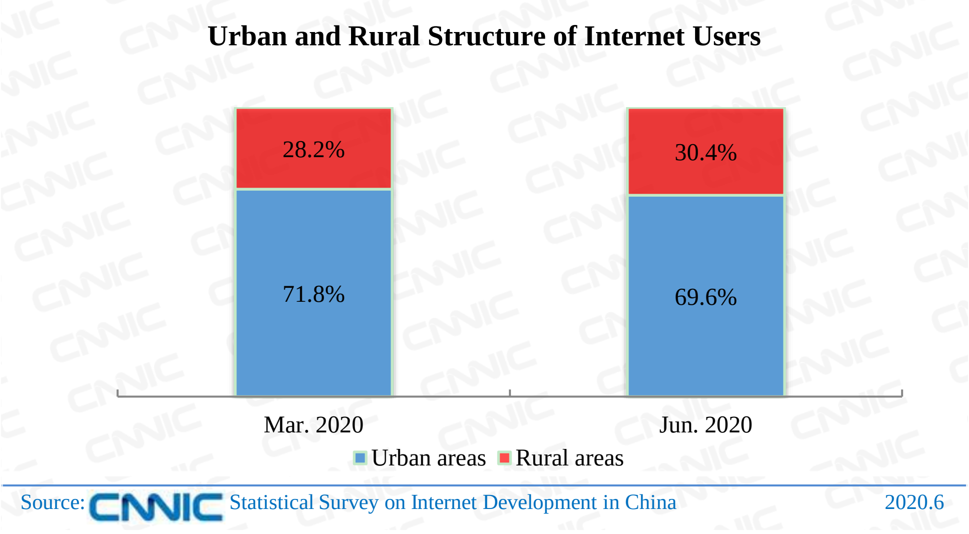

Figure 17 Urban and Rural Structure of Internet Users

Up to June 2020, the Internet penetration in China's urban areas was 76.4%, basically unchanged from March 2020, while that in rural areas was 52.3%, up 6.1 percentage points over March 2020. The gap of Internet penetration between urban and rural areas was narrowed by 6.3 percentage points.

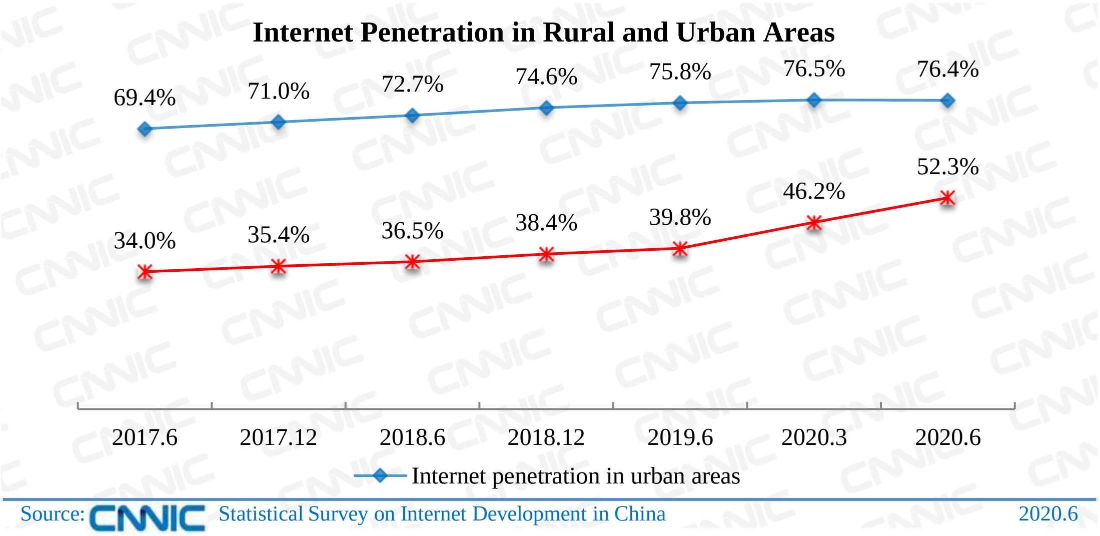

Figure 18 Internet Penetration in Rural and Urban Areas

(III) Achievements in Internet-based poverty alleviation

The year of 2020 marks that the goal of finishing building a moderately prosperous society in all

respects will be met and that China will win the battle against poverty. Under the leadership of the Central Committee of the Communist Party of China with Comrade Xi Jinping at the core, the Chinese people have resolutely overcome the difficulties and challenges posed by the COVID-19 epidemic and are determined to realize poverty alleviation. Internet-based poverty alleviation, an important means in this regard, has been increasingly understood, engaged and recognized by Internet users.

First, Internet users' awareness of various online poverty alleviation activities has been further raised. As of June 2020, the proportion of Internet users who saw the "promotion of distinctive agricultural products from poor areas" on the Internet reached 51.5%, up 3.4 percentage points over March 2020.

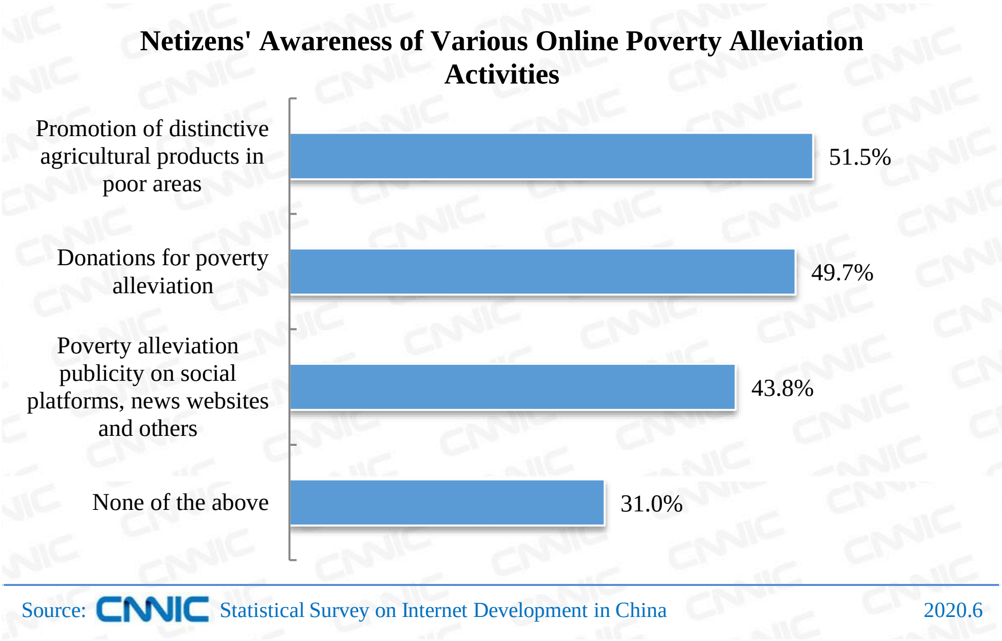

Figure 19 Netizens' Awareness of Various Online Poverty Alleviation Activities

Second, more Internet users have engaged in various online poverty alleviation activities. According to the data, as of June 2020, 52.7% of Internet users who understood online poverty alleviation activities participated in praising, forwarding and commenting on poverty alleviation campaigns, up 16.4 percentage points from March 2020; 47.1% participated in online poverty alleviation donations, up 3.2 percentage points over March 2020; and the proportion of Internet users who purchased distinctive agricultural products from poor areas online reached 34.6%, up 11.5 percentage points from March 2020.

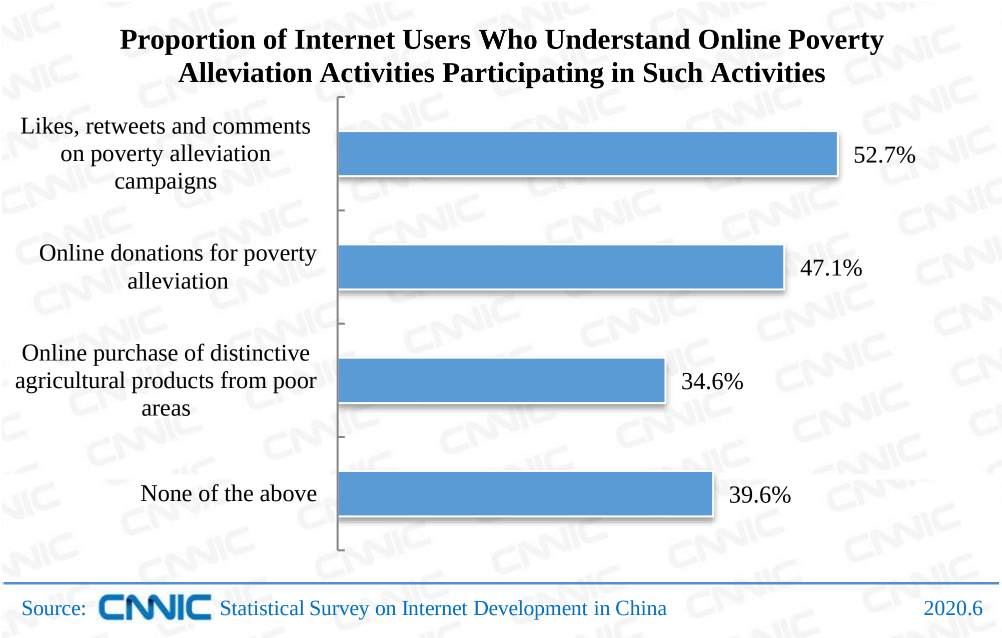

Figure 20 Proportion of Internet Users Who Understand Online Poverty Alleviation Activities Participating in Such Activities

Thirdly, more Internet users have recognized the role of the Internet in helping lift poor areas out of poverty. As of June 2020, the proportion of Internet users who agree that the Internet can "pool the strength of netizens to help the needy" had reached 81.2%, up 1.7 percentage points from March 2020; that of those users who agree that the Internet can "help the poor expand the sales of agricultural products through e-commerce" amounted to 77.2%, up 1.8 percentage points over March 2020; that of Internet users who agree that the Internet can "make it easier for needy people to obtain information on job, social security and medical care" reached 75.9%, up 3.7 percentage points from March 2020; and that of Internet users who agree that the Internet can "provide children in poor areas with quality learning resources through distance education" was 74.0%, up 4.4 percentage points from March 2020.

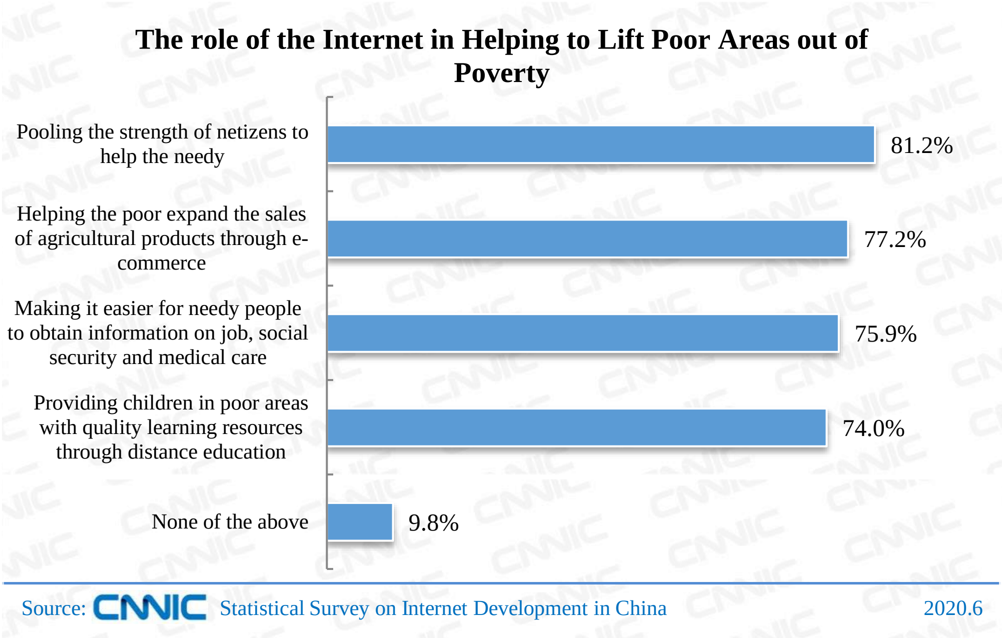

Figure 21 The Role of the Internet in Helping to Lift Poor Areas out of Poverty

(IV) The Size of Non-netizen

As of June 2020, of 463 million non-netizens in China, these in urban areas accounted for 43.8%, while those in rural areas made up 56.2%. Most non-netizens are still living in rural areas.

While focusing on the growth in the number of Internet users, we still need to pay attention to the non-netizens community. During the COVID-19 epidemic, no access to the Internet prevented non-netizens from getting more timely and effective epidemic control information and enjoying the convenience of travel and shopping services delivered by the Internet. Shortage of skills, limited literacy level and inadequate devices are major reasons why non-netizens do not access the Internet. According to the data, 48.9% of non-netizens did not access the Internet because they did not know how to use the computer/Internet; 18.2% did not because they did not master Pinyin or due to literacy limitations; 14.8% did not because they did not have access to computers and other devices; 12.9% did not because they were too old/too young to access the Internet; and the proportion of non-netizens having no time to surf the Internet or being not interested in it was less than 10%.

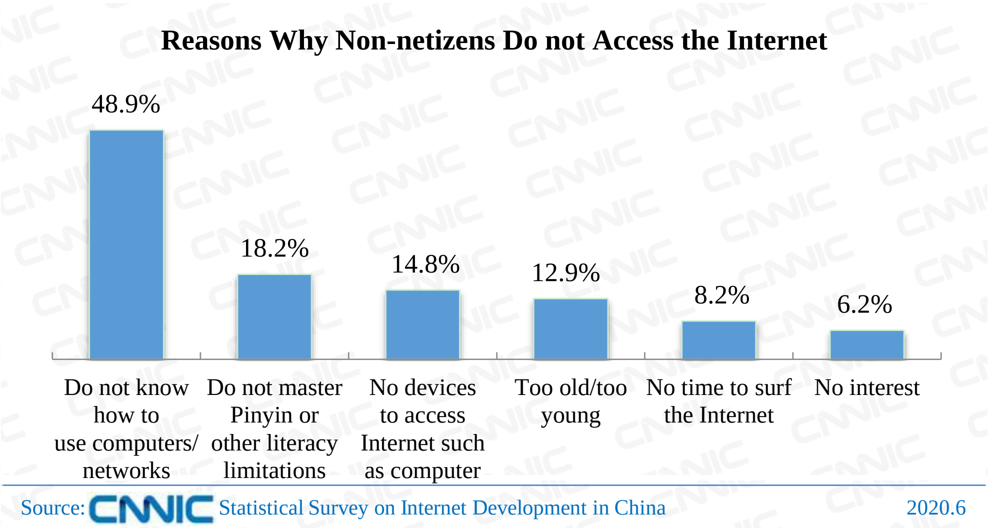

Figure 22 Reasons Why Non-netizens Do not Access the Internet

According to the data, the primary factor for non-netizens access the Internet was the convenience of communicating with their family members, accounting for 31.8%, followed by the availability of barrier-free Internet devices, making up 30.1%, and the provision of free relevant training and guidance, taking up 30.0%.

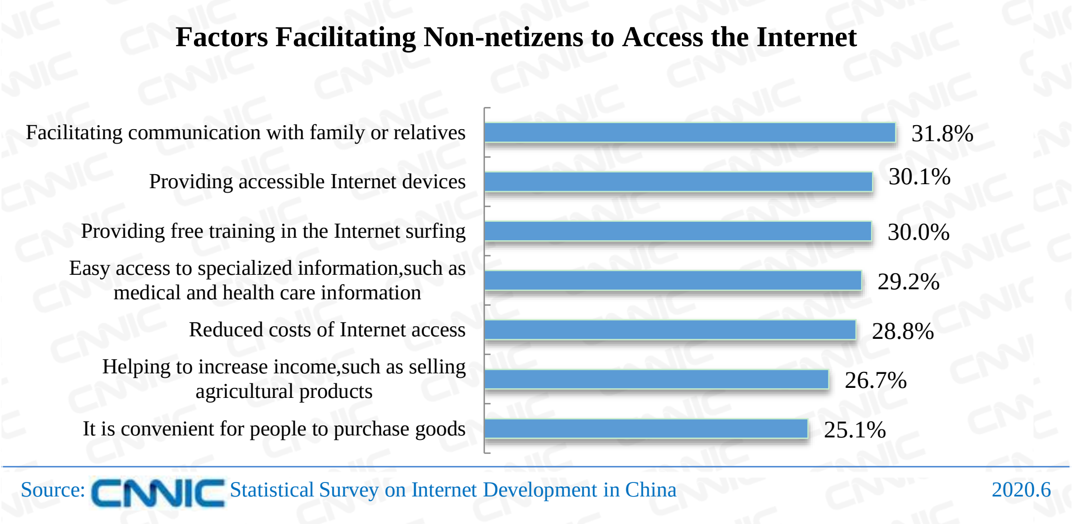

Figure 23 Factors Facilitating Non-netizens to Access the Internet

II. The Attribute Structure of Internet Users

(I) Gender Structure

As of June 2020, the ratio of male to female among Chinese netizens is 51.0:49.0, which is roughly

the same as that in China's overall population (51.1:48.9)[17].

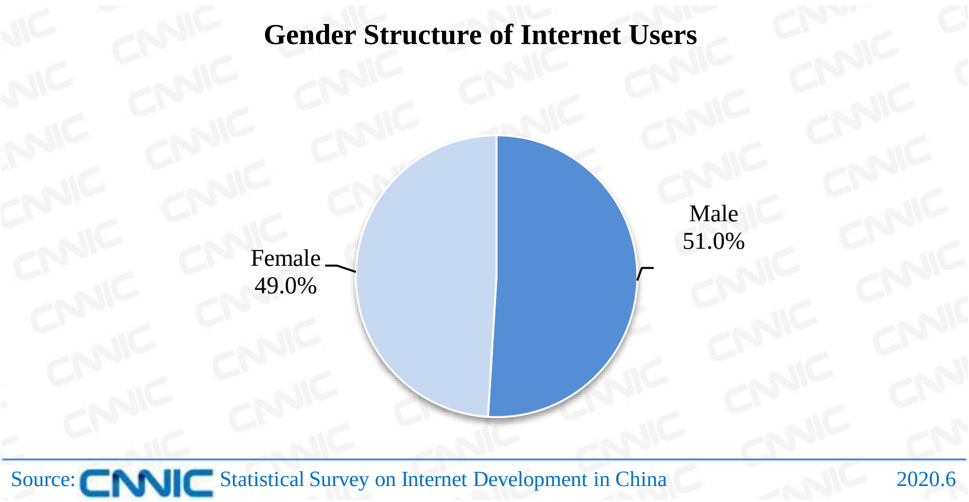

Figure 24 Gender Structure of Internet Users

(II) Age Structure

As of June 2020, the proportion of Internet users aged 20-29 and 30-39 was 19.9% and 20.4% respectively, higher than that of other age groups; that of Internet users aged 40-49 was 18.7%; and that of Internet users aged 50 and above increased from 16.9% in March 2020 to 22.8% in June 2020, with the Internet further penetrating middle- and old-age groups.

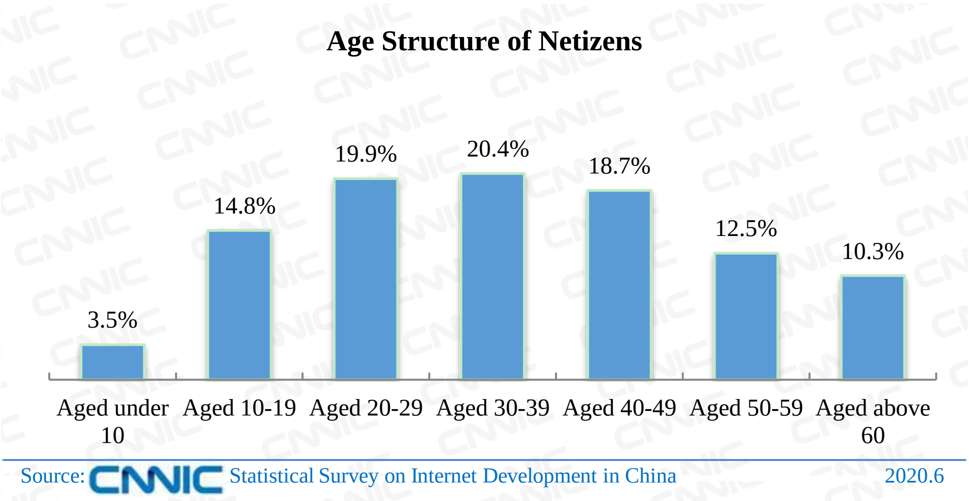

Figure 25 Age Structure of Netizens

(III) Educational Background

[17] Source: the *Statistical Communique of the People's Republic of China on the 2019 National Economic and Social Development* released by the National Bureau of Statistics of China.

As of June 2020, the proportions of netizens graduating from junior middle schools as well as senior middle schools, vocational schools or technical schools were 40.5% and 21.5%, respectively. The proportion of Internet users holding a diploma from colleges or above was 18.8%, respectively.

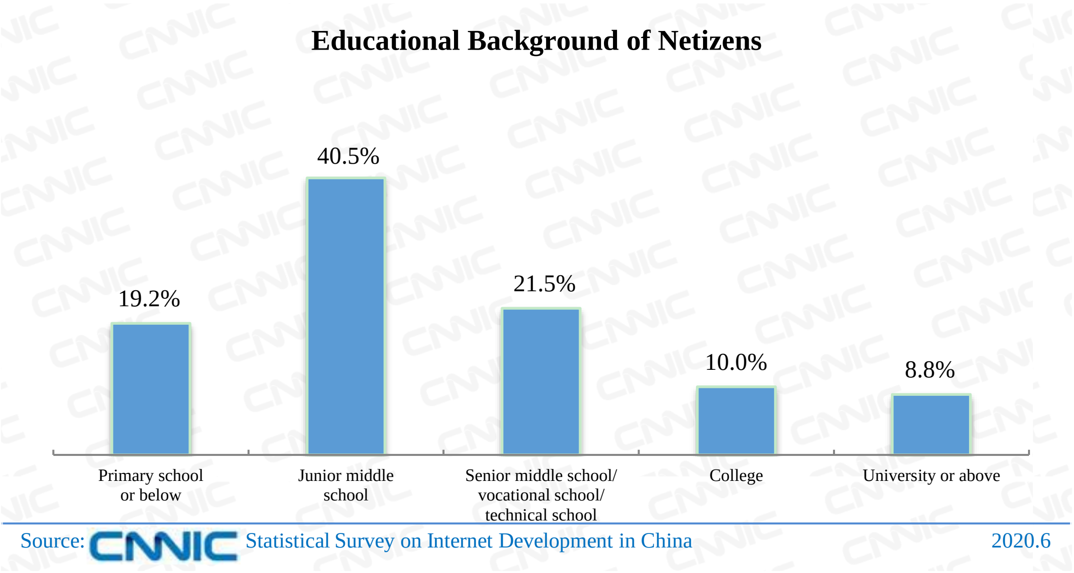

Figure 26 Educational Background of Netizens

(IV) Occupational Structure

As of June 2020, students got the largest share of Internet users in China, accounting for 23.7%, followed by the self-employed/freelance workers, making up 17.4%, and the agricultural, forestry, animal husbandry and fishery workers, representing 15.3%.

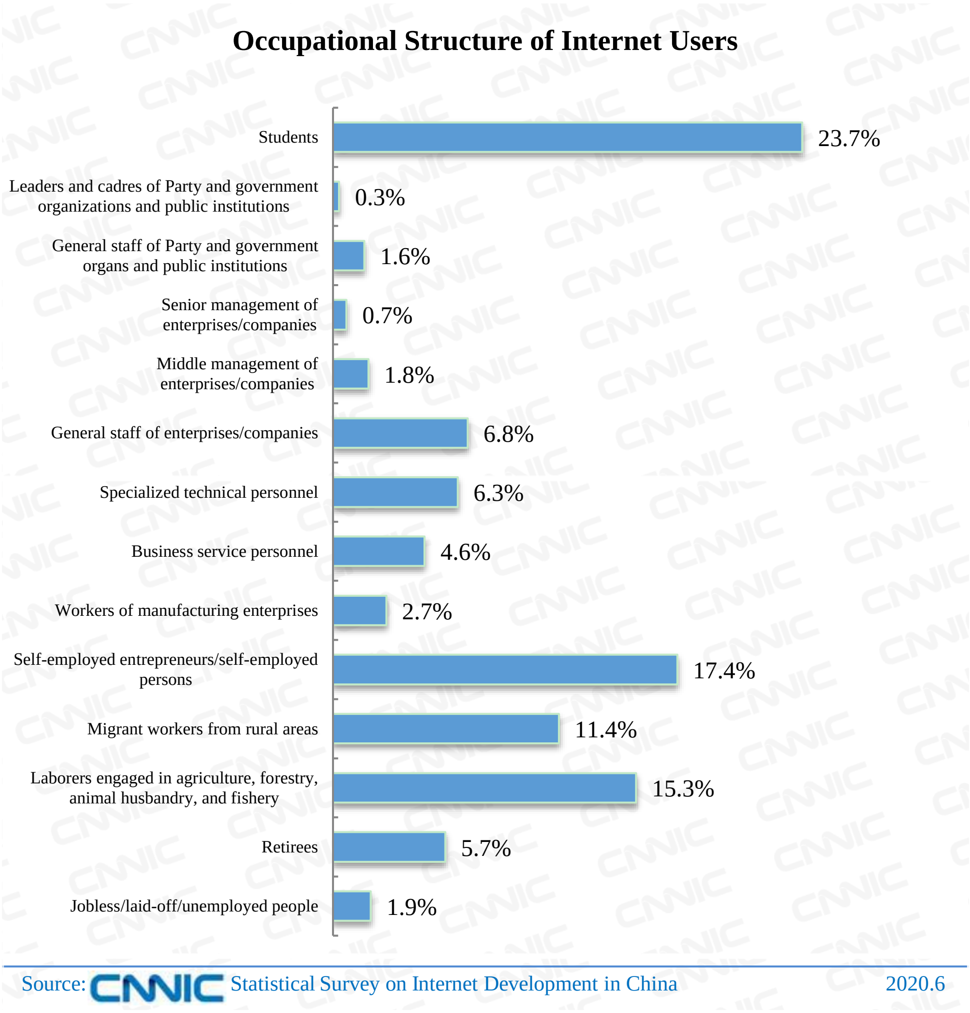

Figure 27 Occupational Structure of Internet Users

(V) Income Structure

As of June 2020, the proportion of Internet users with monthly income[18] from 2,001 to 5,000 yuan was 32.6%; that of those users with monthly income above 5,000 yuan was 24.2%; and that of those netizens with monthly income of 1,000 yuan or less was 21.0%.

[18] Monthly income: the income of students includes living allowances provided by families, salary earned from work-study programs, scholarships and others. The income of workers engaged in agriculture, forestry, animal husbandry and fishery includes the living allowances provided by children, income of agricultural production, and government subsidy. The income of those who are jobless, laid off or unemployed includes the living allowances provided by children, government relief and subsidy, pension, and subsistence allowances. The income of retirees includes the living allowances provided by children and pension.

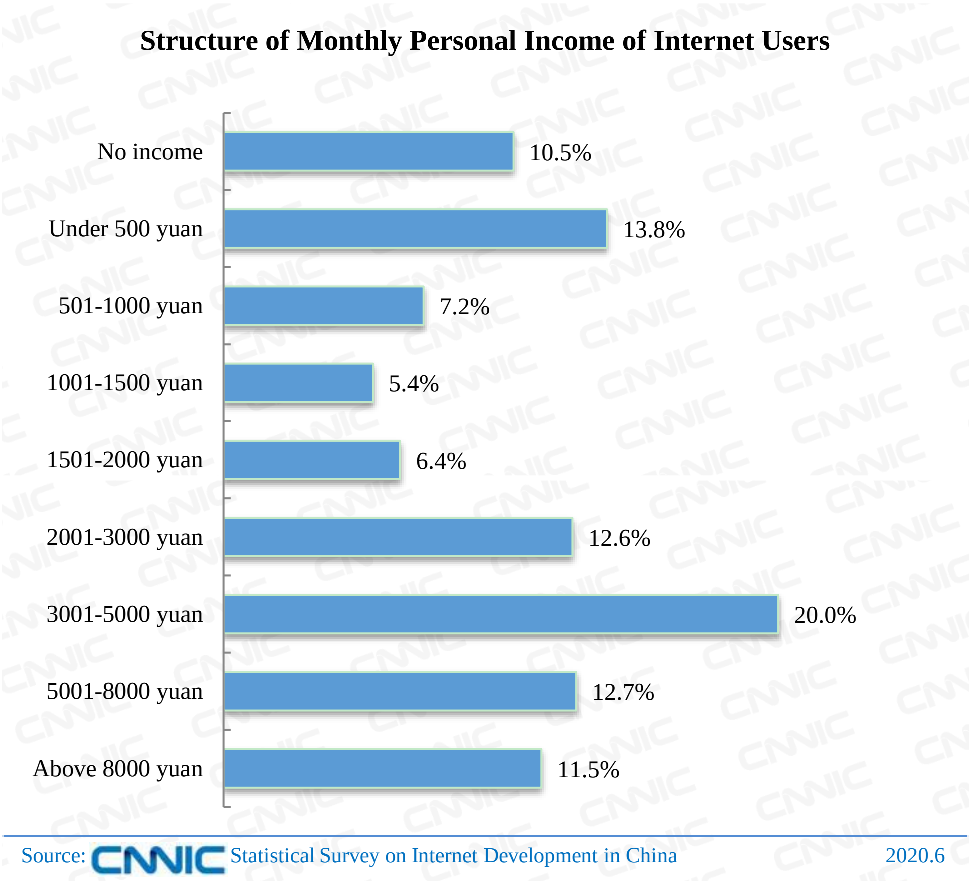

Figure 28 Structure of Monthly Personal Income of Internet Users

Chapter Three The Development of Internet Applications

I. Overview of Internet Applications

In the first half of 2020, China saw steady growth in personal Internet applications. The most significant growth in user size was found in live streaming e-commerce, video clip and online shopping applications, with growth rates of 16.7%, 5.8% and 5.5% respectively. A steady growth rate ranging from 1% to 5% was seen in instant messaging, search engine and other basic applications as well as online games, online video, cell phone literature and other online entertainment applications. In terms of mobile Internet applications, the growth rate of mobile shopping users exceeded 5%.

Table 3 User Size and Utilization Rate of Internet Applications of Internet Users from Mar. 2020 to Jun. 2020

	Jun. 2020		Mar. 2020		
Applications	Number of Internet users (10,000)	The percentage of Internet users using the application	Number of Internet users (10,000)	The percentage of Internet users using the application	Growth rate
Instant messaging	93079	99.0%	89613	99.2%	3.9%
Search engine	76554	81.5%	75015	83.0%	2.1%
Online news	72507	77.1%	73072	80.9%	-0.8%
Telecommuting	19908	21.2%	-	-	-
Online shopping	74939	79.7%	71027	78.6%	5.5%
Online meal ordering	40903	43.5%	39780	44.0%	2.8%
Online payment	80500	85.7%	76798	85.0%	4.8%
Internet wealth management	14938	15.9%	16356	18.1%	-8.7%
Online games	53987	57.4%	53182	58.9%	1.5%
Online video (including video clip)	88821	94.5%	85044	94.1%	4.4%

	Jun. 2020		Mar. 2020		
Applications	Number of Internet users (10,000)	The percentage of Internet users using the application	Number of Internet users (10,000)	The percentage of Internet users using the application	Growth rate
Video clip	81786	87.0%	77325	85.6%	5.8%
Online music	63855	67.9%	63513	70.3%	0.5%
Online literature	46704	49.7%	45538	50.4%	2.6%
Live streaming[19]	56230	59.8%	55982	62.0%	0.4%
Online Car-hailing Services	34011	36.2%	36230	40.1%	-6.1%
Online education	38060	40.5%	42296	46.8%	-10.0%
Online medical services	27602	29.4%	-	-	-

Table 4 User Size and Utilization Rate of Applications of Mobile Internet Users from Mar. 2020 to Jun. 2020

	Jun. 2020		Mar. 2020		
Applications	Number of Internet users (10,000)	Utilization ratio of mobile Internet users	Number of Internet users (10,000)	Utilization ratio of mobile Internet users	Growth rate
Mobile instant messaging	93037	99.8%	89012	99.2%	4.5%
Mobile Search Engine	76078	81.6%	74535	83.1%	2.1%
Mobile news	71999	77.2%	72642	81.0%	-0.9%
Mobile shopping	74696	80.1%	70749	78.9%	5.6%
Mobile meal ordering	40720	43.7%	39653	44.2%	2.7%
Mobile payment	80172	86.0%	76508	85.3%	4.8%
Mobile game	53592	57.5%	52893	59.0%	1.3%
Mobile music	63598	68.2%	63274	70.5%	0.5%

[19]Live streaming includes live-stream e-commerce, live sport broadcasting, host live show, live game streaming, and live concert streaming.

Applications	Jun. 2020		Mar. 2020		Growth rate
	Number of Internet users (10,000)	Utilization ratio of mobile Internet users	Number of Internet users (10,000)	Utilization ratio of mobile Internet users	
Cell phone literature	46515	49.9%	45255	50.5%	2.8%
Mobile learning	37668	40.4%	42023	46.9%	-10.4%

In June 2020, the per capita number of mobile phone Apps[20] among netizens aged 15-19 was 83, the largest proportion. In the second place, Internet users aged 20-29 had 72 mobile phone Apps on average. The per capita number of mobile phone Apps among netizens aged 15 or above gradually decreased with the increase of age. Netizens aged 60 or above installed 44 mobile phone Apps on average.

Per Capita Number of Mobile Phone Apps by Netizens' Age

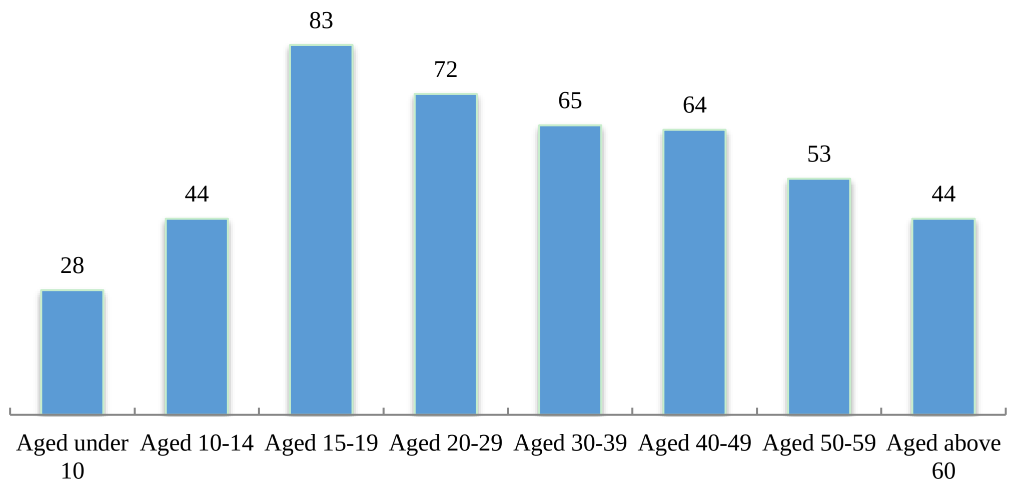

Source: China Telecom 2020.6

Figure 29 Per Capita Number of Mobile Phone Apps by Netizens' Age

II. Basic Apps

(I) Instant messaging

Up to June 2020, the user size of instant messaging was 930.79 million or 99.0% of China's total netizen population, up 34.66 million over March 2020; the number of mobile instant messaging

[20] Per capita number of mobile phone Apps refers to that of Apps installed on mobile phones of netizens on average.

users had reached 930 million, up 40.24 million from March 2020, making up 99.8% of mobile Internet users.

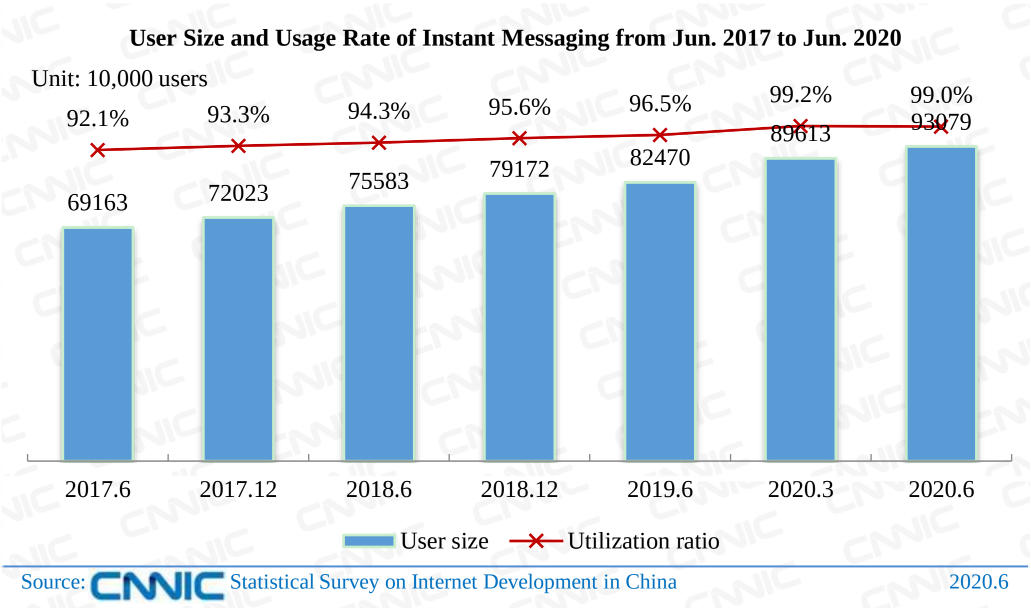

Figure 30 User Size and Usage Rate of Instant Messaging from Jun. 2017 to Jun. 2020

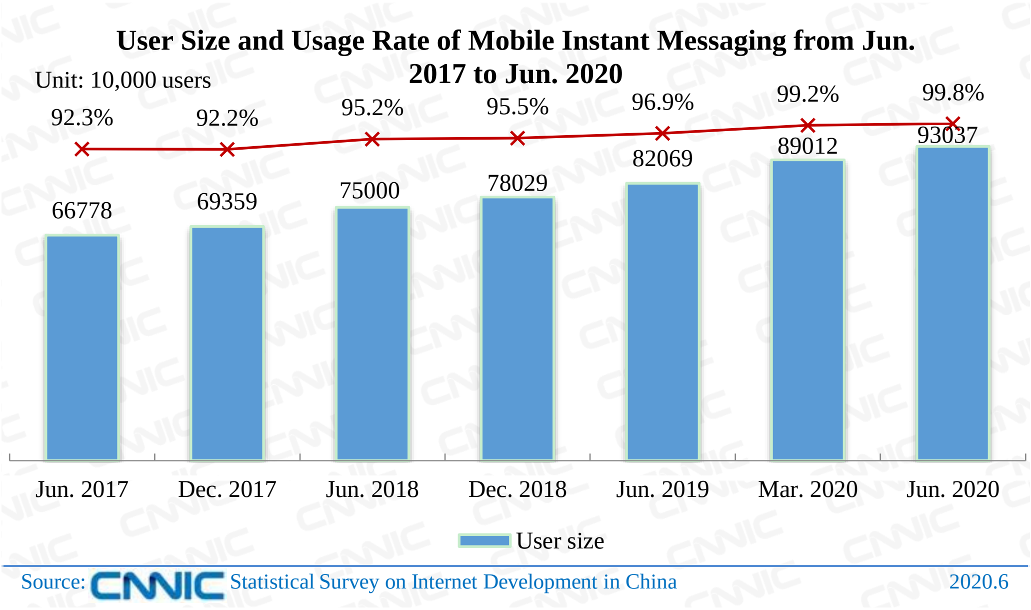

Figure 31 User Size and Usage Rate of Mobile Instant Messaging from Jun. 2017 to Jun. 2020

In the first half of 2020, the instant messaging industry grew at a fast pace amid the COVID-19 epidemic, which is embodied in the following three aspects.

Regarding the industry development, instant messaging became one of the fastest growing Internet applications during the COVID-19 epidemic. In the personal instant messaging, communication activities among netizens have gradually shifted from offline to online as a result of the COVID-19 epidemic. In this context, instant messaging, a primary online communication

channel, has further enhanced user activity. According to relevant data[21], both WeChat and QQ achieved double-digit year-over-year growth in total daily messages and time length in the first quarter. Regarding instant messaging applications for enterprises, the strong demand for a contactless office environment has prompted a growing number of enterprises to use instant messaging products amid the COVID-19 outbreak, driving significant growth in the user size of this market. Based on relevant data, over 300 million users used DingTalk during the epidemic, helping 15 million enterprises resume work and production[22]; enterprise-version WeChat users grew from 60 million to 250 million[23] during the period.

With respect to market competition, the number of new entrants in the instant messaging continues to increase, further intensifying the competition in the industry. Huawei's WeLink, ByteDance's Feishu, Pinduoduo's Knock and other enterprise-class instant messaging products were available to the public in the first half of 2020. The enterprise-class instant messaging services by these tech companies marked the evolution of the competition from a dual-pillar to a multi-polar landscape. In addition, the three major telecom carriers jointly released the *White Paper on 5G Messaging* to offer rich-media instant messaging services[24] directly to subscribers from the perspective of a telecom operator. As an upgraded version of short message service (SMS), 5G messaging has featured a large user base and low operating costs compared to existing instant messaging products. However, there is a gap between 5G messaging and other mature instant messaging products in user experience and service ecosystem.

In terms of social impact, instant messaging serves as an important information platform for anti-epidemic efforts. First, official accounts and applets of instant messaging applications have become an important channel for releasing epidemic information. According to relevant data[25], the COVID-19 modules of instant messaging applets delivered over 6 billion epidemic updates to users nationwide in the first quarter alone. Second, instant messaging companies contributed to reopening businesses and schools across China. Based on big data, facial recognition and satellite positioning, instant messaging enterprises have introduced health QR code, ride QR code and school resumption code to digitally assist the society in resuming its normal functioning. Third, instant messaging companies further facilitated the IT-based remodeling of hospitals during the epidemic. For example, some instant messaging companies have integrated their instant messaging, cloud services and online conferencing into "digital solutions for hospitals fighting the epidemic," providing a convenient and efficient mobile office environment for frontline medical staff in their training, consultations, and updating of patient information.

(II) Search engine

As of June 2020, the user size of search engine was 765.54 million or 81.5% of China's total netizen population, up 15.39 million over March 2020; the number of mobile search engine users had reached 761 million, up 15.42 million from March 2020, accounting for 81.6% of mobile Internet users.

---

[21]Source: Tencent's financial report 2020 Q1.
[22]Source: the 2020 DingTalk Spring/Summer Launch.
[23] Source: Sina https://tech.sina.com.cn/roll/2020-05-18/doc-iirczymk2296915.shtml, May 18, 2020.
[24] Rich-media instant messaging services refers to a instant messaging service that supports a variety of message formats such as text, voice, image, video and location.
[25] Source: Tencent's official website, https://www.tencent.com/zh-cn/articles/2201019.html, March 29, 2020.

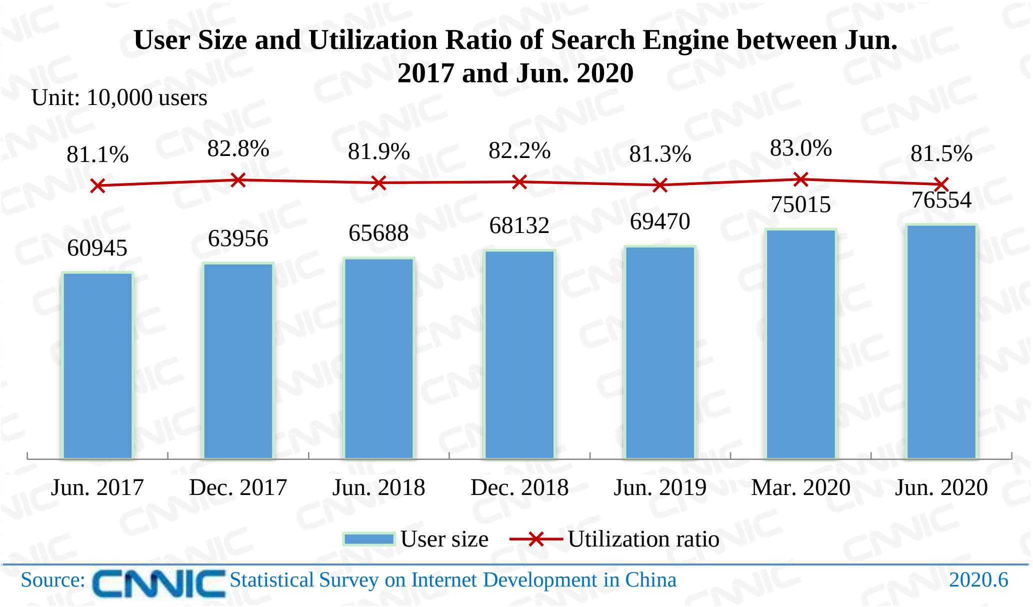

Figure 32 User Size and Utilization Ratio of Search Engine between Jun. 2017 and Jun. 2020

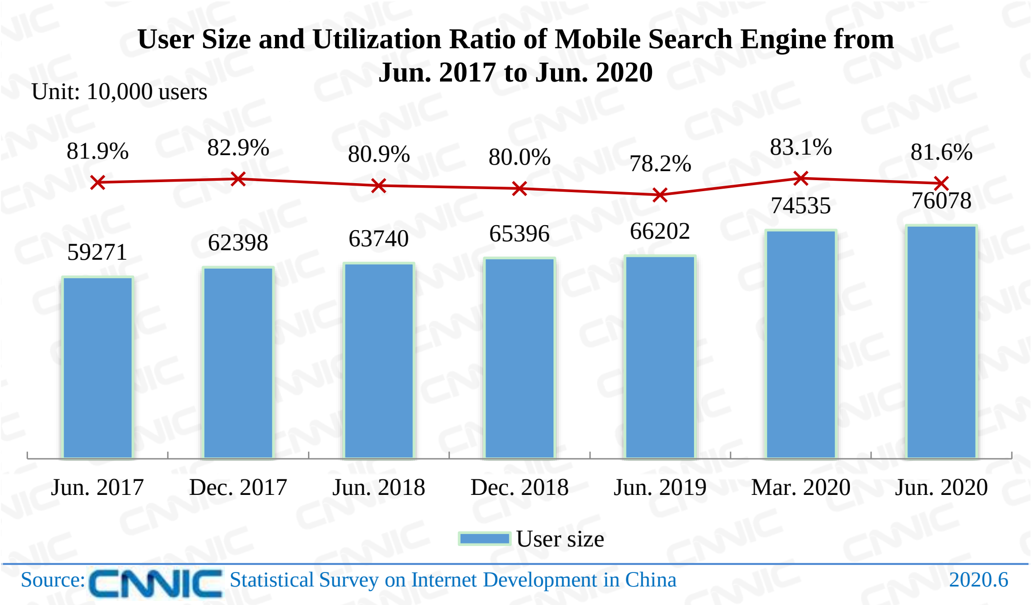

Figure 33 User Size and Utilization Ratio of Mobile Search Engine from Jun. 2017 to Jun. 2020

In the first half of 2020, search engines continuously advanced the content ecosystem and made services more intelligent in a bid to broaden revenue channels. Also, the efficiency and effectiveness of social governance for massive information searches were initially demonstrated amid the COVID-19 epidemic.

Search engines accelerate the development of the content ecosystem. First, maximizing the commercial value of traffic is the main driver for building a content ecosystem. To enhance profitability, search engine companies have established the content ecosystem to retain users,

promote traffic monetization[26] and reduce cost of revenue. According to the data[27], Baidu saw its steady traffic growth from in-app search engine and mobile ecosystem, with the cost of revenue declining for two consecutive quarters in 2020. **Second, enriching original content and purchasing content are the primary means to build a content ecosystem.** Through two years of vigorous development, the number of Baidu Baijiahao creators increased by 52% in the second quarter compared to the same period last year.[28] They are now main providers of original content for the platform's content ecosystem. Baidu Baijiahao is developing itself as part of an information search and distribution system together with news feeds, encyclopedias, videos, and live streaming. After launching its independent App, Toutiao Search acquired baike.com and baikemy.com by integrating products and contents under ByteDance. The search platform has further enhanced the comprehensiveness and professionalism of its search contents, so as to meet the multiple demands of users for contents.

Search engines have been made more intelligent continuously. First, more smart search portals are being developed. Smart assistants, changing the way information is searched, are increasingly accepted by users. According to the data[29], the number of voice interaction via DuerOS totaled 5.8 billion in June 2020, up 57% year-on-year. Alibaba's Quark has expanded its search portal through AI tools, with camera-based search increasing by more than 10 times over the last year[30]. **Secondly, search-based direct access services[31] are more intelligent**. For example, WeChat's Search is becoming smarter in its search-based services that can be directly linked to corresponding information, entertainment resources and mini programs based on users' keywords, providing them with more efficient ways to obtain content, brands and services. For example, a "nucleic acid test" can be registered directly and a brand name can be linked to its purchase page directly, when users search such keywords.

Search engines begin to play their role in social governance. The significantly enhancement of search user activity was due to the COVID-19 outbreak. During the outbreak, Baidu's search traffic grew by over 30%,[32] while Sogou's search and information flow products were accessed for more than 560 million[33]. Search engines amass a tremendous amount of information on users' needs and serve as important entrances and guides in fighting against the epidemic and reopening businesses and schools. They provide a vital reference for tracking public opinion, squashing rumors through scientific knowledge and guiding the distribution of anti-epidemic materials. In the future, the in-depth mining, modeling and prediction of search data play an essential role in researching emergencies, serving the people and promoting economic recovery, thus helping to build a digital governance system and improve governance capabilities.

---

[26] Traffic monetization refers to the process of converting website traffic into cash income through commercial means.
[27] Source: Baidu's financial reports in Q1 and Q2 2020.
[28] Source: Baidu's financial report in Q2 2020.
[29] Source: Baidu's financial report in Q2 2020.
[30] Source: sohu.com.
https://www.sohu.com/a/412567330_162522?_f=index_pagefocus_6&_trans_=000014_bdss_dkgyxqsP3p:CP=, August 11, 2020.
[31] Search-based direct access service means that a search engine matches the keyword a user enters, automatically loads applet components, and directly displays function links of related services in the search results.
[32] Source: Teleconference for Baidu's financial report 2019 Q4.
[33] Source: Sogou's financial report 2019 Q4.

(III) Online news

As of June 2020, the number of online news users in China was 725.07 million or 77.1 percent of the overall Internet users, down 5.65 million from March 2020; the number of mobile news users amounted to 720 million, down 6.42 million from March 2020, making up 77.2 percent of mobile Internet users.

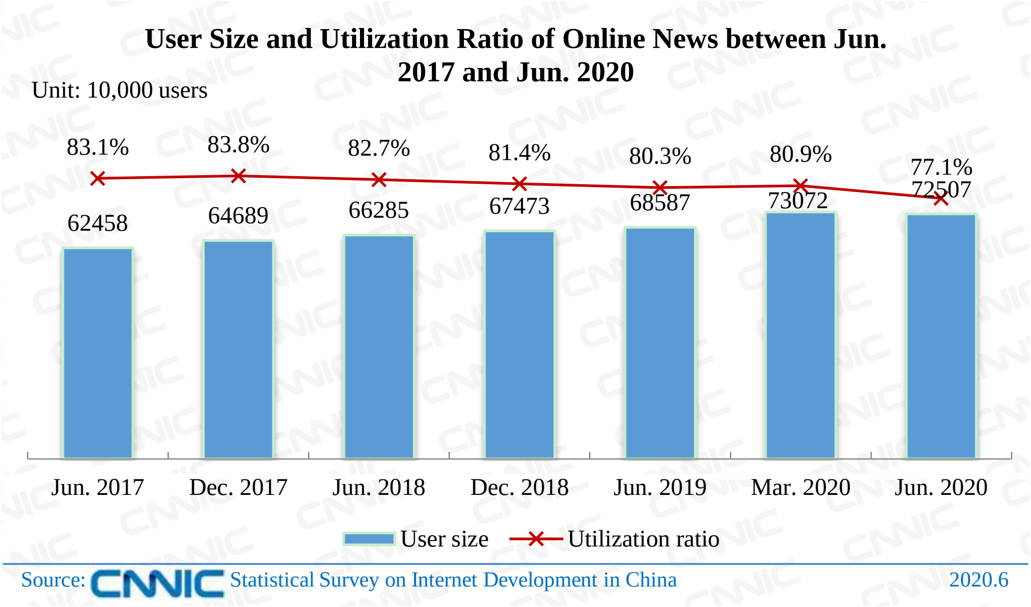

Figure 34 User Size and Utilization Ratio of Online News between Jun. 2017 and Jun. 2020

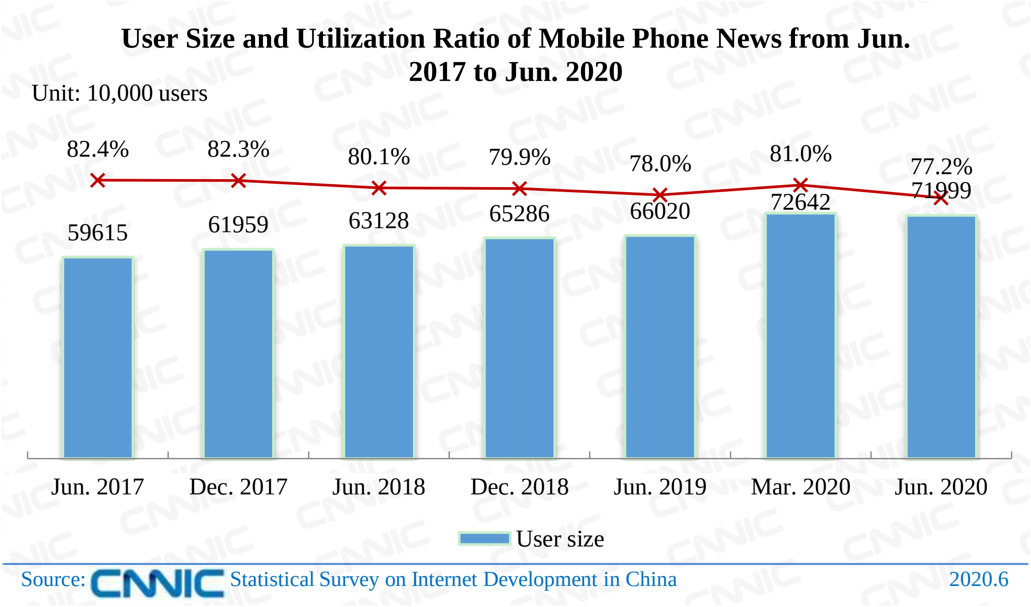

Figure 35 User Size and Utilization Ratio of Mobile Phone News from Jun. 2017 to Jun. 2020

In the first half of 2020, the online news industry has covered the unexpected epidemic in detail and assisted in responding to the emergency in a variety of ways. By fully applying various types of platforms, online news media continued to make news dissemination more accessible and

effective. **Online news media were assisting in the fight against the epidemic in diverse ways. The first was to enrich the forms of presentation and bolster the confidence in fighting the epidemic.** In the anti-epidemic frontline of Wuhan, Hubei province, online news media produced Vlogs, posters and publicity videos to let the people of the country have a thorough understanding of the real situation, bolstering their confidence and determination to win the battle against the COVID-19 epidemic. **The second was to publicize China's achievements in its anti-epidemic efforts.** In April 2020, Xinhua News Agency released a video entitled *Once Upon A Virus* overseas, receiving widespread attention and garnering more than one million views and tens of thousands of likes and retweets[34]. During the epidemic, China Global Television Network (CGTN) broadcast the documentary series *24 Hours in Wuhan*, which had a video reach of over 14 million and over 6 million views as of May 2020[35]. Online news media voiced their opinions, enhancing the understanding and support of the world's other parts for China's fight against the epidemic.

Online news media have further expanded the information reach and improved the effects of news dissemination. Amid the epidemic in 2020, online news media released news and information through social networking platforms, news and information Apps, search engines, and video clip platforms and made it easier for users to access news and information and distribute them multiple times, so as to achieve the better effects of news dissemination. The above-mentioned measures helped users to obtain the latest developments in the fight against the epidemic in a more timely manner, do a good job of personal protection, and pool their strengths to contain the epidemic, in a bid to avoid the further spread of the epidemic. Specifically, CCTV News tracked the latest data on the epidemic around the world and released anti-epidemic information through Weibo, Bilibili and other platforms, with daily readings exceeding one million. Xiakedao, the WeChat official account of the overseas edition of the People's Daily, tracked the epidemic at home and abroad and pushed epidemic-related analysis articles timely, with readings of many articles surpassing 100,000. Sina News tracked and visualized the latest data on the epidemic in China and beyond, providing a convenient way for Chinese across the world to learn about the epidemic.

(IV) Social networking applications

As of June 2020, the use rate of WeChat Moments was 85.0%, basically unchanged from March 2020; the use rates of Qzone and Weibo were 41.6% and 40.4% respectively, down 6 percentage points and 2.1 percentage points respectively over March 2020.

[34] Source: sina.com.cn, http://k.sina.com.cn/article_3215229040_bfa4807000100rj25.html, May 5, 2020.
[35] Source: sina.com.cn, https://k.sina.com.cn/article_2759348142_a4784fae01900rrye.html?cre=tianyi&mod=pcpager_focus&loc=38&r=9&rfunc=100 amp;tj=none&tr=9, May 19, 2020.

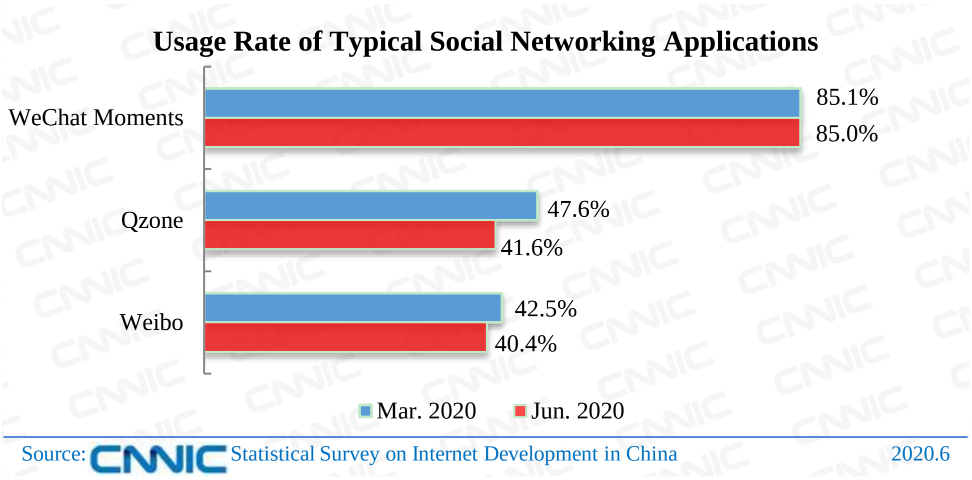

Figure 36 Usage Rate of Typical Social Networking Applications

In the first half of 2020, the market landscape of social networking applications was stable, with products being innovated. During the epidemic, social networking platforms played an important role in international communication.

The overall market landscape of social networking applications is stable with continuous exploration and innovation conducted in segments. WeChat Moments, Weibo and other mainstream social networking platforms have long accounted for most of the traffic. They are developing a refined system featuring closed-loop traffic and service ecosystem through the continuous enrichment of video clips, e-commerce, local life and other services. Faced with a limited market space, some social networking applications have tapped into the unique needs of niche groups as a foothold for innovation. To meet the personalized needs of Generation Z[36], some social networking companies are constantly introducing new social products for specific segments, exploring the opportunities brought about by new technologies and generational changes of users, and striving for innovative breakthroughs. In 2019, 50-odd social networking applications were unveiled in succession.[37] In the first half of 2020, a number of newly segmented applications were also launched, such as Alibaba's real social product "Real as Me."

During the epidemic, social networking platforms played an important role in the dissemination of information in China and beyond. First, Chinese netizens, through domestic social networking platforms, kept abreast of the development of the COVID-19 epidemic abroad. Overseas bloggers from multiple countries and regions share their local epidemic situations on their Weibo homepages in the forms of picture, text and video. Weibo has become an important window for Chinese people to understand the trend of overseas epidemics. Up to March 2020, 1.92 million videos concerning the epidemic had been uploaded by overseas Weibo users, covering 36 countries across five continents, with over 75.8 billion views[38]. **Secondly, Chinese netizens and enterprises proactively showcase China's image via overseas social platforms.** Videos and news reflecting China's anti-epidemic efforts have been widely disseminated on overseas social media, helping overseas netizens understand the real situation in this regard. *Wuhan, Stay Strong. We're Waiting for You*, a homemade short film by Chinese netizens, was translated into

[36] Generation Z refers to the group born in 1995 and later.
[37] Source: iiMedia Research.
[38] Source: Weibo.

10 languages and disseminated on overseas social platforms to share China's experience and tell China's stories. Chinese enterprises operating overseas publicized anti-epidemic aid on Facebook, Twitter and other social platforms to expand the influence of Chinese brands.

(V) Telecommuting Apps

As of June 2020, the number of telecommuting users in China amounted to 199 million or 21.2% of overall Internet users. In the first half of 2020, telecommuting Apps served as an important Internet tool for ongoing anti-epidemic efforts and normal socio-economic functioning. Telecommuting Apps have integrated offline and online business of enterprises by reshaping their original working models. They are also expected to become a normal operation tool after the epidemic is over. They are also an important means to promote the digital transformation of enterprises.

The demand of telecommuting App users is soaring, and the market size is growing rapidly. First, in terms of user size, telecommuting Apps were a widely used model adopted by enterprises and individual users amid the epidemic. Especially in the economic reopening period, the number of users and duration have grown exponentially. According to the data, on February 4, 2020, 60,000 new users held their meetings via Tianyi Cloud Conference, with a total of 90,000 meeting hours.[39] From June to July 2020, the average daily use of teleconferencing was 110 minutes[40], making the cloud conference a routine App. **Secondly, regarding the market size**, more than 18 million enterprises in China have adopted the telecommuting model during the 2020 Spring Festival. The market size of smart mobile office is projected to reach 37.5 billion yuan in 2020, with a growth rate of 30.2%[41].

The telecommuting application market is fiercely competitive, and the service capabilities are being increased. First, market players are more diversified. Driven by the demand, all types of companies are rapidly competing to capture the market. Large Internet enterprises relying on existing products expand their presence across the board, such as Tencent, Alibaba and Baidu. Professional software providers focusing on vertical industries refine their lean services, such as Vidyo in health care, and XYLink in education and health care. software and hardware integration suppliers integrated a variety of business processes into a unified platform to achieve the combination of a range of office software and hardware, like Huawei Cloud WeLink. **Secondly, software performance and function have been upgraded constantly.** As the competition is heating up, telecommuting applications have been improved rapidly in their service capabilities. In terms of software performance, the number of concurrent users, video definition, low latency and other performances have been upgraded constantly to optimize user experience. For example, DingTalk and enterprise-version WeChat support 300 users concurrently. Regarding software functions, the innovative integration of Internet media technology with traditional OA software functions and the powerful telecommuting service capabilities help enterprises maintain normal operation during the epidemic. For instance, the combination of live streaming and online document

[39]Source: China Telecom Tianyi Cloud.
[40]Source: the data collected by the Computer Network Information Center under the Chinese Academy of Sciences cover 299,727 Internet devices in 12 cities of 10 provinces, with monitoring software including Zoom, VooV Meeting and DingTalk.
[41]Source: iiMedia Research.

collaboration tools improves the communication efficiency of teleconferencing.

Telecommuting makes a big push for the development of infrastructure and the expansion of the software and hardware industries. First, telecommuting has advanced the development of IT-based infrastructure. In order to meet the needs of telecommuting for video definition, information latency, server concurrent processing and other core performances, a variety of IT-based infrastructures continue to accelerate, including high-speed wireless network, expansion of cloud computing services, deployment of high-performance servers, and R&D and production of high-capacity memory chips. During the COVID-19 outbreak, VooV Meeting, one video conferencing tool released by Tencent, expanded its capacity by over 100,000 cloud hosts with one million cores of computing resources[42] in eight days, giving a strong boost to the growth of the server market. **Secondly, related intelligent software and hardware industries have been developed rapidly.** The growing integration of telecommuting and enterprise-based digital management has significantly increased the demand for related intelligent hardware, such as intelligent access control, smart router, intelligent interactive screen, and remote video device. Telecommuting services provide one-stop solutions through the integration of software and hardware to accelerate the innovations for enterprise services market. According to the data,[43] a fast-growing number of cloud-based services were launched for enterprises. In the first quarter of 2020, China's SDS[44] market size grew at a rate of 25.9% year-on-year.

[42] Source: sina.com.cn,https://tech.sina.com.cn/i/2020-02-06/doc-iimxyqvz0776909.shtml, February 6, 2020.
[43]Source: IDC's *China Software Defined Storage (SDS) Market Tracking Report Q1 2020*.
[44] SDS refers to Software Defined Storage.

III. Business Transaction Applications

(I) Online shopping

Up to June 2020, the user size of online shopping was 749.39 million or 79.7% of China's total netizen population, up 39.12 million over March 2020; the number of mobile shopping users had amounted to 747 million, up 39.47 million from March 2020, taking up 80.1% of mobile Internet users.

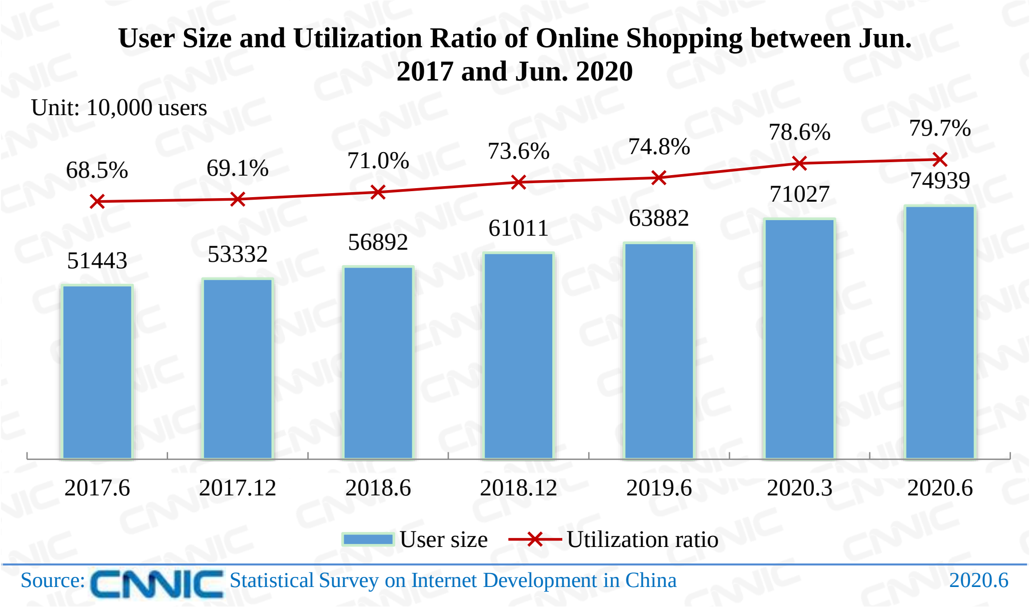

Figure 37 User Size and Utilization Ratio of Online Shopping between Jun. 2017 and Jun. 2020

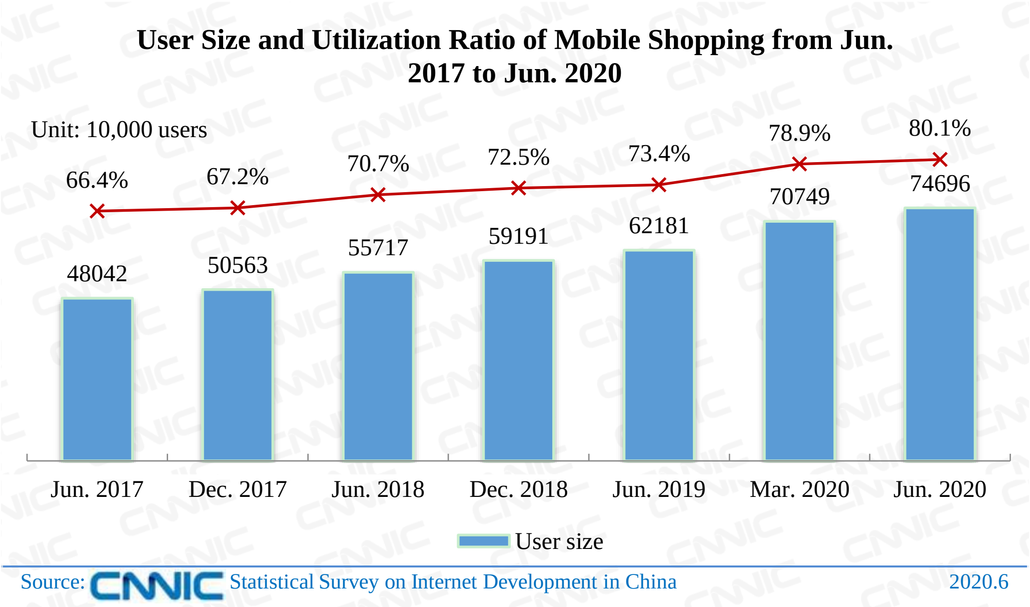

Figure 38 User Size and Utilization Ratio of Mobile Shopping from Jun. 2017 to Jun. 2020

Since 2013, China has been the world's largest online retail market for seven consecutive years. Despite the severe challenge posed by the COVID-19 epidemic, the ability of online retail market sustaining itself was further demonstrated. Online retail sales reached 5.15 trillion yuan in the first half of the year, a YoY increase of 7.3%. Specifically, online sales of physical goods rose 14.3% year-on-year to nearly 4.35 trillion yuan, accounting for 25.2% of total retail sales, 25.7 percentage points higher than the YoY growth rate of total retail sales of consumer goods.[45] Online retail has given a strong boost to opening up China's economic circulation by expanding domestic demand through consumption, bolstering development through innovation, and energizing the market.

While expanding domestic demand, online consumption has made the economy more resilient. The first was to ensure people's livelihood amid the epidemic. The "stay-at-home economy[46]" emerged in the first half of the year, with commodity consumption moving toward online platforms significantly. Major e-commerce platforms initiated their responses by giving full play to their supply-chain strengths to ensure the supply of necessities through direct overseas procurement and via domestic brand merchants. **The second was to give a strong boost to the post-epidemic recovery in consumption.** E-commerce platforms and local governments jointly issued various forms of e-consumption coupons to activate online and offline consumption by subsidizing users. The Ministry of Commerce of China and other departments organized e-commerce platforms to launch the Online Shopping Festival for Quality Brand Products, driving the national online retail sales to exceed 430 billion yuan[47] during the same period. In the large-scale "June 18" e-commerce promotion, Tmall and JD.com reached 698.2 billion yuan and 269.2 billion yuan[48] respectively, demonstrating the power of the domestic demand engine and the momentum of economic transformation. All these efforts unleashed the potential of consumption and enhanced economic resilience.

Apart from boosting the development, new forms and models of business have also helped upgrade the economy. First, cross-border e-commerce has become an important way to stabilize foreign trade and promote the consumption return. From January to May 2020, the total import and export retail commodities through the customs-based cross-border e-commerce management platform amounted to 71.73 billion yuan, up 22.4% year-on-year[49]. Foreign trade enterprises realized survival and development through online marketing and trading. During the 127th Canton Fair, nearly 26,000 domestic and overseas exhibitors achieved global sourcing via "cloud" platforms[50]. Also, cross-border e-commerce further promoted the consumption return. As of June 2020, the user size of cross-border e-commerce[51] in China had reached 138 million. **Second, new models such as fresh-food e-commerce are driving all types of consumption growth.** Due to the epidemic in the first half of the year, livelihood consumer applications such as fresh-food e-commerce and online grocery shopping witnessed explosive growth. As of June 2020, the user size amounted to 257 million, making up 27.4% of overall Internet users. In addition, the sizes of

---

[45] Source: the National Bureau of Statistics of the People's Republic of China, http://www.stats.gov.cn/tjsj/zxfb/202007/t20200716_1776198.html, July 16, 2020.

[46] Stay-at-home economy refers to a new form of economy that emerges with the rise of the Internet, covering teleworking, and at-home shopping and online meal ordering.

[47] Source: the Ministry of Commerce of the People's Republic of China http://www.mofcom.gov.cn/article/i/jyjl/l/202006/20200602973242.shtml,June 12, 2020.

[48] Source: Sina Finance, http://finance.sina.com.cn/stock/relnews/hk/2020-06-19/doc-iircuyvi9289301.shtml, June 19, 2020.

[49] Source: the General Administration of Customs of the People's Republic of China (GACC).

[50] Source: chinanews.com http://www.chinanews.com/cj/2020/06-29/9224175.shtml, June 29, 2020.

[51] Cross-border e-commerce users refer to those who purchased imported goods online in the last six months.

agricultural products e-commerce and second-hand e-commerce users[52] reached 248 million and 61.43 million respectively, playing an important role in promoting the uplift of agricultural products and the development of the idle economy.

E-commerce empowers the majority of small and medium-sized enterprises to meet the demand of the market. First, the online transformation heightened the capabilities of businesses in their operations amid the epidemic. During the epidemic prevention and control period, "online economy," "stay-at-home economy" and other new forms had been derived from online retail, offering opportunities for traditional enterprises and businesses to digitally transform and upgrade themselves. Through livestreamed e-commerce and WeChat-based membership group and other digital methods, small- and medium-sized enterprises (SMEs) sped up their online operations, promoting the resumption of work and production. **Secondly, e-commerce platforms are empowered to relieve the difficulties of small- and medium-sized enterprises.** In the first half of 2020, multiple e-commerce platforms intensified their efforts to support SMEs' development by empowering their technologies and resources. For example, Jingdong Digital Science and Technology provides small and medium-sized enterprises with solutions for blockchain electronic contracts, blockchain trade secret protection and data services based on Jingdong's intelligent supply chain. Alibaba launched the "Spring Thunder Plan" to support small and medium-sized enterprises, and through the activation of the digital industry belt and other important initiatives, use a reverse customization model to help factories sell directly online.

(II) Online meal ordering

Up to June 2020, the user size of online meal ordering was 409.03 million or 43.5% of China's total netizen population, up 11.24 million over March 2020; the number of mobile meal ordering had reached 407.20 million, up 10.67 million from March 2020, accounting for 43.7% of mobile Internet users.

52 Agricultural products e-commerce and second-hand e-commerce users refer to those who purchased agricultural products and second-hand goods online respectively in the last six months.

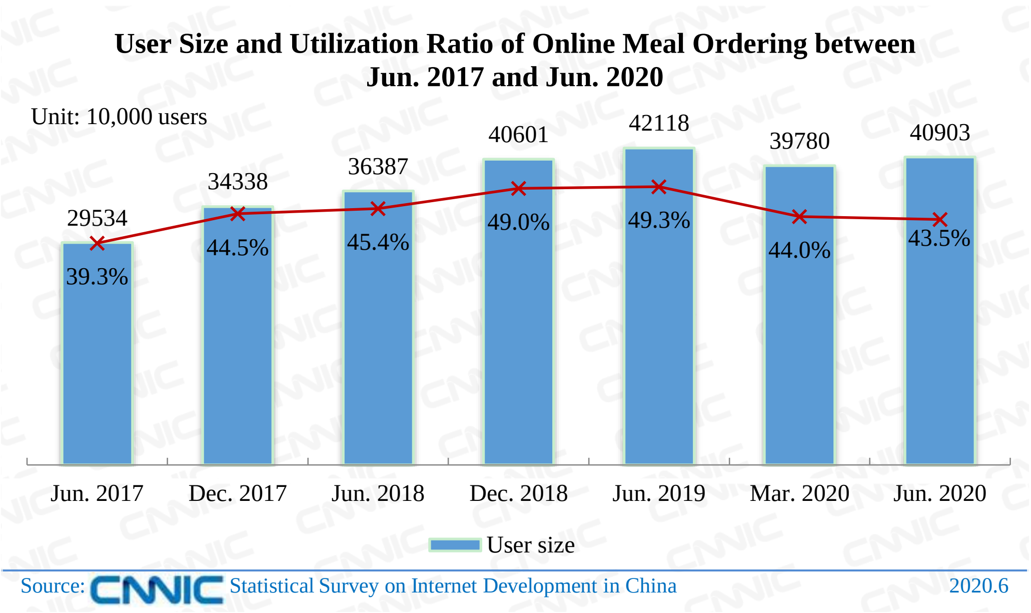

Figure 39 User Size and Utilization Ratio of Online Meal Ordering between Jun. 2017 and Jun. 2020

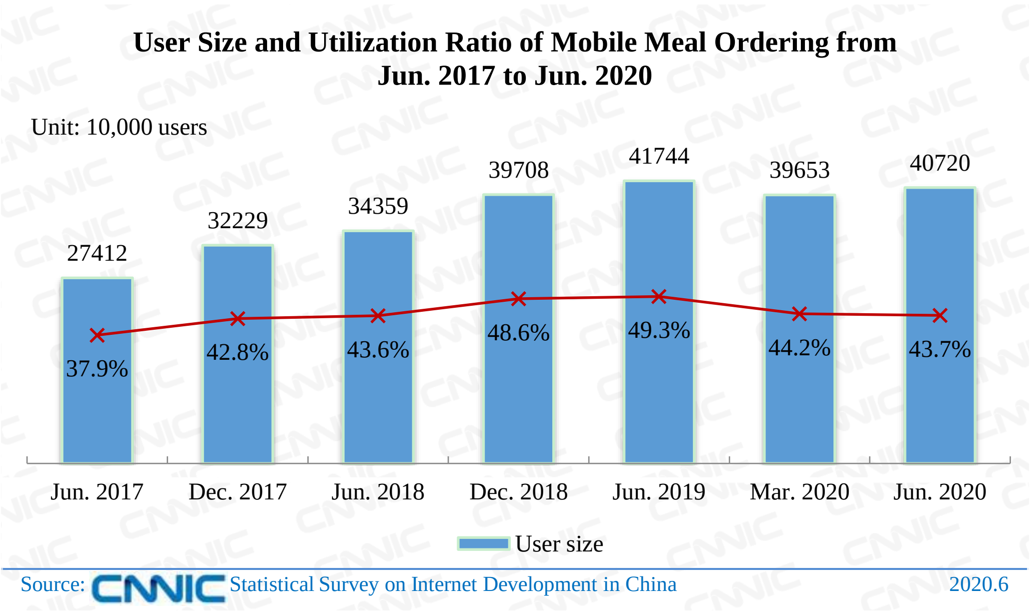

Figure 40 User Size and Utilization Ratio of Mobile Meal Ordering from Jun. 2017 to Jun. 2020

Regarding meal ordering and other services, the industry bottomed out and bounced back in the first half of 2020 after short-term shocks. First, the COVID-19 outbreak has a short-term negative impact on the meal ordering industry. During the epidemic, some food and beverage outlets and communities were closed, which negatively impacted the scale of takeaway and platform revenue as users reduced demand for takeaway. According to the data[53], Meituan Dianping’s meal ordering revenue decreased by 11.4% year-on-year in the first quarter of 2020.

[53] Source: Meituan Dianping’s financial report in Q1 2020.

Second, labor costs and average transaction values are enhanced to help the online meal ordering industry pick up. Affected by the epidemic, takeaway rider costs have been reduced. Meanwhile, more medium- and high-end brand restaurants have started their takeaway services, improving the average transaction value of takeaway to some extent. Labor costs and average transaction values have been enhanced to exert a positive impact on the takeaway pick-up. According to the data[54], Meituan Dianping's takeaway revenue grew 13.2% year-on-year in the second quarter. **Thirdly, the new business of local life embraces opportunities for growth.** Amid the epidemic, the demand for new business such as grocery shopping and flash sale surged rapidly in the first half of 2020. The high growth provided a valuable window of time for online meal ordering platforms to continuously refine their supply chains with their scale advantages, laying a solid foundation for medium- and long-term business leaps.

Regarding the social impact of the online meal ordering industry, its role in supporting the catering industry is further heightened. First, the online meal ordering industry supported the steady development of the catering industry amid the epidemic. During the epidemic, the online meal ordering industry became an important channel for catering companies to maintain operations and stabilize cash flow. According to the data,[55] China added 106,000 takeaway-related enterprises[56] from January to May 2020, up 766% year-on-year from 2019, based on the industrial and commercial registration. In particular, the number of new takeaway-related businesses in both April and May 2020 exceeded 43,000, already surpassing the number added in 2019. The impact of the epidemic on offline dine-in has accelerated the push for some restaurant brands to join the online meal ordering industry for development. **Second, the online meal ordering industry drives new spending in the catering industry.** By influencing the demand side imperceptibly, the online meal ordering industry has created more opportunities for revenue growth in the catering industry. On the one hand, takeaway drives the demand for food and beverage in niche scenarios such as afternoon tea and evening snacks; on the other hand, users increase their food and beverage expenditures because of convenient takeaway services. According to the data[57], 75% of the total revenue increment and 65% of the total profit increment in the catering industry are driven by the takeaway business, respectively.

(III) Online payment

As of June 2020, the user size of online payment was 805 million or 85.7% of China's total netizen population, up 37.02 million over March 2020; the number of mobile payment users stood at 802 million, up 36.64 million from March 2020, representing 86.0% of mobile Internet users.

[54] Source: Sina Finance, http://finance.sina.com.cn/stock/relnews/hk/2020-08-25/doc-iivhuipp0498069.shtml, August 25, 2020.
[55] Source: hexun.com, http://news.hexun.com/2020-06-05/201503716.html?_t=t, June 5, 2020.
[56]Takeaway-related enterprises refer to those whose business scope includes "takeaway" and whose status is in operation, in existence, moving in or moving out.
[57]Source: *Study on the Role of Takeaway Business in the High-quality Development of the Catering Industry* by Academy of China Council for the Promotion of International Trade.

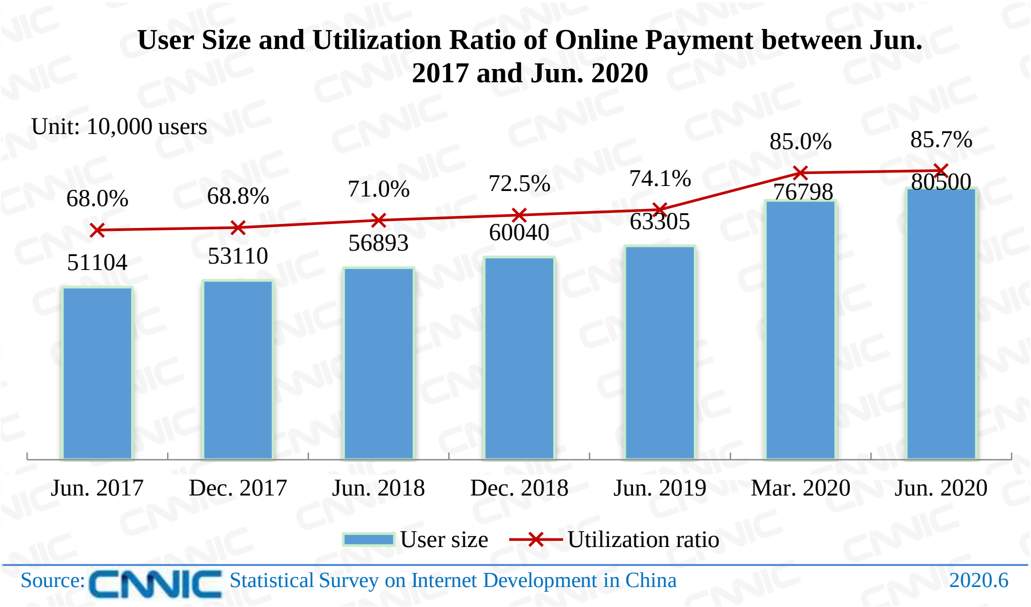

Figure 41 User Size and Utilization Ratio of Online Payment between Jun. 2017 and Jun. 2020

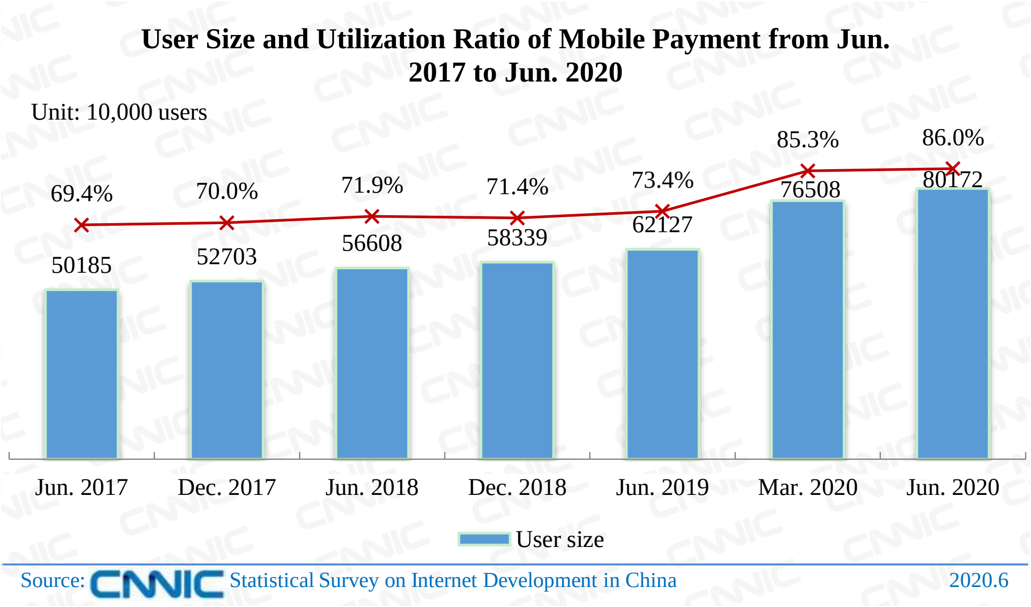

Figure 42 User Size and Utilization Ratio of Mobile Payment from Jun. 2017 to Jun. 2020

In the first half of 2020, China led the world in the scale of mobile payment transactions. With the diversified development of online payment models, the compliance of payment business was accelerated to constantly improve the operation of the entire industry.

China has continued to expand the application scenarios of mobile payment, ranking the world's No. 1 in the transaction scale for three consecutive years. First, the application scenarios of mobile payment are constantly enriched. Payment institutions help the "small shop economy" flourish by means of integrated online and offline payment, national welfare subsidies, and online training and guiding for merchants. Also, payment institutions use big data, AI and other new technologies to promote the development of "credit county" and "county-wide financial inclusion" and expand more "plus payment" application scenarios. **Second, the scale of mobile**

payment transactions continues to expand. During the COVID-19 epidemic, offline merchants accelerated their transformation to online business, and mobile payment tools played the role of information carriers, electronic wallets, credit media, and cash register and bookkeeping to promote the popularity of mobile payment. In the first half of 2020, China amounted to 196.98 trillion yuan in its mobile payment, up 18.61% year-on-year, ranking first in the world[58].

Diversification of online payment highlights the resilience and potential of the payment market. First, China's online payment has penetrated rural and middle- and old-age groups. Diversified online payment methods, intelligent payment passwords and convenient application experience help narrow the gap of online payment and show the trend of popularization, thus enhancing the anti-risk ability of the payment market. As of June 2020, the proportion of online payment users aged 40 and above was 36.6%, up 4.5 percentage points from March 2020; the proportion of rural online payment users increased by 2.7 percentage points. **Second, aggregate payment[59] assists in the interconnection of the payment industry chain.** As a connecting vehicle for merchants, consumers, and multiple payment institutions, aggregate payment not only provides a convenient way of cash register, but also offers value-added services such as precision marketing, digital operations, and low-threshold loans. Based on the interconnection of the payment industry chain, aggregate payment has facilitated the digital transformation of offline merchants and the development of financial inclusion of "sinking market[60]."

The central bank has tightened its supervision to urge payment institutions to develop in compliance with relevant laws and regulations. First, China has stepped up its efforts to regulate payment institutions. In the first half of 2020, branches under the central bank issued to payment institutions five fines of over 10 million yuan[61]. The central bank cancelled 30-odd payment business licenses, with 237 companies still holding their licenses[62]. The central bank has taken a problem-oriented approach to improving its governance mechanism, with the strict supervision model for payment institutions normalized. **Second, the compliance management of cross-border payment has been further refined.** As of March 2020, about 15 payment institutions participating in the pilot business of cross-border foreign exchange payment have obtained their official "cross-border payment license."[63] With the qualification of cross-border payment business shifting from the pilot system to the licensing system, the entire cross-border payment market will be more orderly, healthy and standardized.

IV. Online Entertainment Applications

(I) Online games

As of June 2020, the user size of online games was 539.87 million or 57.4% of China's total netizen

[58]Source: Calculations based on the data of the People's Bank of China.
[59] Aggregate payment refers to a payment model integrating multiple payment services of banks and non-banking institutions through technical means. Common products of aggregate payment include aggregate code card, smart POS, code scanner, and code scanning box.
[60] A sinking market refers to small and medium-sized third-tier cities or below as well as rural areas in China.
[61] Source: thepaper.cn, https://www.thepaper.cn/newsDetail_forward_8072233, July 1, 2020.
[62] Source: mpaypass.com.cn, https://www.mpaypass.com.cn/pay.asp.
[63] Source: the Economic Information Daily, https://xw.qq.com/cmsid/20200327A068WX00, March 27, 2020.

population, up 8.05 million over March 2020; the number of mobile game users had reached 535.92 million, up 6.99 million from March 2020, accounting for 57.5% of mobile Internet users.

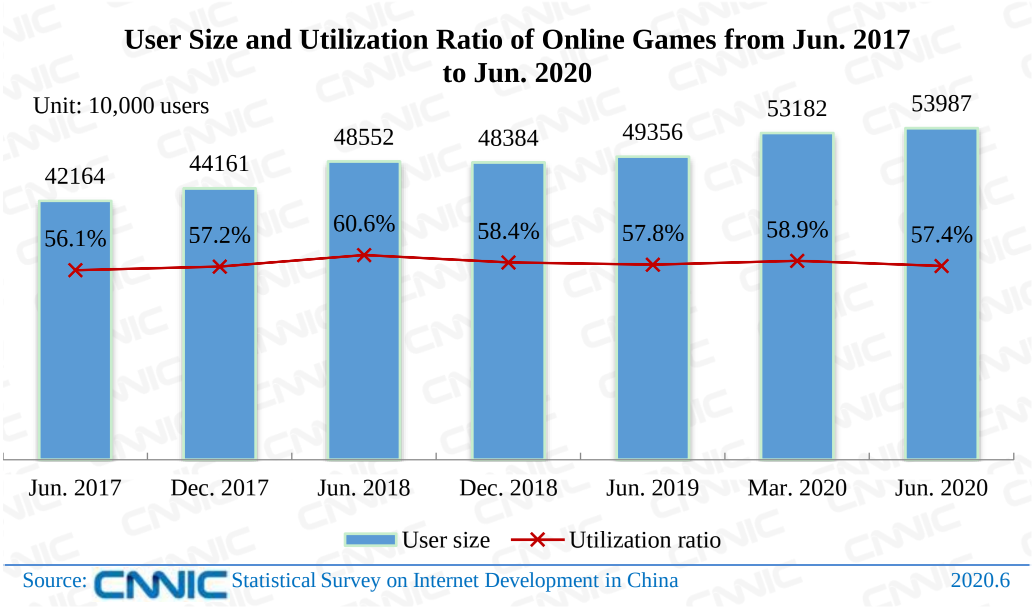

Figure 43 User Size and Utilization Ratio of Online Games from Jun. 2017 to Jun. 2020

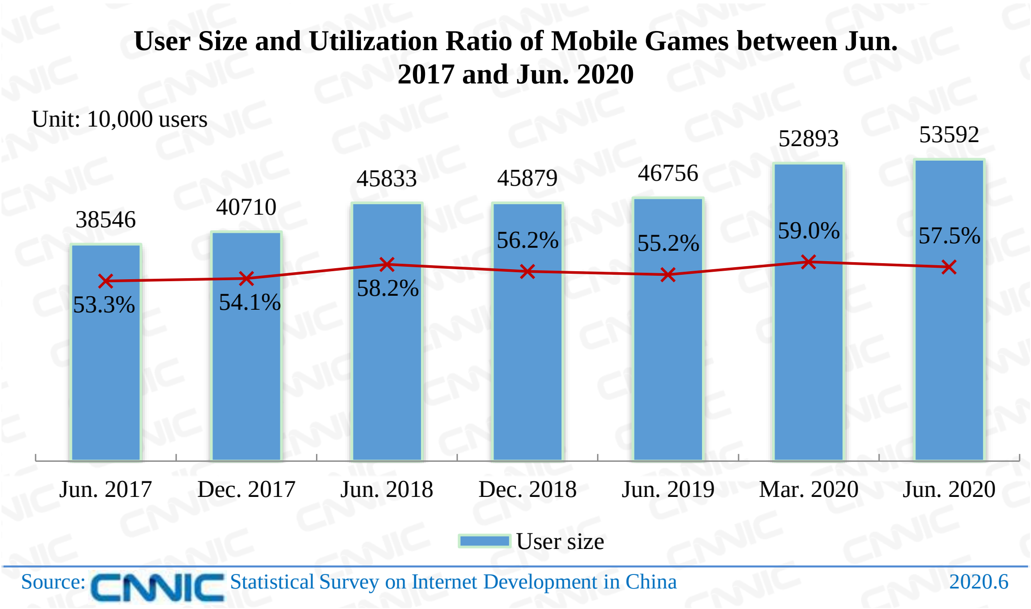

Figure 44 User Size and Utilization Ratio of Mobile Games between Jun. 2017 and Jun. 2020

In the first half of 2020, offline activities were greatly restricted by the COVID-19 epidemic, while online games met the cultural and entertainment needs of Internet users, driving both revenue and number of enterprises in the online game industry. In the meantime, China's increasingly large mobile games market provided the ground for more SMEs to innovate and develop. In addition, China's online games enterprises have further increased exchanges and cooperation in the field of mobile games with internationally renowned counterparts to boost the quality of China's mobile games, which will deliver better experience to Chinese online games users.

To meet the needs of Internet users, the online games industry has driven the growth of both

revenue and number of enterprises. The COVID-19 epidemic has severely hampered social activity, entertainment and other offline activities, prompting related activities to move online. Online games, an important means of online entertainment, met the needs of Internet users during the epidemic, contributing to the accelerated development of the online games industry. According to the data, in the first half of 2020, the actual sales revenue of the online games market was 139.493 billion yuan, up 22.34% year-on-year; the overseas revenue of China's self-developed games reached 7.589 billion U.S. dollars (or 53.362 billion yuan), an increase of 36.32% year-on-year[64]. In the first half of 2020, China added more than 22,000 games companies, with an average daily increase of 122[65]. The number of games Apps amounted to 925,000, taking up 25.8% of all mobile Apps, an increase of 26,000 from the previous month[66].

The huge mobile games market has provided the ground for SMEs to innovate and grow. According to the data, mobile games accounted for 75.04% of the total sales revenue of China's online games market in the first half of 2020, far exceeding other kinds of games[67]. The huge mobile games market and higher revenues create conditions for SMEs to grow and provide growth space for new models and games. Since 2019, a number of innovative and pioneering mobile games have gained the favor of online games users and the market, which has spurred the continued innovation and healthy development of the online games industry. Since its launch in July 2020, *Jiangnan Baijingtu*, a mobile game of simulation and management, has been among the top 50 best-selling games, and ranked No. 1 in the iOS list[68].

Games makers enhance external cooperation to improve the user experience of mobile games. In 2019, the top 10 mobile games in global user spending were bagged by China, Japan, South Korea and the US,[69] signifying that China's mobile games have been in the top tier globally. As the advantages of China's mobile games become more prominent globally, the cooperation between China's online game enterprises and their internationally renowned counterparts in related fields has been further consolidated. The cooperation between domestic and international online games enterprises is conducive to the further improvement of the quality of China's online games, which will help to promote the development of high-quality games and bring better experience to Chinese online games users. In June 2020, Tencent announced that it was co-developing the multiplayer online tactical competitive game *Pokemon Unite* with the internationally famous game maker Pokemon Company. NetEase and Activision Blizzard's mobile game *Diablo Immortal*, developed from 2019, is registered in 2020.

---

64 Source: *China Games Industry Report from January to June 2020* by the Games Working Committee of China Audio-video and Digital Publishing Association.
65 Source: Sina Technology, https://tech.sina.com.cn/roll/2020-07-14/doc-iivhvpwx5276255.shtml, July 14, 2020.
66 Source: *Operation of Internet and Related Services in the First Half of 2020* by the Ministry of Industry and Information Technology of China.
67 Source: *China Games Industry Report from January to June 2020* by the Games Working Committee of China Audio-video and Digital Publishing Association.
68 Source: sohu.com, https://www.sohu.com/a/413048459_403354?_trans_=000014_bdss_dkasgjczh, August 14, 2020.
69 Source: the *2020 Mobile Market Report* by App Annie.

(II) Online video

Up to June 2020, the user size of online video (including video clips) in China had reached 888.21 million, up 37.77 million from March 2020, making up 94.5% of all Internet users. The number of video clip users amounted to 817.86 million, up 44.61 million from March 2020, accounting for 87.0% of overall Internet users.

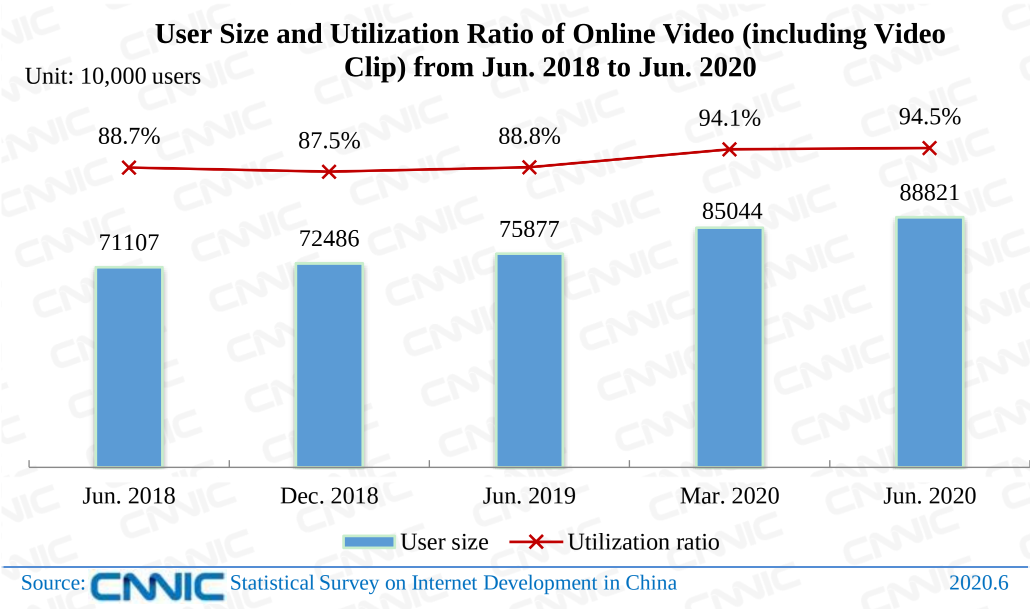

Figure 45 User Size and Utilization Ratio of Online Video (including Video Clip) from Jun. 2018 to Jun. 2020

In the first half of 2020, Internet users' entertainment needs continued to shift online, driving further growth in user size and utilization ratio of online video. Quality content is still the core competitiveness of online video platforms. Payment models based on quality content are gradually gaining user recognition.

In terms of content operation, high-quality short dramas have become the highlight of the online drama market, with platforms speeding up their theatrical operations. In February 2020, the *Notice on Further Strengthening the Management of the Creation and Production of TV Dramas and Online Dramas* was issued. Relevant administrative departments have strictly supervised the planning and establishment of online drama projects and the review of finished films, controlled watered-down dramas, guided the creation and production of high-quality online drama series, and promoted the rapid development of high-quality online dramas. In the first half of 2020, 356 online dramas were launched on major video platforms, of which online short dramas[70] accounted for 47.5%, up 12.4 percentage points over 2019[71]. Online short dramas, represented by *If There is no Tomorrow* and The Bad Kids, achieved traffic and word-of-mouth success. In addition, major platforms adopted a theatrical model to improve the ability of producing their own high-quality content and categorize their theatrical models for quality content series. By doing so, they not only

[70] Online short dramas refer to those with less than 20 episodes.
[71] Source: Guduo Media.

met the needs of different users but also realized differentiated competition by identifying their genres and positions and building their own brands.

From a business model perspective, premium video on demand[72] is being normalized, and the revenue of member services is growing steadily. Since 2019, other video platforms, except MangoTV, have been slowing down the growth rate of membership. Enhancing members' ARPU[73] has become the key direction of profitability for major platforms. Quality content and scheduling that meets users' needs hold the key to attracting users to pay for their membership. In August 2019, Tencent Video debuted a premium video on demand (PVOD) model that unlocks the unreleased episode endings in advance, which was followed by multiple online dramas on other platforms. Since March 2020, the number of PVOD dramas has increased significantly, the format and price have been fixed, and PVOD has become the normal scheduling model to open new revenue channels for video websites. In the first half of 2020, iQiyi's membership service revenue was 8.6 billion yuan, up 26.5% year-on-year, accounting for 57.3% of its total revenue[74].

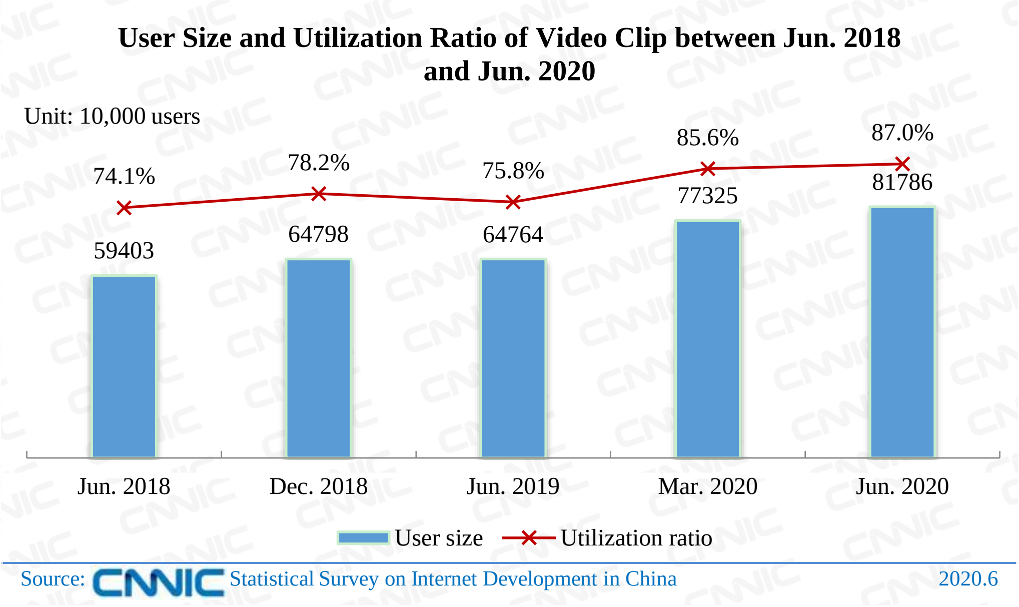

Figure 46 User Size and Utilization Ratio of Video Clip between Jun. 2018 and Jun. 2020

The integration of the video clip industry with news, e-commerce, tourism and other industries is being deepened, with communication scenarios expanding. Video clip platforms also continue to leverage their strengths to assist the rural economy with its development.

To disseminate information, video clips are increasingly becoming a basic feature of other web Apps. First, video clips are the new choice for news coverage. Video clips provide a large number of information sources, change the news narrative, broaden news reporting channels, and create new methods for news dissemination. During the epidemic, Weibo users uploaded a total of 2.25 million epidemic-related video clips, which were viewed more than 84.2 billion times[75]. **Second, video clip has become a new part of e-commerce platforms.** Major e-commerce platforms have continued to expand their video clip business, use video clips to vividly display

[72] Premium video on demand means that an online video subscriber, after his/her membership fee has been paid, makes an additional payment to unlock the unreleased video content in advance.

[73] ARPU means average revenue per user.

[74] Source: iQiyi's financial reports in 2019 and 2020.

[75] Source: Weibo.

products, promote consumers' awareness of products, stimulate user demand and improve conversion efficiency. Video clips have been part of mainstream e-commerce platforms, with the "recommendation (zhong cao in Chinese)"[76] function highlighted increasingly. **Third, video clips are the new driving force in the tourism market.** Over the past two years, video clips have enabled a host of tourist attractions to go viral, serving as an important marketing tool for the tourism industry. Major online travel platforms have introduced video clip content communities to guide users to create video clips about their trips so as to increase their traffic and finally monetize it.

As a mainstream web application, video clip platforms are exploring new models for supporting agriculture. Video clip platforms have taken actions to solve production and business problems for farmers and boost the development of rural economy through content support, traffic inclination, marketing assistance and brand empowerment. Key video clip platforms now cover the entire agricultural industry chain linking farmers, agronomy experts and enterprises, creating an online community for exchange, learning and trading. During the epidemic, Kuaishou released its agricultural courses in large volumes, unveiled its "Spring Farming Plan," and supported agricultural video clips with 500-million-yuan traffic. In the meantime, it allowed offline enterprises to make their sales through online e-commerce, lending a helping hand to the whole chain of agricultural production and operation in an all-scenario manner[77]. Douyin and other platforms launched a series of activities to assist farmers in improving the efficiency of matching supply and demand information of agricultural products across China and in solving the problem of selling agricultural products.

(III) Live streaming

As of June 2020, the user size of live streaming in China had reached 562.30 million, up 2.48 million from March 2020, taking up 59.8% of all Internet users. Specifically, the user size of livestream e-commerce was 309 million, up 44.30 million from March 2020, accounting for 32.9% of the overall Internet users. That of live game streaming was 269 million, up 9.23 million over March 2020, making up 28.6% of all Internet users. That of host live show was 186 million, down 21.15 million from March 2020, taking up 19.8% of all Internet users. That of live concert streaming was 121 million, down 29.47 million from March 2020, representing 12.8% of all Internet users. That of live sport broadcasting was 193 million, down 19.27 million over March 2020, accounting for 20.6% of overall Internet users.

[76] Recommendation (zhong cao in Chinese) is an Internet term for sharing and recommending a product through content introduction and display to stimulate others' desire to purchase it.
[77] Source: xinhuanet.com, http://www.xinhuanet.com/tech/2020-02/21/c_1125605562.htm, February 21, 2020.

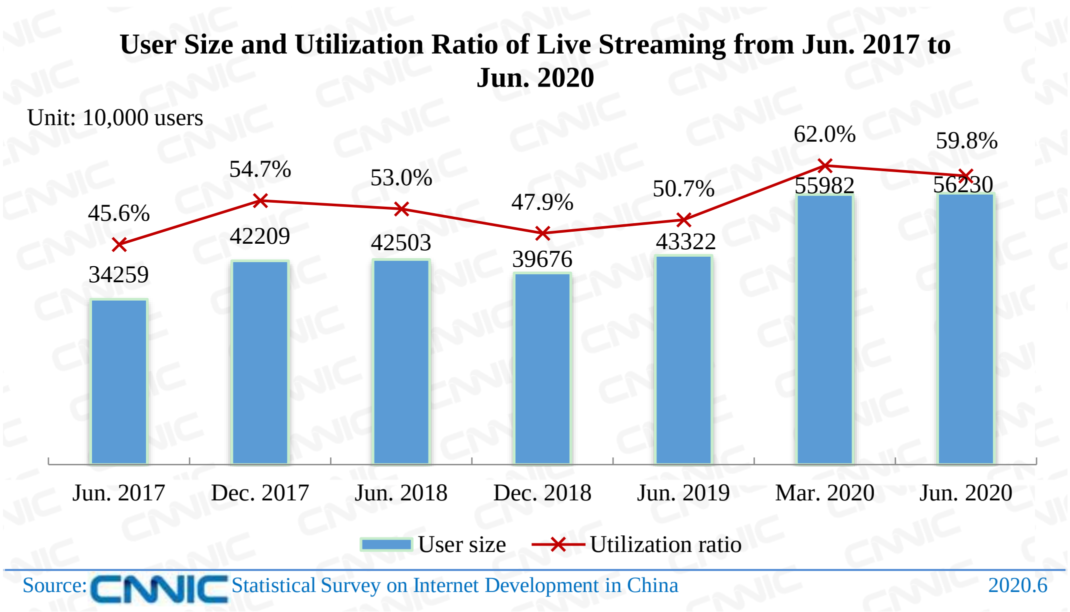

Figure 47 User Size and Utilization Ratio of Live Streaming from Jun. 2017 to Jun. 2020

In the first half of 2020, the COVID-19 epidemic had a marked impact on the live streaming industry in the following two aspects.

First, live-stream e-commerce has become one of the fastest growing Internet applications in the first half of 2020. On April 20, 2020, General Secretary Xi Jinping encouraged local staff members for live-stream e-commerce that **"e-commerce is promising in marketing agricultural and sideline products,"** while researching poverty alleviation in Jinmi Village, Zhashui County, Shaanxi Province, According to the data, over 10 million live e-commerce broadcasts were hosted by more than 400,000 active live streamers in the first half of 2020, attracting over 50 billion viewers. The vigorous development of live-stream e-commerce is attributed largely to the following three factors. **At the government level,** live-stream e-commerce has become a new growth point for governments at all levels to boost the economy and consumption. In response to the changes in domestic and international markets during the epidemic, the CPC Central Committee clearly proposed the establishment of a new development paradigm with domestic circulation as the mainstay and domestic and international circulations reinforcing each other. In this context, live-stream e-commerce has done a good job of stimulating consumption potential, serving as an important force in facilitating the great circulation of domestic demand. Local governments in Zhejiang, Guangdong and Sichuan have spurred and guided this new form of business and supported the development of live-stream e-commerce in their jurisdictions by issuing preferential policies, attracting professional talents and building industrial parks. **At the enterprise level,** major Internet companies engaging in live-stream e-commerce has boosted the rapid growth of this industry. In the first half of the year, live-stream e-commerce became a focused area for Taobao, Pinduoduo and other e-commerce platforms; Douyin, Kuaishou and other video clip platforms; and Baidu, Sohu and other traditional Internet companies. They pooled a wealth of talent, funds and media resources in a short period to make for the explosive growth of live-stream e-commerce in the first half of 2020. **At the user level,** live-stream e-commerce stimulated online shopping demand during the epidemic. By integrating itself with the goals of fighting the epidemic and lifting farmers out of poverty, it enhanced users' sense of gain during their shopping. The public welfare

campaign titled "Thank you for Purchasing Hubei's Products," co-hosted by CCTV's news anchor and professional live streamer on Weibo, attracted 10.91 million users to watch online concurrently so as to promote Hubei's characteristic products, with sales exceeding 40 million yuan each time[78].

Second, the live game streaming has continued its overall positive momentum. As a traditional type of live streaming, live game streaming functioned as an important way for legions of users to relax themselves at home amid the epidemic, driving further growth in revenue and mobile user activity on live game streaming platforms. According to the data[79], the revenue of Douyu and Huya increased by 42.4% and 40.3% year-on-year, respectively, in the first half of 2020. In terms of user activity, both companies maintained their year-over-year growth of average monthly active mobile users by over 15% in the first and second quarters.

V. Public Service Applications

(I) Online Car-hailing Services

As of June 2020, the number of online car-hailing users in China reached 340.11 million, down 22.19 million from March 2020, accounting for 36.2% of overall Internet users.

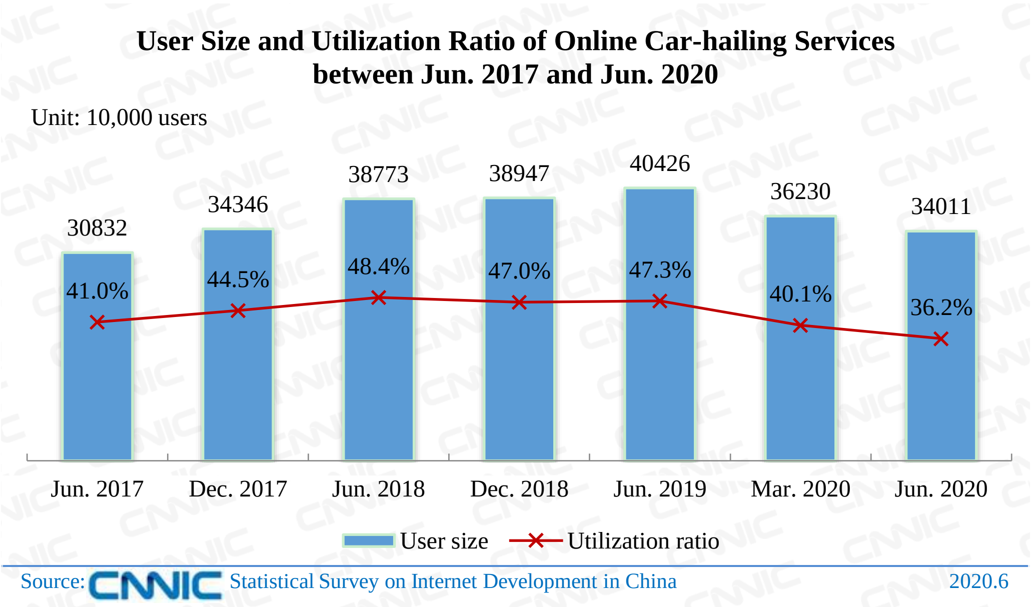

Figure 48 User Size and Utilization Ratio of Online Car-hailing Services between Jun. 2017 and Jun. 2020

In the first half of 2020, online car-hailing companies explored new models and business forms that benefit all. The entire industry has stepped up efforts to ensure safe travel and continuously enhance social benefits.

In terms of market operations, first, companies are fully tapping into the potential of cross-

[78]Source: Weibo.
[79]Source: the Q1 and Q2 2020 financial reports released by Douyu and Huya.

border integration and autonomous driving to explore new models. Auto manufacturers and online car-hailing companies are working together in a model of "hardware plus soft strength." The former has the advantage of car manufacturing capability and channel resources, while the latter has the advantage of digital technology capability and user resources. The deep integration of the two will reshape the online car-hailing market. In addition, online car-hailing companies have further increased investments in the intelligent field and launched L2 and L3[80] autonomous driving services to integrated themselves into the smart driving industry chain. Going forward, companies will continuously expand their survival space by improving their intelligence and optimizing their capabilities for operations and services. **Second, online car-hailing companies have attracted "longtail[81] market" users to expand new business.** Recently, meeting the needs of "longtail market" has become a new goal of online car-hailing companies. For one thing, online car-hailing platforms continue to aggregate[82] the capacity of service providers. Didi Chuxing has introduced a one-click call model for multiple travel service providers after Gaode Map and Meituan Dianping aggregated service providers. For another, the platforms have tapped the potential of business and consumer services. To meet the needs of government and businesses, online car-hailing companies provide dedicated car, commuter car and customized charter services. To satisfy the needs of consumer services, they also expand freight services within the same city to offer instant delivery services.

With regard to social benefits, first, the online car-hailing industry has abided by the bottom line of safety, with the safety of users guaranteed. Amid the epidemic, online car-hailing companies continued to upgrade their safety strategies, such as nucleic acid testing for drivers, installation of protective film for vehicles, and the introduction of "smart anti-epidemic code[83]," so as to safeguard the health of drivers and passengers. In addition, the safety standard for online car-hailing services[84] first released by the China Communications and Transportation Association covers basic safety functions such as trip sharing, 110 alarm, emergency contact, trip recording, and phone number protection, which helps standardize the development of the industry and maintain social security and stability. **Secondly, the online car-hailing industry actively participates in public welfare activities to offer its support to medical staff.** Online car-hailing companies organized transportation capacity to fully support the fight against the epidemic by setting up their vehicle fleets[85] for medical staff in 10-odd cities such as Wuhan, Beijing and Nanjing, with about 160,000 drivers voluntarily serving 37,987 medical workers and driving over 15 million kilometers[86].

---

[80] According to the Society of Automotive Engineers (SAE), autonomous driving is divided into five categories including L0 (no automation), L1 (driver assistance), L2 (partial automation), L3 (conditional automation), L4 (high automation) and L5 (complete automation).

[81] Long-tail: with small sales but diverse forms, a product or service that was previously under-appreciated is likely to outperform mainstream products because of its large total volume and cumulative total revenue.

[82] Aggregate: a platform expands supply-side resources, accesses multiple online car-hailing service providers and aggregates traffic portals in a bid to enhance the efficiency of hailing cars online.

[83] Smart anti-epidemic code: passengers can scan a code via their mobile phones to view disinfection records of the vehicle they ride or register their ridings.

[84] Safety standard for online car-hailing services refers to the *Self-regulation Code for Safe Operation of Online Car-hailing Platforms and Companies* and the *Technical Specifications for Safe Operation of Private-Minibus Shared-Riding Information Service Platforms and Companies*.

[85] Source: qq.com, https://tech.qq.com/a/20200225/029298.htm, February 25, 2020.

[86] Source: Didi Chuxing.

(II) Online education

As of June 2020, the user size of online education was 380.60 million or 40.5% of China's total netizen population, down 42.36 million over March 2020; the number of mobile learning users had reached 376.68 million, down 43.55 million from March 2020, making up 40.4% of mobile Internet users. In the second quarter of 2020, with the epidemic contained on an ongoing basis, the scale of online education users declined as primary and secondary schools and universities resumed their classes in an orderly manner.

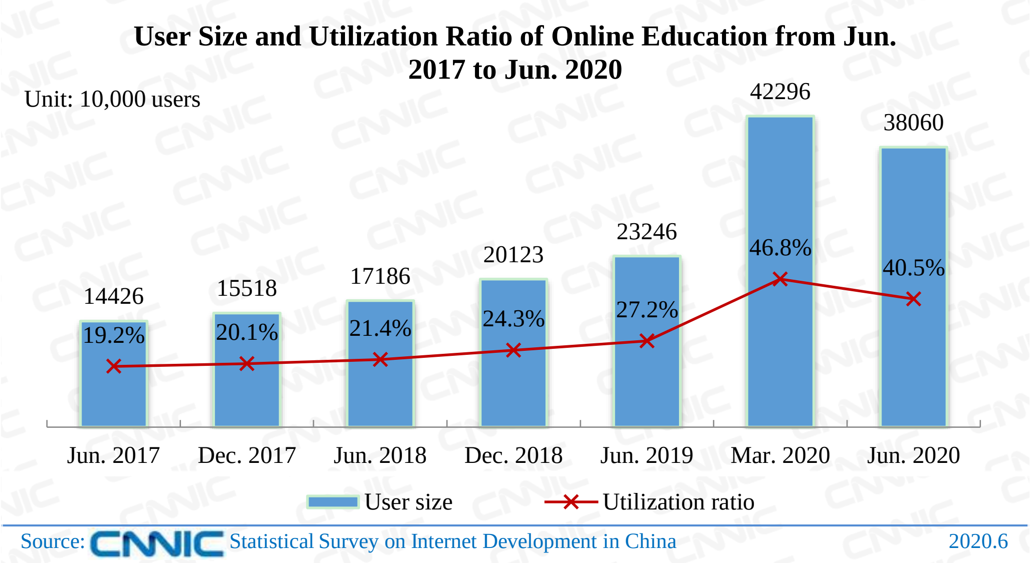

Figure 49 User Size and Utilization Ratio of Online Education from Jun. 2017 to Jun. 2020

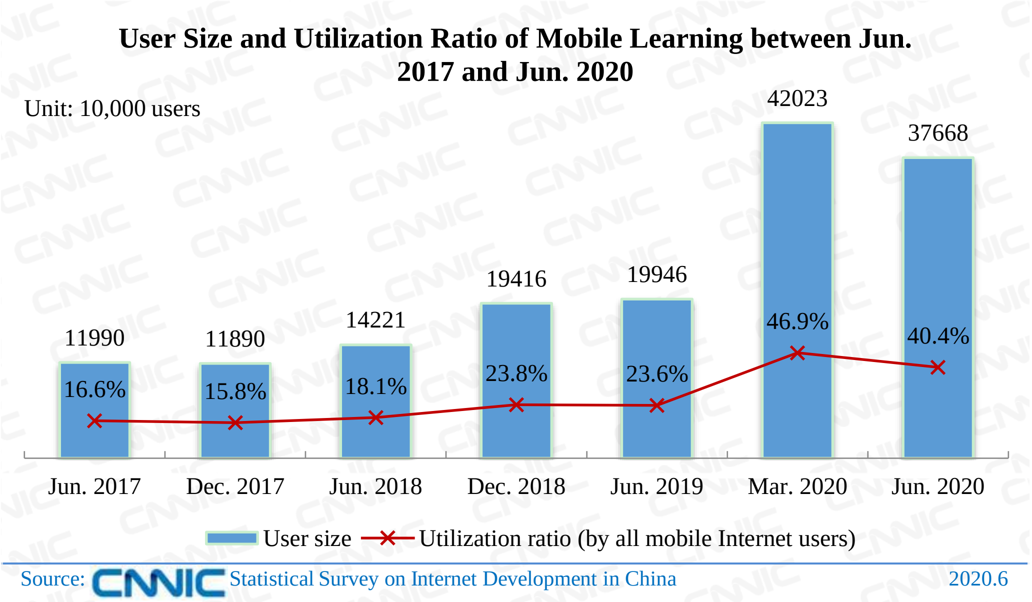

Figure 50 User Size and Utilization Ratio of Mobile Learning between Jun. 2017 and Jun. 2020

In the first half of 2020, under the guidance and promotion of the policy of "suspended classes and

non-stop learning," 282 million students nationwide[87]generally switched to online courses, with the level of IT-based education further enhanced. In the future, the online and offline integration will become a trend in the education industry.

From a supply-side perspective, online education and IT-based education reinforce each other. First, the results of basic IT-based education have laid a solid foundation for the development of large-scale online education. In 2019, 98.4% of primary and secondary schools (including teaching points) nationwide had the access to the Internet. 90.1% of primary and secondary schools had multimedia classrooms, with 10 million teachers engaging in the program of "one teacher producing one excellent course and one lesson taught by one famous teacher[88]." These results have been fully applied and tested in the epidemic prevention and control, laying a solid foundation for the promotion of online education. **Second, the widespread application of online education has substantially promoted the practice of IT-based education.** Prior to the epidemic, the products and applications of in-school IT-based education were mostly designed with auxiliary functions for achieving collaboration between schools, parents, and students, and less often involved the output of teaching content. During the epidemic, schools, governments and third-party companies and platforms across China launched online courses timely to enable a real shift to educational innovation.

On the user side, the public's awareness and use of online education increased rapidly during the epidemic. First, local education departments are proactively promoting the use of e-learning platforms. In February 2020, the Ministry of Education and 27 provinces, following the issuance of the Circular on Working Arrangements for the "Suspended Classes and Non-stop Learning" Period in Response to Postponed Start of Primary and Secondary Schools, opened national and provincial e-learning platforms to provide reliable services for students studying at home. As of May 11, the National Primary and Secondary School Web Cloud Platform had 2.073 billion viewers and 1.711 billion visitors[89]. **Second, major online education platforms are accelerating their penetration of sinking market.** Major online education platforms had responded to the government's call to launch free live courses for students, with the user size growing rapidly. During the epidemic, the number of daily active users in the online education industry rose from 87 million on weekdays to 127 million after the Spring Festival, an increase of 46%, with new traffic mainly coming from third, fourth and fifth-tier cities[90]. As of June 2020, online education users in third-tier cities and below accounted for 67.5% of overall users, an increase of 7.5 percentage points year-on-year. Also, it should be noted that, despite the accelerated penetration of online education in the sinking market during the epidemic, there is still much room for improvement in the accessibility and content quality of education services, and there is still a long way to go before achieving universal education in remote areas.

OMO[91] will emerge as the mainstream model in the development of the education industry. Online education can break through the time and space constraints, promote resource sharing and achieve fair education. Offline education is more conducive to teacher-student interaction, yielding

[87]Source: the Ministry of Education of China, http://www.moe.gov.cn/jyb_sjzl/s5990/202008/t20200831_483697.html, August 31, 2020.

[88] Source: the Ministry of Education of China's press conference, http://www.moe.gov.cn/fbh/live/2020/51987/, May 14, 2020.

[89] Source: the Ministry of Education of China's press conference, http://www.moe.gov.cn/fbh/live/2020/51987/, May 14, 2020.

[90] Source: Quest Mobile.

[91] OMO refers to Online Merge Offline.

good teaching results. The integration of online and offline education is the general trend in the future. In July, the National Development and Reform Commission, the Office of the Central Cyberspace Affairs Commission, the Ministry of Industry and Information Technology and other ten departments jointly issued the *Opinions on Supporting the Healthy Development of New Forms and Models of Business and Activating the Consumer Market to Expand Employment*, making clearly that convergent online education should be developed vigorously to build an integration mechanism for online and offline education in a bid to develop a sound interactive model. In the future, as relevant government departments improve the intellectual property protection, content supervision, market access and other institutional norms for online education, schools can gradually explore the inclusion of excellent online resources into the daily teaching system and carry out classroom teaching based on a smart online environment in an effort to achieve a higher goal of educational training and output.

(III) Online medical services

In the first half of 2020, users' demand for online medical services was growing due to the epidemic, further promoting the online development of China's healthcare industry. In this context, the complementary role of online medical services to the offline healthcare system was highlighted.

The online medical market is expanding at a faster pace, while the capacity for service supply is being enhanced. Up to June 2020, 276 million Internet users or 29.4% of all netizens had received online medical services in China. **First, new technologies have put into place the new infrastructure for medical services.** On the supply side, online medical enterprises, relying on big data, cloud computing, AI and other new technologies, continue to expand and explore in smart healthcare and cooperate with the government, hospitals, research institutes and other external institutions to carry out smart medical services based on AI, big data and information technology. During the epidemic, a team led by Academician Zhong Nanshan and Tencent announced a partnership to establish a joint laboratory for AI and big data to research screening, early warning and prevention and control for epidemic, respiratory and thoracic diseases. **Second, the payment model is maturing, and the industry-specific ecosystem for healthy development is gradually forming.** On the operational side, Internet companies have begun to explore service models to vigorously promote the development of paid medical industries including medical cosmetology, dentistry, medical checkup, vaccines, pregnancy and childbirth. They have continuously upgraded online medical consumption, developing a healthy ecosystem. According to the data[92], Ali Health's revenue from health consulting and other online medical services totaled 38.42 million yuan, up 221.2% year-on-year.

Online medical industry has introduced its policies, diversifying service entities. As of June 2020, 26.4% of Internet users had purchased medical supplies including drugs and health devices online; 17.9% had used online medical services such as registration and consultation due to the epidemic. Netizens' acceptance of online medical services has been increasing. **First, policies have facilitated the integration of online medical services into the medical insurance payment system to help promote universal sharing of online medical care.** With the introduction of policies, medical insurance information security, privacy, electronic credentials of medical insurance payment, security of electronic signature, electronic bills and other issues are being resolved, with online medical benefits for people further shared. On February 26, the Wuhan Medical Security Bureau opened medical insurance payment for WeDoctor Digital General Hospital, making it the first platform-based Internet hospital in Wuhan to include medical insurance payments. Subsequently, multiple provinces and cities temporarily included online medical treatment in their medical insurance payments during the epidemic, including Zhejiang, Tianjin, Jiangsu and Shanghai. **Second, the quality resources of traditional medical institutions have been extending online, driving the growth of users.** The medical industry has used the "Internet plus" initiative to optimize resources allocation, enhance service efficiency, make more hospitals highly engaged and have highly qualified doctors practice medicine. According to the data[93], nearly 600 Internet hospitals were established in all provinces of China, with the approval of the National Health Commission. **Thirdly, the number of visits to Internet platforms, represented by**

---

[92] Source: Ali Health's financial report 2020.
[93] Source: the National Health Commission of China.

Ping'an Doctor, Ali Health and Good Doctor, has increased significantly, with user habits being formed. In response to the epidemic, some third-party Internet service platforms increased the number of medical consultations 20-odd times year-on-year, with the number of prescriptions increasing nearly 10 times[94]. Multiple platforms launched online appointments for nucleic acid testing services of COVID-19, greatly improving the efficiency of results feedback.

[94] Source: Press Conference of Joint Prevention and Control Mechanism of the State Council of China, March 20, 2020.

Chapter Four Development of E-government

I. Development of E-government Services

As of June 2020, the user size of China's e-government services reached 773 million or 82.2% of overall Internet users, up 11.4% from March 2020.

The "Internet plus government services" has given a strong boost to the post-epidemic resumption of work and production. After the COVID-19 outbreak, "zero meeting, zero legwork and zero cost" had become the most fundamental requirements for the nationwide anti-epidemic efforts as well as a strong driving force for the development of China's digital government. The importance of "Internet plus government services" continued to be highlighted as various industries across China resumed work and production. **For one thing, governments at all levels are going digital to ensure that economic development goes hand in hand with the fight against the epidemic.** The national platform for government services has set up a service column for micro and small enterprises and self-employed businesses to make policies more accessible and go through procedures at one stop, facilitate the resumption of work and production, and ensure services were put in place during the epidemic. **For another, online services have improved the working efficiency and accelerated the resumption of work and production.** Online administrative approval services have been available in many places to open the green approval channel for the fight against the epidemic and strive for "zero meeting, zero legwork and zero cost," so that enterprises can resume their work and production more efficiently. The national platform for government services has developed a health QR code, gathering and sharing 623 million health QR codes across the country. With a cumulative total of 600 million persons served, health QR codes functioned as a pass in most areas of the country, representing an important innovation of the big data-based response to the epidemic.

The fully integrated system of government services has been established initially. According to the UN data[95], China has an e-government development index of 0.7948, ranking 45th, a record high signaling the "very high" level of global e-government development. But it ranked 65th in 2018 in this regard. **For one thing, local governments have been promoting the cross-regional access to more government services via a single website.** The Beijing-Tianjin-Hebei region, the Yangtze River Delta and other regions have already begun to deploy the cross-regional access to more government services via a single website, with early pilot projects focusing on enterprise investment approval as well as social security and housing provident funds for employees. Cross-regional one-stop websites combine the inter-regional policy system with new technologies to better promote cross-regional cooperation by optimizing the inter-regional business environment. They also integrate government services as many as possible to speed up the rational allocation of market factors and resources. **For another, a five-tier online service system has taken shape initially to**

[95] Source: the *United Nations E-Government Survey Report 2020*.

cover urban and rural areas, with effective coordination at all levels. Nearly 70% of China's provinces have achieved the five-tier coverage for provincial, municipal, county, township and village services so that more villages have access to government services. Equal and universal government services have been available. The contradiction between the effective supply of e-government services and the insufficient and unbalanced demand of enterprises and people has been alleviated to some extent. Government services benefiting the wellbeing of people have been made available.

II. Development of Government Websites

I. General Situation of Government Websites by Province

As of June 2020, there were 14467 government websites[96] in China, mainly including government portals[97] and departmental websites[98]. Specifically, one website was designed for the Chinese government. Departments under the State Council and their internal and vertical administrative agencies had 900 government websites. 13,566 government websites for administrative units at the provincial level or below were distributed in 31 provinces (autonomous regions and municipalities directly under the central government) and Xinjiang Production and Construction Corps.

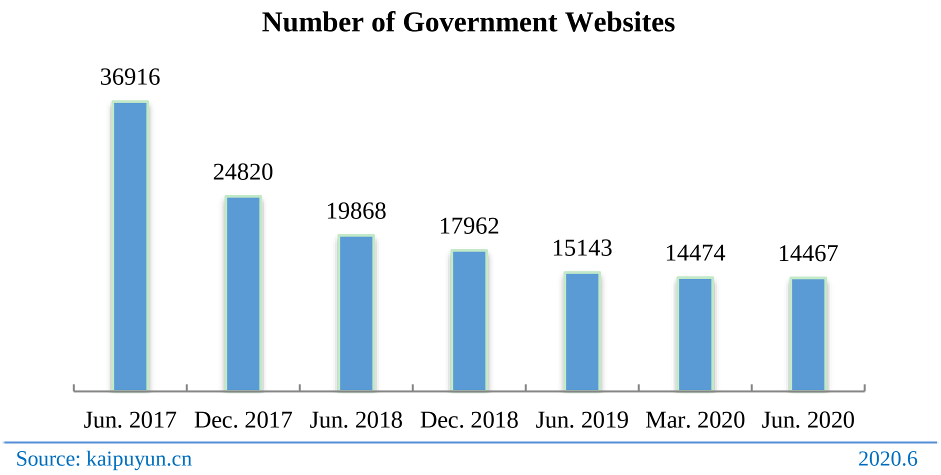

Figure 51 Number of Government Websites

Table 5 Number[99] of Government Websites by Province from Dec. 2019 to Jun. 2020

---

[96] Government websites refer to those run by people's governments at all levels and their departments, agencies and institutions with administrative functions on the Internet. They have the functions of information release, interpretation and response, service, and interactive exchange.
[97] Government portals refer to government portal websites set up by people's governments at or above the county level and departments under the State Council. In principle, villages, towns and communities do not set up government portals, and there are special treatment for special needs.
[98] Departmental website: provincial and municipal government departments, as well as institutions above the county level where the system-wide vertical management department is located, can set up their own websites. In principle, county-level government departments do not set up government websites, and there are special treatments for special needs.
[99] The data Table 7 do not include the number of websites of ministries and commissions.

Province	Jun. 2020	Dec. 2019
**Beijing**	73	72
**Tianjin**	97	105
**Hebei**	502	499
**Shanxi**	416	398
**Inner Mongolia**	553	537
**Liaoning**	554	543
**Jilin**	311	302
**Heilongjiang**	198	207
**Shanghai**	67	63
**Jiangsu**	649	645
**Zhejiang**	570	558
**Anhui**	837	810
**Fujian**	435	433
**Jiangxi**	536	533
**Shandong**	894	864
**Henan**	842	841
**Hubei**	608	707
**Hunan**	595	576
**Guangdong**	587	617
**Guangxi**	561	573
**Hainan**	110	108
**Chongqing**	87	113
**Sichuan**	920	909
**Guizhou**	425	413
**Yunnan**	302	302
**Tibet**	225	215
**Shaanxi**	615	627
**Gansu**	521	520
**Qinghai**	133	134
**Ningxia**	128	126
**Xinjiang**	162	161
**Xinjiang Production and Construction Corps**	53	51
**Total**	13566	13562

Source: Kaipuyun

II. Number of Government Websites by Administrative Level

As of June 2020, departments under the State Council and their internal and vertical institutions had 901 government websites[100], accounting for 6.2% of the total. There were 11,866 government websites for administrative units at the municipal level or below, making up 82.0%.

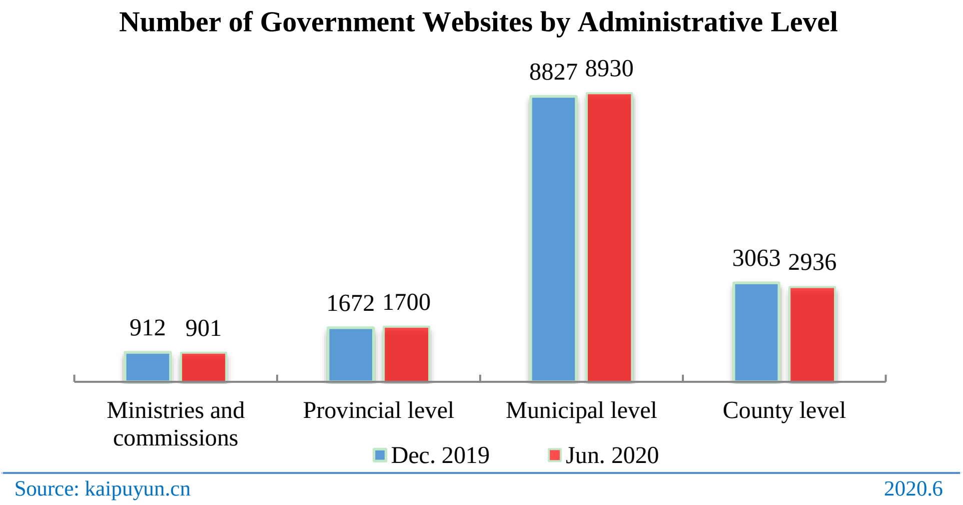

Figure 52 Number of Government Websites by Administrative Level

III. Number of Columns on Government Websites by Administrative Level

As of June 2020, a total of 294,000 columns were opened on government websites at all administrative levels, covering information disclosure, online services, and government affairs. Among the government websites at all administrative levels, municipal websites set the largest number of columns, reaching 131,000, accounting for 44.5% of the total. Among all columns of government websites, the number of columns of information disclosure was 209,000, ranking first and accounting for 71.2%; Online service columns took up 12.9%; Government affairs column made up 11.8%.

[100]This data include the website of the Chinese government.

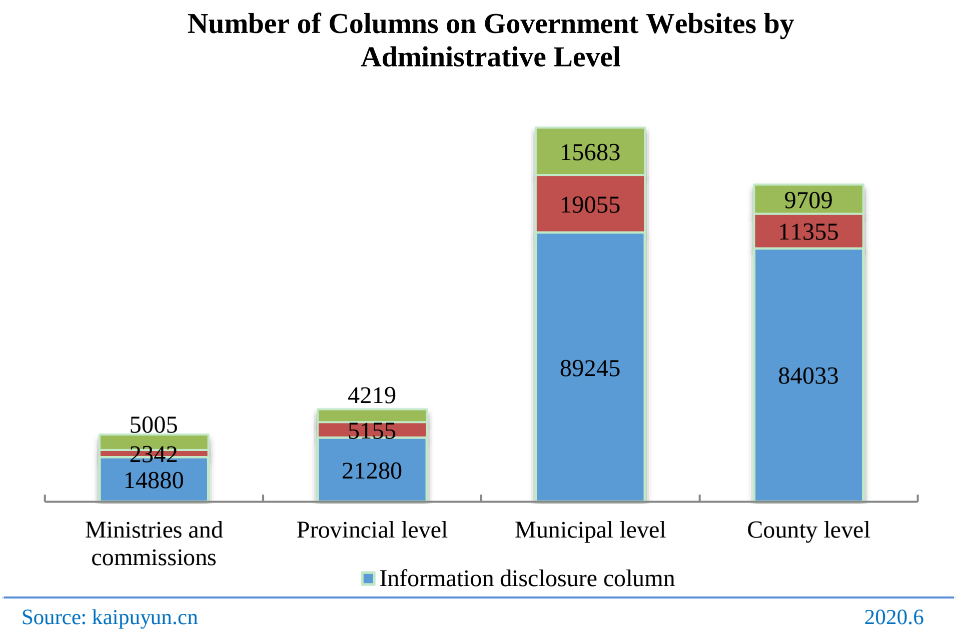

Figure 53 Number of Columns on Government Websites by Administrative Level[101]

IV. The number of articles updated on the homepages of government websites at all administrative levels

In the first half of 2020, the number[102] of articles updated on the homepages of China's government websites increased by 20.1% over the end of 2019. The increase in the number of articles updated on the homepage of county government websites was 30.7%, the highest figure.

[101] The number distribution of columns on government websites at all administrative levels only includes the three categories shown in the Figure, excluding other small columns.
[102] The number of articles updated on the homepage refers to that of updated homepage articles on government websites.

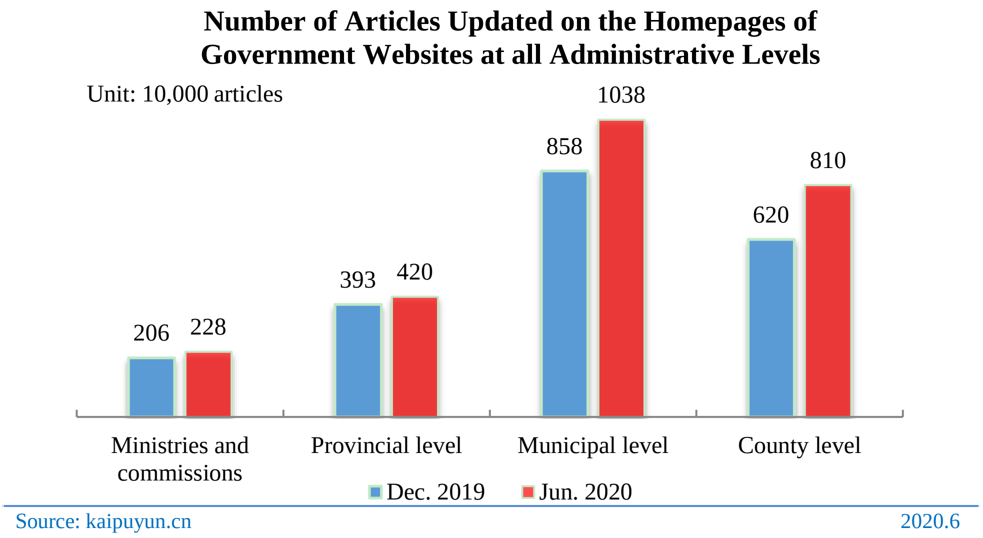

Figure 54 Number of Articles Updated on the Homepages of Government Websites at all Administrative Levels

III. The Development of New Government Media

(I) The Development of Government Service Search

1. Overall Status of Government Service Search

In the first half of 2020, the search volume of government services via Baidu App was 10.779 billion times. As of June 2020, the cumulative number of people served by Baidu's mobile smart government and livelihood applet was 5.515 billion.

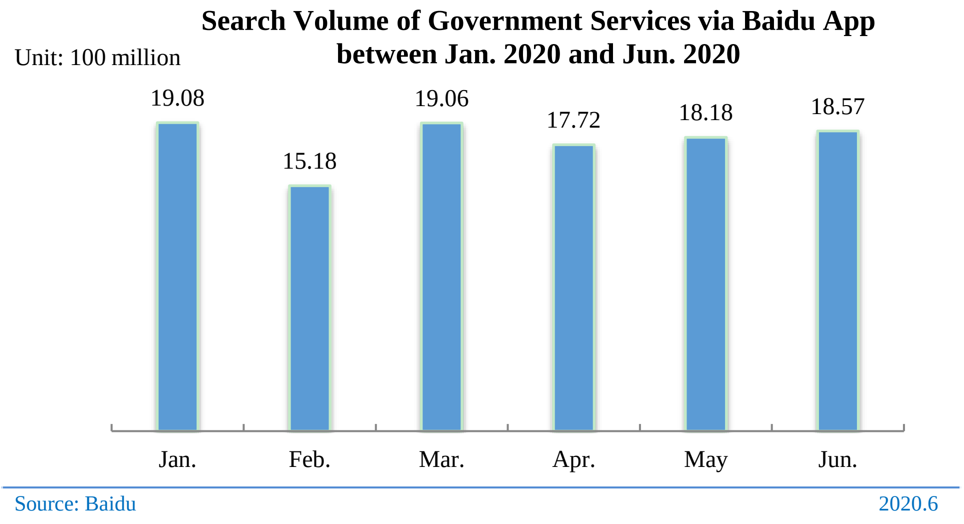

Figure 55 Search Volume of Government Services via Baidu App between Jan. 2020 and Jun.

2020

2. Search of Government Services by Province

In the first half of 2020, Guangdong netizens conducted 1.103 billion searches for government services via Baidu App, ranking first nationwide.

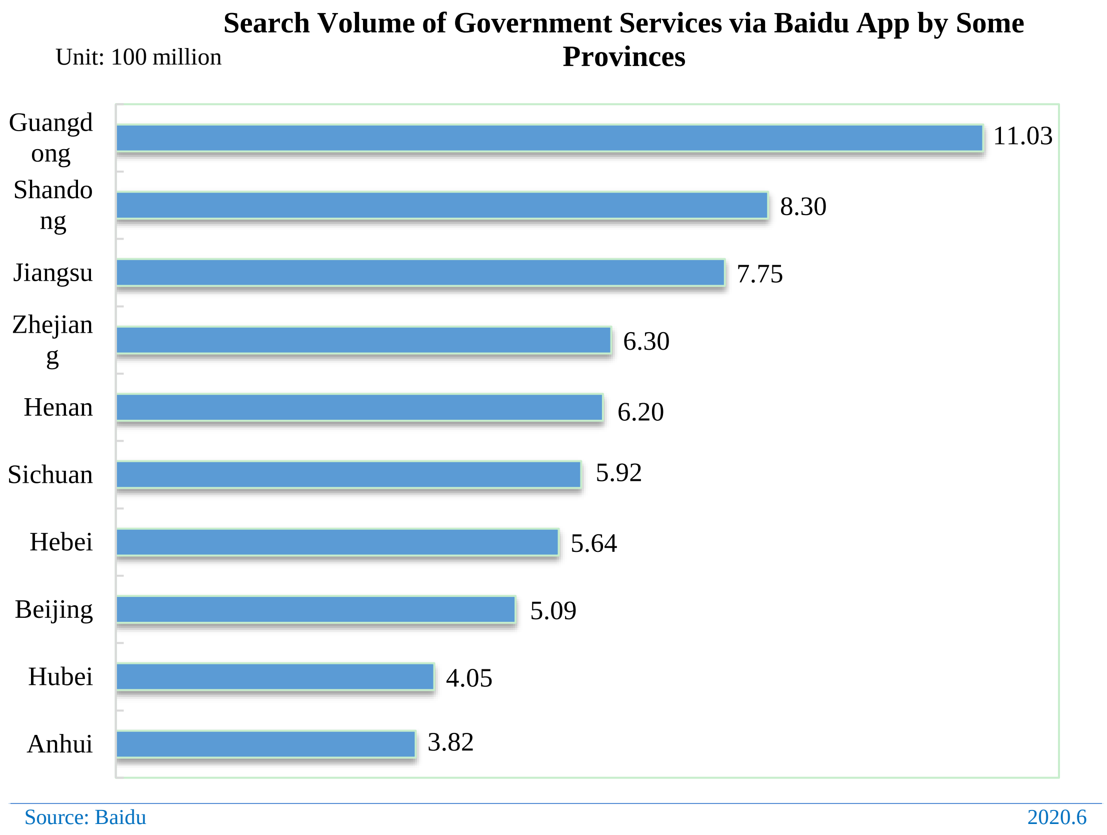

Figure 56 Search Volume of Government Services via Baidu App by Some Provinces

(II) Development of Government Microblogs

1. Overview of Government Microblogs

Up to June 2020, 141,000 government microblogs had been verified by Sina.

Number of Government Microblogs

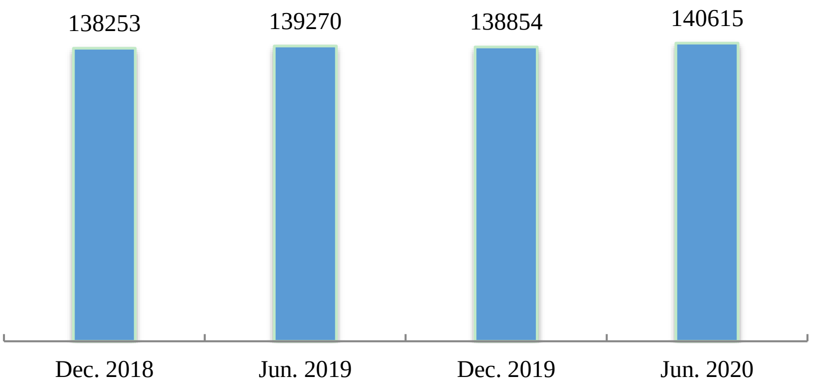

Source: Sina Weibo 2020.6

Figure 57 Number of Government Microblogs

2. The Overview of Government Microblogs by Province

As of June 2020, 31 provinces, autonomous regions and municipalities directly under the Central Government in Mainland China had launched government microblogs. Specifically, Henan province had opened 10,251 government microblogs, ranking first in the country, followed by Guangdong province launching 9,613 government microblogs.

Number of Government Microblogs by Some Provinces

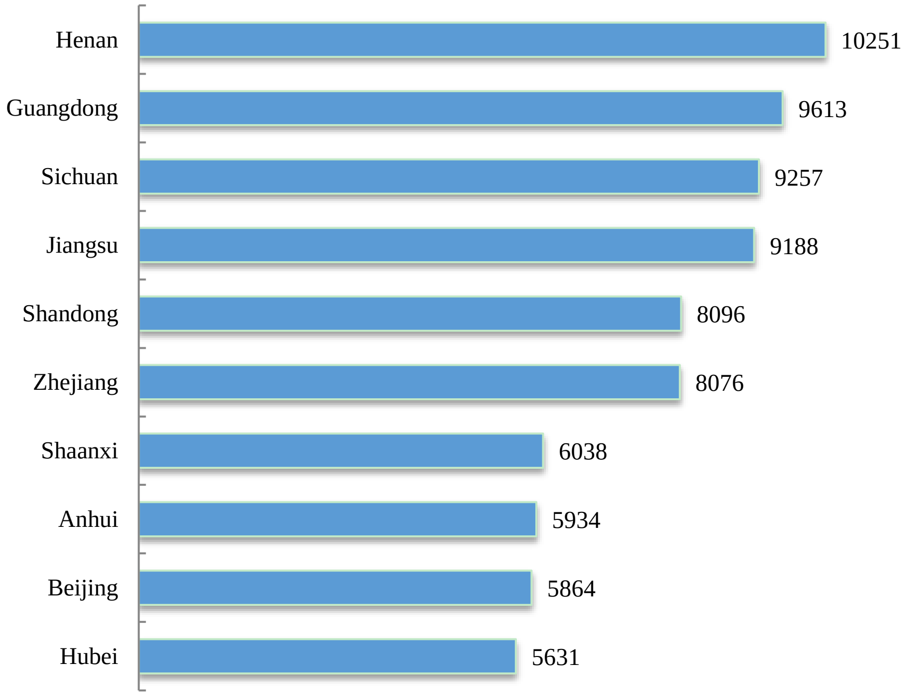

Source: Sina Weibo 2020.6

Figure 58 Number of Government Microblogs by Some Provinces

(III) Development of Zhengwutoutiao and Douyin Accounts

1. Overview of Zhengwutoutiao

As of June 2020, a total of 82,216 Zhengwutoutiao accounts[103] had been opened by governments at all levels.

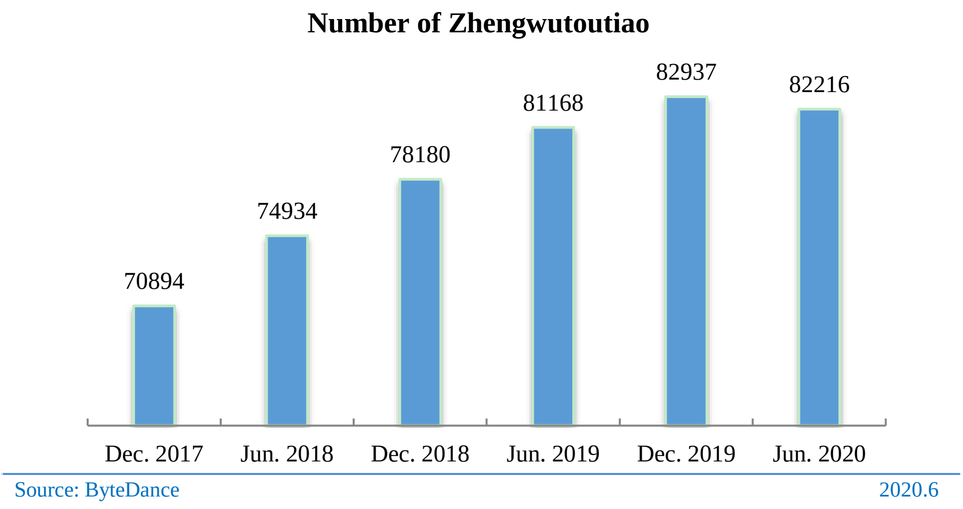

Figure 59 number of Zhengwutoutiao

2. The Overview of Zhengwutoutiao by Province

As of June 2020, 31 provinces, autonomous regions and municipalities directly under the Central Government in Mainland China had launched government microblogs. Among them, Shandong was the province with the largest number of Zhengwutoutiao totaling 8,218. 10 provinces opened more than 3,000 Zhengwutoutiao accounts.

[103] Zhengwutoutiao refers to a public information publishing platform for governmental departments, which is based on the App Top News.

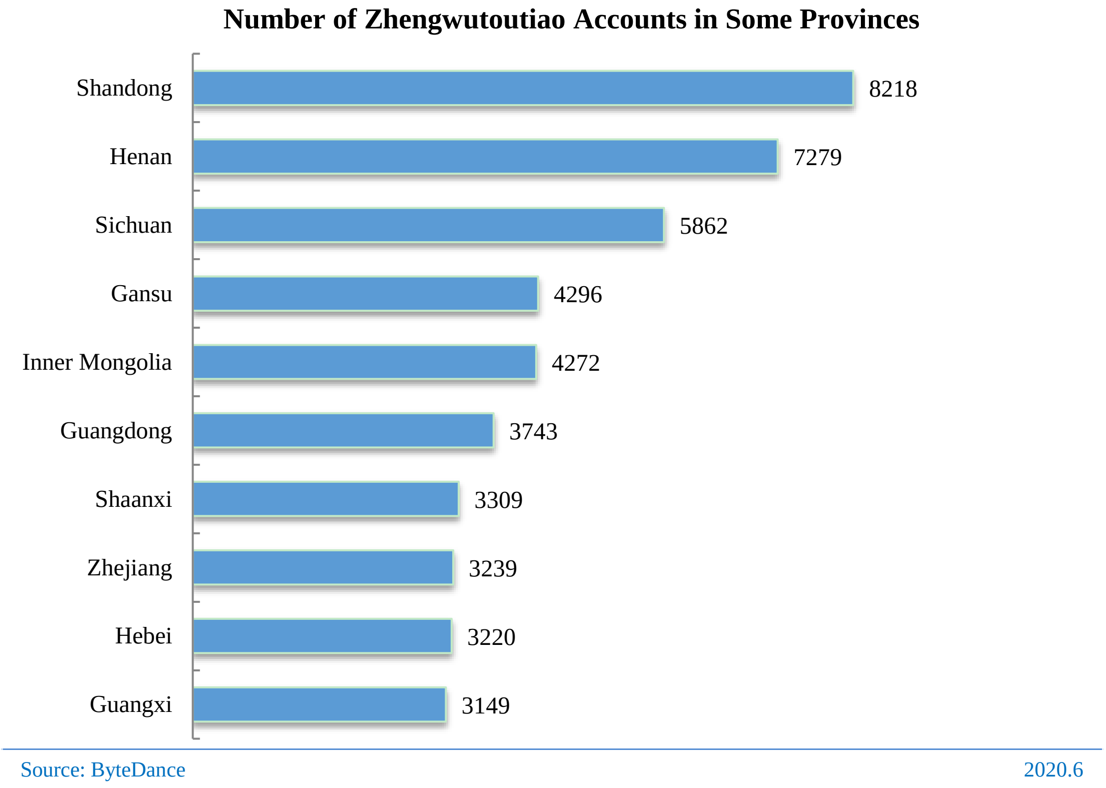

Figure 60 Number of Zhengwutoutiao Accounts in Some Provinces[104]

3. Government Douyin Accounts as a Whole and by Province

Up to June 2020, governments at all levels had opened 25,313 government Douyin accounts. All of China's 31 provinces (autonomous regions and municipalities directly under the central government) have opened their government Douyin accounts. Shandong was the province with the largest number of 1,631 government Douyin accounts.

[104] The data do not include the number of Zhengwutoutiao accounts of ministries and other agencies.

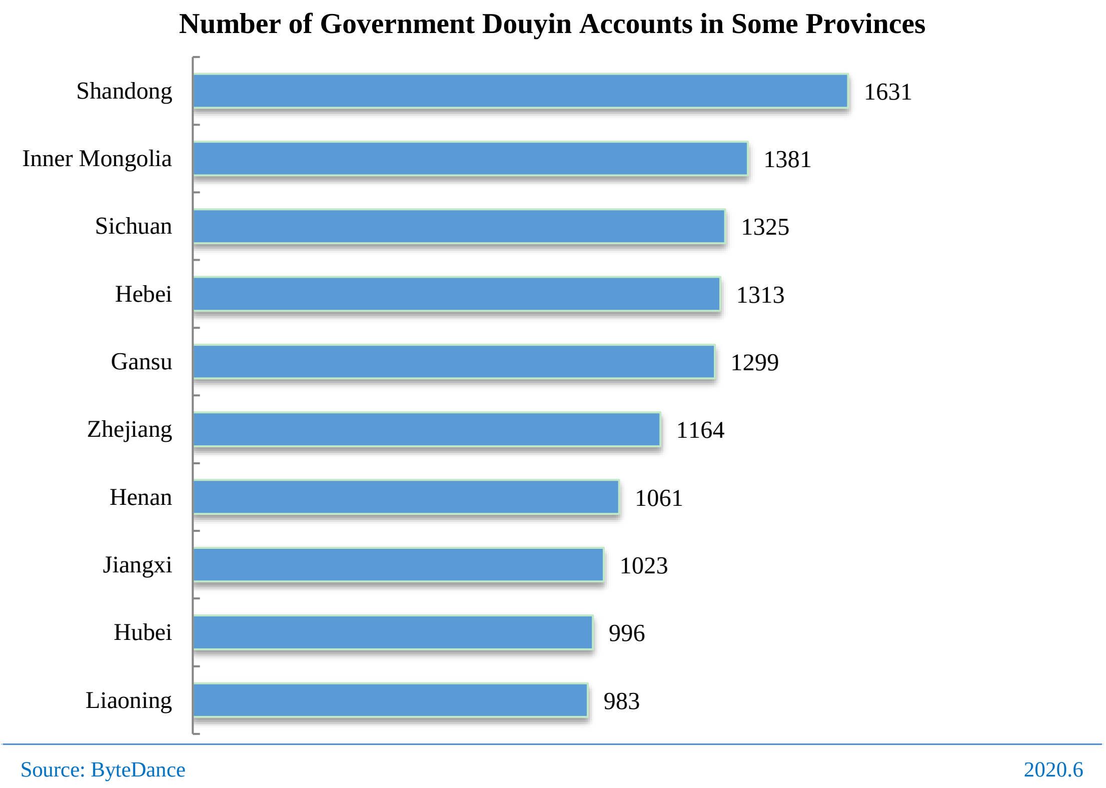

Figure 61 Number of Government Douyin Accounts in Some Provinces[105]

105 The data do not include the number of Douyin accounts for ministries and other agencies.

Chapter Five The Internet Security

I. Cyber Incidents

(I) Proportion of Types of Cybersecurity Problems

The proportion of Chinese Internet users who had not encountered any cybersecurity problems further increased. As of June 2020, 61.6% of Internet users said they had not experienced any cybersecurity problems in the past six months, up 5.2 percentage points from March 2020. The proportion of Internet users experiencing all kinds of cybersecurity problems decreased. Specifically, the proportion of Internet users who had suffered from online fraud decreased by 4.2 percentage points compared with March 2020; that of Internet users who had suffered from personal information leakage also dropped by 2.9 percentage points over March 2020.

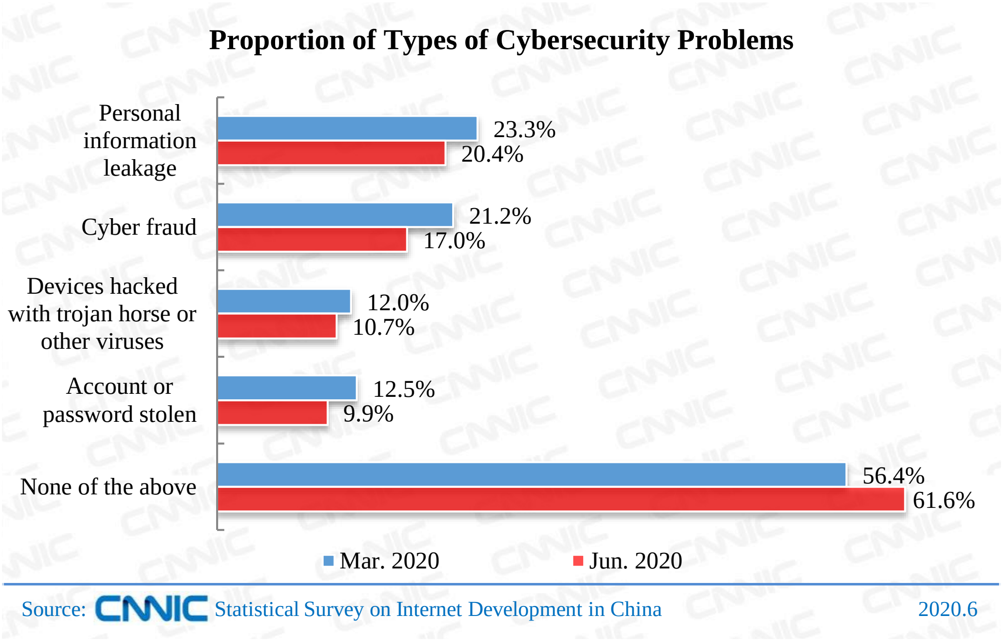

Figure 62 Proportion of Types of Cybersecurity Problems

(II) Proportion of Types of Internet Fraud

Through a further survey of Internet users who had encountered online fraud, it was found that bonus-winning fraud was still the most common type of online fraud, accounting for 49.1%, down 3.5 percentage points from March 2020; fake friends fraud made up 36.0%, down 5.1 percentage points from March 2020; and online shopping fraud took up 31.8%, down 1.2 percentage points from March 2020.

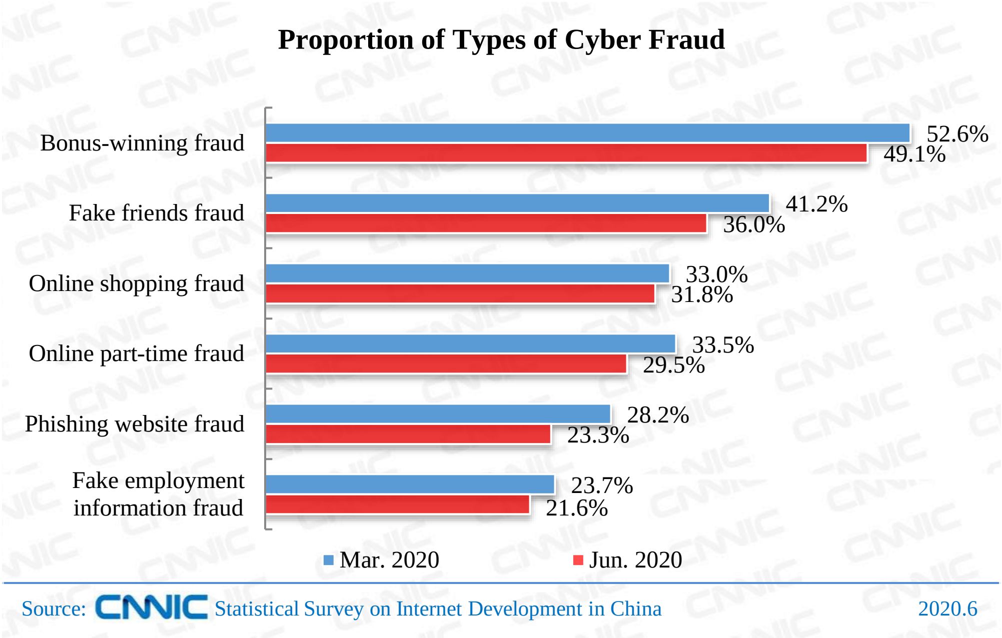

Figure 63 Proportion of Types of Cyber Fraud

II. Website security incidents and information system vulnerabilities

(I) Number of Websites Tampered with by Hackers in China

In the first half of 2020, the National Computer Network Emergency Response Technical Team/Coordination Center of China (CNCERT for short) monitored that the number of tampered[106] websites in China was 147,682[107], a large increase from 50,257 in the same period in 2019[108].

[106] Tampered means that malicious destruction or change of webpage content leads to the fact that a website is unable to work properly or inserted with abnormal webpage content by hackers.
[107] The data is de-duplicated data, the same below.
[108] Since April 2019, CNCERT has expanded the scope of monitoring, so the data have increased greatly.

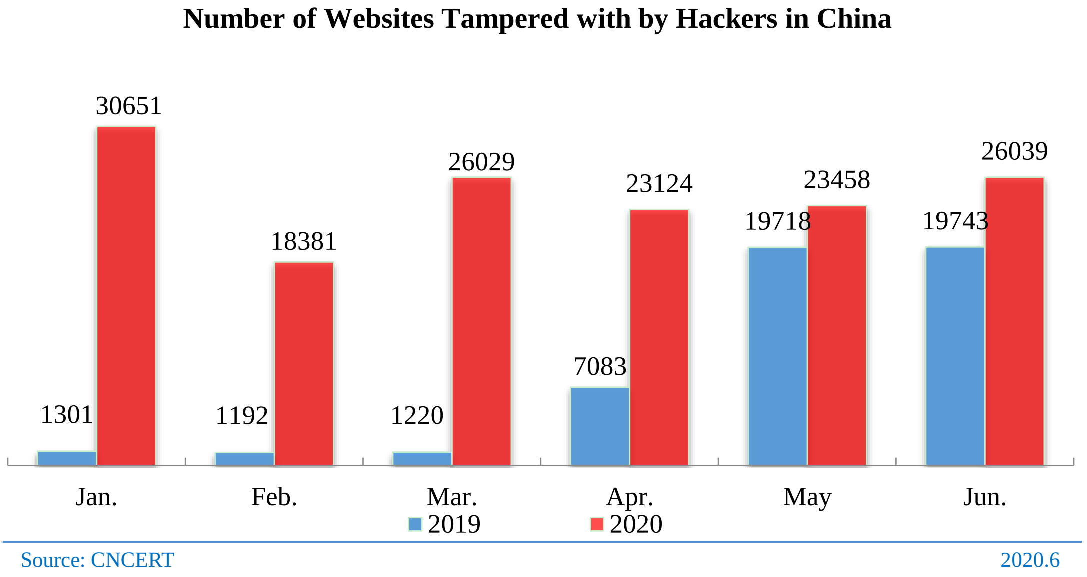

Figure 64 Number of Websites Tampered with by Hackers in China

In the first half of 2020, CNCERT monitored 581 tampered government websites[109] in China, up 57.0%from 370 in the same period of 2019.

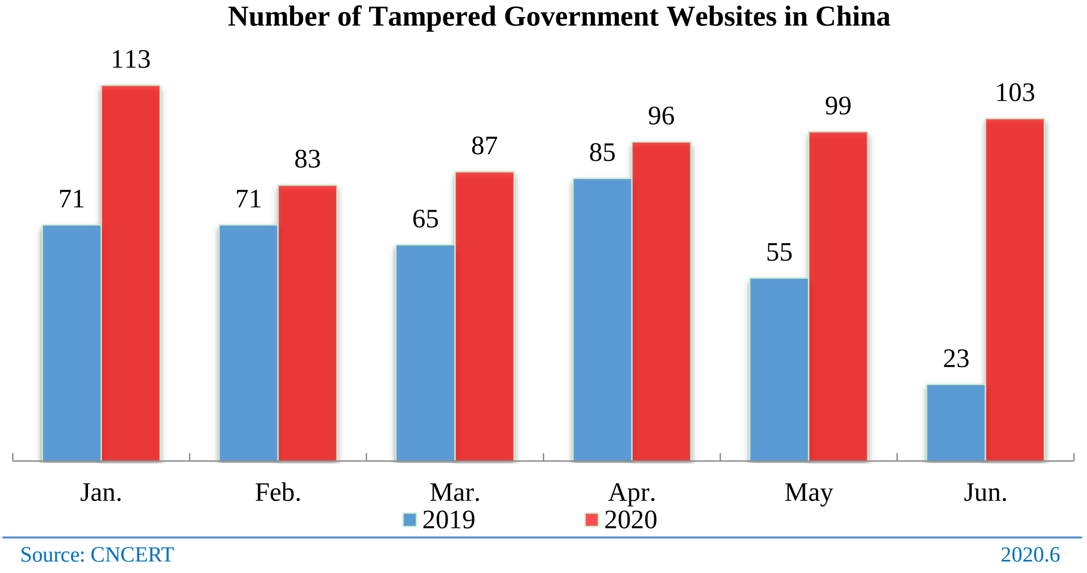

Figure 65 Number of Tampered Government Websites in China

(II) The Number of Websites Implanted with Backdoor Malware in China

In the first half of 2020, CNCERT monitored 40,086 websites implanted with backdoor malwares

[109] Government website refers to a website whose English domain name ends with ".GOV.CN".

in China, up 32.7% from 30,199 in the same period of 2019[110].

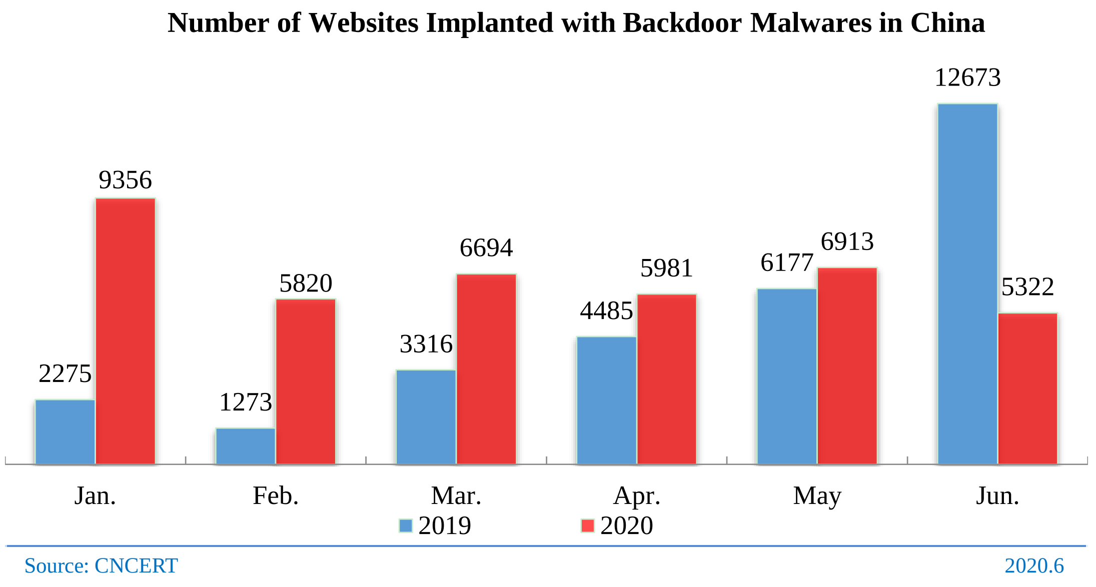

Figure 66 Number of Websites Implanted with Backdoor Malwares in China

In the first half of 2020, CNCERT monitored 127 government websites implanted with backdoor malwares in China, down 69.0% from 410 in the same period in 2019.

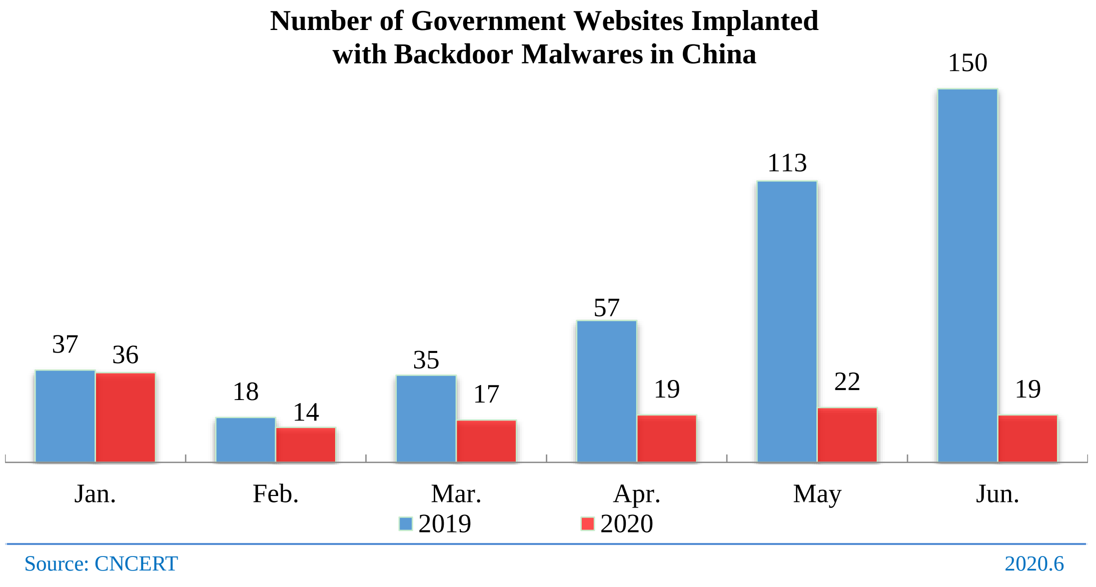

Figure 67 Number of Government Websites Implanted with Backdoor Malwares in China

[110] Since April 2019, CNCERT has expanded the scope of monitoring, so the data have increased greatly.

(III) The Number of Information System Vulnerabilities

In the first half of 2020, China National Vulnerability Database (CNVD)[111] collected 11,073 information system vulnerabilities, up 89.2% from 5,853 in the same period of 2019.

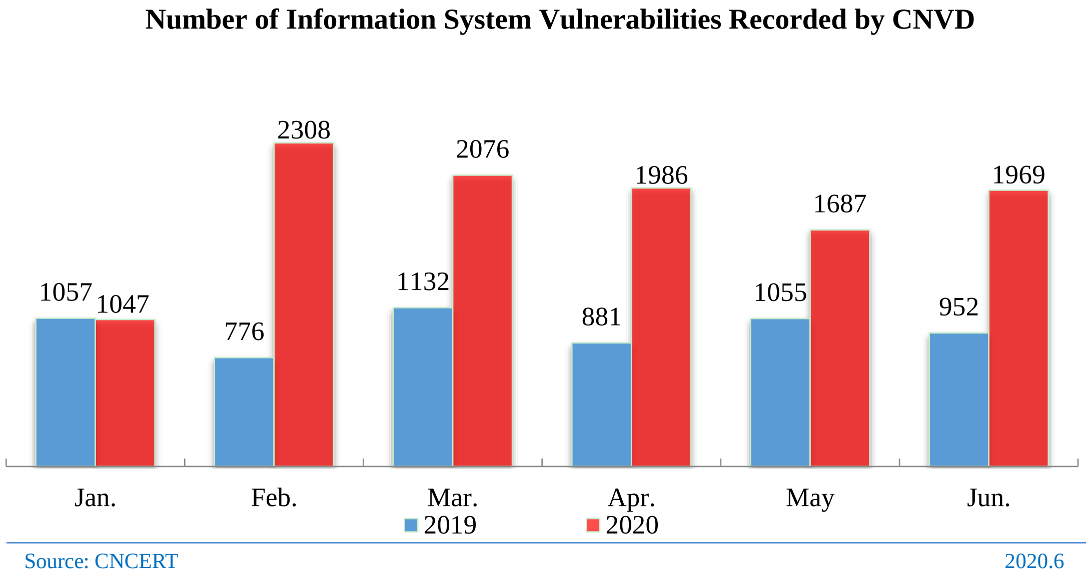

Figure 68 Number of Information System Vulnerabilities Recorded by CNVD

Specifically, 4,280 high-risk vulnerabilities in information systems were collected and recorded, up 128.1% over the same period in 2019 (1,876).

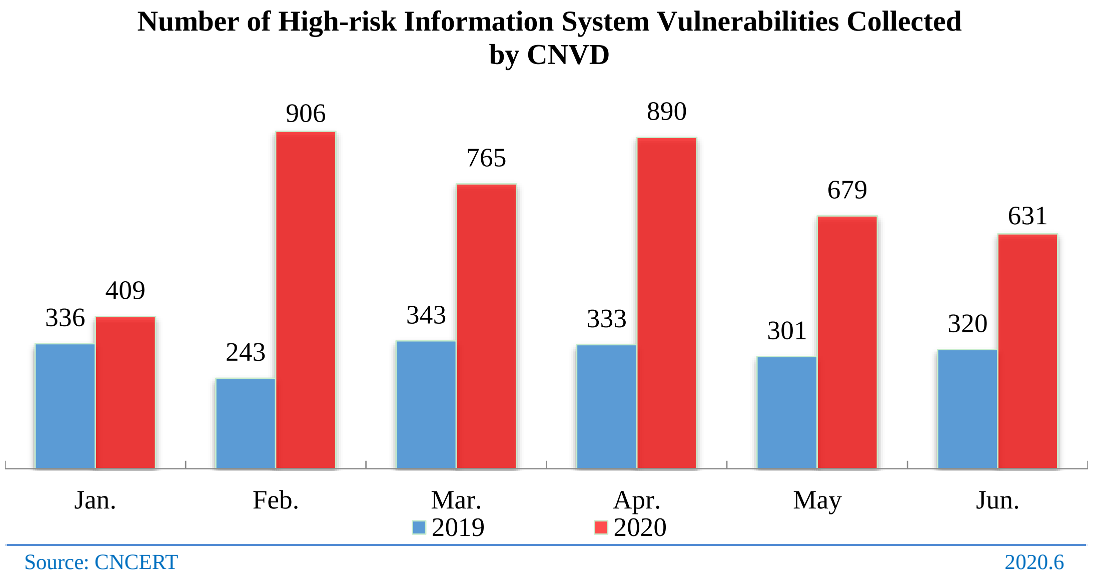

Figure 69 Number of High-risk Information System Vulnerabilities Collected by CNVD

[111] China National Vulnerability Database (CNVD) is a shared knowledge database for information vulnerabilities established by CNCERT in cooperation with China's important information system units, basic telecom carriers, network security vendors, software vendors, and Internet companies.

III. Reporting and Handling of Cybersecurity Incidents

(I) Number of Reported Cybersecurity Incidents Received by CNCERT

In the first half of 2020, CNCERT received 49,468 reports of cybersecurity incidents, up 0.6% from 49,186 reports in the same period of 2019.

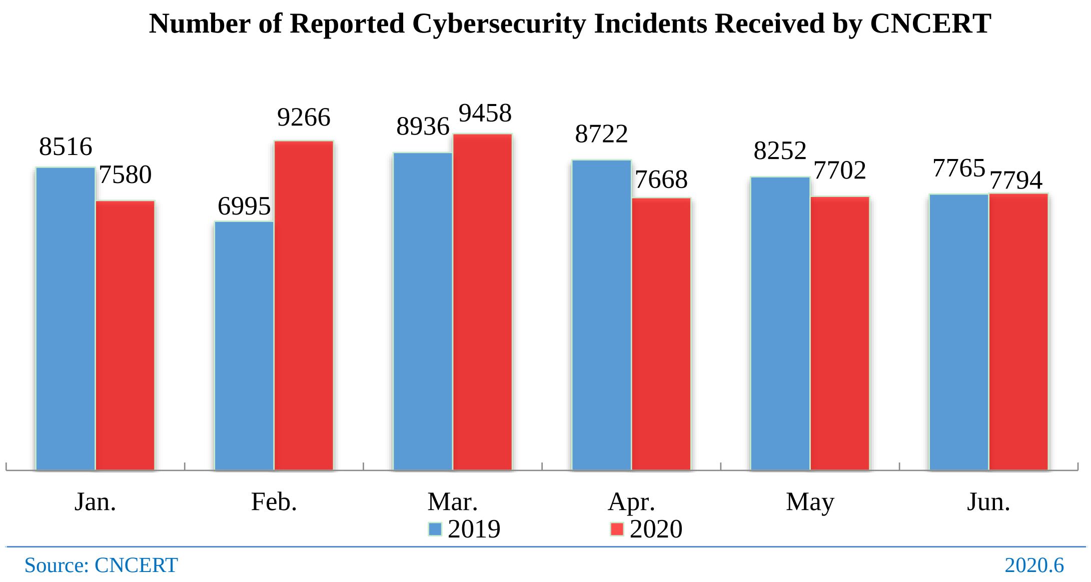

Figure 70 Number of Reported Cybersecurity Incidents Received by CNCERT

(II) Number of Reports Received by China's Network Reporting Departments at all levels

In the first half of 2020, network reporting departments at all levels received 80.88 million reports nationwide, up 17.9% from 68.58 million in the same period of 2019.

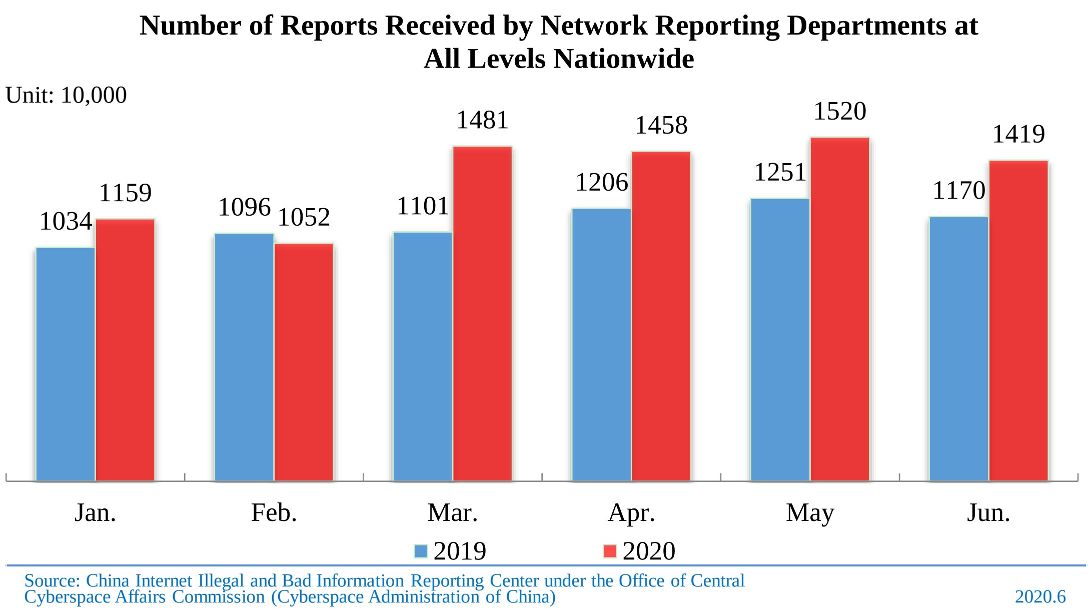

Figure 71 Number of Reports Received by Network Reporting Departments at All Levels Nationwide

Appendix One Survey Methodology

I. Survey Methodology

(I) Survey on Individual Internet Users

1.1 Survey Population

Chinese permanent residents at the age of 6 or above who have residence fixed-line telephones (including home phones and dormitory telephones) or mobile phones

◇ Sample scale

The number of total samples is 30,000, covering 31 provinces (districts and cities).

◇ Division of survey population

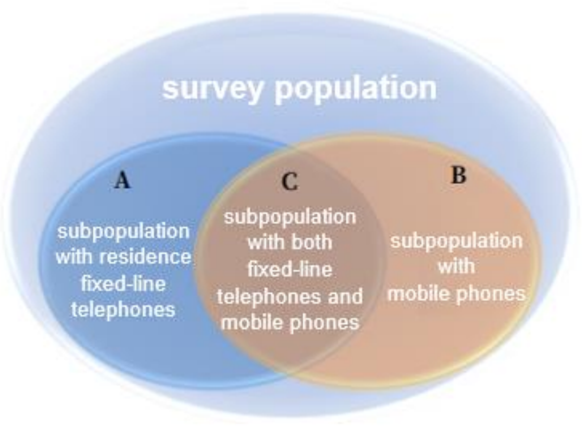

The survey population can be divided into three categories:

Subpopulation A: Survey subpopulation using residence fixed-line telephones (including residents with home phones, students with dormitory telephones, and other users with dormitory telephones);

Subpopulation B: Survey subpopulation with mobile phones;

Subpopulation C: Survey subpopulation with both residence fixed-line telephones and mobile phones (there is an overlap between subpopulation A and subpopulation B, and the overlapped part is subpopulation C), $C=A \cap B$.

1.2 Sampling Method

CNNIC surveys subpopulation A, B and C. Double sampling is adopted for the survey so as to cover as many Internet users as possible. The first sampling frame is subpopulation A, the people with residence fixed-line telephones. The second sampling frame is subpopulation B, the people with mobile phones.

For the survey population with fixed-line telephones, stratified two-stage sampling is adopted. To ensure the sufficient representativeness of samples, the whole country is divided into 31 tiers according to the province, autonomous region and municipality directly under the central government and the sampling is made independently at each tier.

The self-weighted sampling method is adopted for each province. The sample sizes for each district, city and prefecture (including the governed districts and counties) are allocated in accordance with the proportion of the people at the age of 6 or above covered by residence fixed-line telephones in the local area compared to the total covered population in the whole province.

Sampling in subpopulation B is the similar to that in subpopulation A. The whole country is divided into 31 tiers according to the provinces, autonomous regions and municipalities directly under the central government, and sampling is made independently in each tier. Samples are allocated in accordance with the proportion of the residents in each district or city, in order to make the sample allocation in each province conform to the self-weighting method.

To ensure the residence fixed-line telephones are taken with almost the same probability in each district, city or prefecture, that is, the local bureau number with more residence fixed-line telephones will more likely be taken, and to make the phone visit more feasible, the residence fixed-line telephone numbers in each district, city and prefecture are taken according to the following procedures:

For mobile phone user groups, all the mobile bureau numbers in each district, city and prefecture are sampled; a certain quantity of 4-digit random numbers are generated according to the valid sample size in each district, city or prefecture, and then combined with the mobile bureau numbers in each district, city or prefecture to form a number library (local bureau number + the random 4-digit number); randomly order the number library; dial and visit the randomly ordered number library. Survey of the subpopulation with fixed-line telephones is similar to that of the subpopulation with mobile phones: a random number is generated and combined with the local bureau number to form a telephone number, and then such number is dialed and visited. To avoid

repeated sampling, only residence fixed-line telephones are visited.

According to the latest population attribute structure published by the provincial statistical bureaus, we use the method of multi-variable joint weighting to estimate the size of netizens.

1.3 Sampling error

Based on the design, analysis and calculation of sampling, 0.5 percentage points is the estimated maximum allowable absolute error of the proportional target quantity (e.g. the popularity rate of netizens) among the individual netizen survey results, when the confidence is 95%. From this, we can deduce the error range of estimating other kinds of target quantities, such as the scale of netizens.

1.4 Survey Method

The computer-assisted telephone interviewing (CATI) system is adopted for the survey.

1.5 Differences between survey population and targeted population

A study for the subpopulation who are not covered by telephones, conducted by CNNIC at the end of 2005, shows that Internet users are very few in this subpopulation. Currently, the subpopulation is downsizing gradually with the development of our telecom industry. In this survey, there is an assumption, i.e., Internet users who are not covered by fixed-line telephones or mobile phones are negligible.

(Ⅱ) Automatic Online Search and Data Report

Automatic online search is used to conduct technical statistics about the quantity of websites. Statistical data for reporting mainly includes the number of IP addresses.

2.1 Total Number of IP Addresses

The data of IP addresses counted by province come from the IP address databases of Asia-Pacific Network Information Center (APNIC) and CNNIC. Registered data in each database, that can be distinguished by the province which the addresses belong to, can be added respectively by province to generate data of each province. As address allocation is a dynamic process, the statistical data are only for reference. The Ministry of Industry and Information Technology, as the national competent department for IP addresses, also require IP address allocation organizations to report the quantity of IP addresses they own semiannually. To ensure the accuracy

of IP data, CNNIC will compare and verify APNIC statistical data with the reported data to confirm the final quantity of IP addresses.

2.2 Total Number of Websites

It is worked out by CNNIC according to the lists of domain names. The lists of domain names with .CN and .中国 come from the CNNIC database, while the lists of gTLDs come from relevant international domain name registries.

2.3 Total Number of Domain Names

The numbers of domain name under ".CN" and ".中国" come from the database of China Internet Network Information Center (CNNIC).

II. Definitions of Terms in the Report

◇ **Internet Users or Netizens:** Chinese residents at the age of 6 or above who have used the Internet in the past 6 months.

◇ **Mobile Internet Users:** Internet users who have used mobile phones to access and surf the Internet in the past 6 months, but not limited to those surfing the Internet via mobile phones only.

◇ **Computer Internet Users:** Internet users who have used computers to access and surf the Internet in the past 6 months, but not limited to those surfing the Internet via computers only.

◇ **Rural Internet Users:** Internet users who have been living in rural areas of China in the past 6 months.

◇ **Urban Internet Users:** Internet users who have been living in urban areas of China in the past 6 months.

◇ **IP Address:** As the basic resource on the Internet, the IP address functions to identify computers, servers and other devices connected to the Internet. Connection with the Internet can be realized only when an IP address (in any form) is acquired.

◇ **Website:** It refers to a web site with a domain name itself or "www. + domain name". Such domain names include Chinese ccTLD, such as .cn and .中国, and gTLD, and registrants of the domain names are within the territory of P.R.C. For example: for the domain name of "cnnic.cn", it has only one website and the corresponding web address is "cnnic.cn" or "www.cnnic.cn". Other

web addresses with such domain name as the suffix, like “whois.cnnic.cn” and “mail.cnnic.cn”, are regarded as different channels of the website.

◇ **Scope of Survey:** Unless otherwise expressly indicated, data in this Report only refer to mainland China, excluding Hong Kong, Macao and Taiwan.

◇ **Deadline of Survey Data:** The deadline of the statistical survey data is Jun. 30, 2020

Appendix Two Attached Tables of Basic Internet Resources

Table 1 The Number of IPv4 Addresses in Different Regions of China

**Region**	**Number of Addresses**	**Equivalence**
Mainland China	340,527,104	20A+80B+13C
Taiwan	35,700,992	2A+41B+243C
Hong Kong SAR	12,502,272	168B+59C
Macau SAR	336,640	5B+33C

Table 2 The Allocation of IPv4 Addresses among Organizations in Mainland China

**Organization Name**	**Number of Addresses**	**Equivalence**
China Telecom	125,763,328	7A+126B+255C
China Unicom	69,866,752[Note 1]	4A+42B+21C
IP Address Allocation Alliance of CNNIC	61,996,288[Note 2]	3A+177B+253C
China Mobile	35,294,208	2A+26B+140C
China Education and Research Network	16,649,728	254B+14C
China Tietong Telecom	15,796,224[Note 3]	241B+8C
Others	15,160,576	231B+85C
Total	340,527,104	20A+80B+13C

Data sources: Asia-Pacific Network Information Center (APNIC) and China Internet Network Information Center (CNNIC)

Note 1: The addresses of China Unicom include the addresses of former China Unicom and former China Netcom. Specifically, the IPv4 addresses 6316032（96B+96C）of former China Unicom are assigned by CNNIC.

Note 2: As a national Internet registry (NIR) approved by APNIC and national competent authorities in China, CNNIC has organized ISPs, enterprises and public institutions of certain size in China to set up IP Address Allocation Alliance. So far, the total number of IPv4 addresses held by the members of IP Address Allocation Alliance is 84.97 million, equivalent to 5.1A. The IPv4 addresses of the members of IP Address Assignment Alliance listed in the above table do not include those IPv4 addresses already assigned to former China Unicom and Tietong.

Note 3: The IPv4 addresses of China Tietong Telecom are assigned by CNNIC.

Note 4: The deadline for the above statistical data is Jun. 30, 2020.

Table 3 The Number of IPv6 Addresses in Different Regions of China (unit: /32[note1])

Region	Number of Addresses
Mainland China	47,875
Taiwan	2,550
Hong Kong SAR	471
Macau SAR	7

Table 4 The Allocation of IPv6 Addresses among Organizations in Mainland China

Organization Name	Number of IPv6 Addresses
China Telecom	16,387
IP Address Allocation Alliance of CNNIC	14,347 [Note 2]
China Education and Research Network	6,162
China Unicom	4,097
China Mobile	4,097
China Tietong Telecom	2,049 [Note 3]
China Science and Technology Network	17 [Note 4]
Others	719
Total	47,875

Data sources: APNIC and CNNIC

Note 1: /32 as shown in the IPv6 address tables is a method to present IPv6 addresses, and the corresponding number of addresses is $2^{(128-32)} = 2^{96}$.

Note 2: At present, the number of IPv6 addresses held by the members of IP Address Allocation Alliance of CNNIC is 16429/32. The IPv6 addresses held by the members of IP Address Allocation Alliance listed in the above table do not include those IPv6 addresses already assigned to China Tietong Telecom and China Science and Technology Network (CSTNET).

Note 3: The IPv6 addresses of China Tietong Telecom are assigned by CNNIC.

Note 4: The IPv6 addresses of CSTNET are assigned by CNNIC.

Note 5: The deadline for the above statistical data is Jun. 30, 2020.

Table 5 The Proportion of IPv4 Addresses in Each Province/Autonomous Region/Municipality Directly under the Central Government

**Province**	**Proportion**
Beijing	25.49%
Guangdong	9.54%
Zhejiang	6.47%
Shandong	4.89%
Jiangsu	4.76%
Shanghai	4.52%
Liaoning	3.33%
Hebei	2.85%
Sichuan	2.77%
Henan	2.63%
Hubei	2.40%
Hunan	2.36%
Fujian	1.95%
Jiangxi	1.73%
Chongqing	1.68%
Anhui	1.65%
Shaanxi	1.63%
Guangxi	1.38%
Shanxi	1.28%
Jilin	1.21%
Heilongjiang	1.21%
Tianjin	1.05%
Yunnan	0.98%
Inner Mongolia	0.77%
Xinjiang	0.60%
Gansu	0.47%
Hainan	0.47%
Guizhou	0.44%
Ningxia	0.28%
Qinghai	0.18%
Tibet	0.13%
Others	8.92%
Total	100.00%

Data sources: APNIC and CNNIC

Note 1: The above statistics are made on the basis of the location of the IP address owners.

Note 2: The deadline for the above statistical data is Jun 30, 2020.

Table 6 The Numbers of Domain Names, .CN Domain Names and .中国 Domain Names by Province

Province	.CN domain names		.中国 domain names	
	Number	Proportion in .CN domain names	Number	Proportion in .中国 domain names
Guangdong	2292601	9.9%	19924	1.2%
Beijing	2028718	8.8%	28114	1.7%
Jiangsu	1323095	5.7%	10532	0.6%
Sichuan	1252208	5.4%	11458	0.7%
Henan	1234613	5.4%	4469	0.3%
Hubei	1155019	5.0%	4550	0.3%
Shandong	1142068	5.0%	24545	1.4%
Hunan	1044480	4.5%	3029	0.2%
Fujian	945587	4.1%	1505717	88.6%
Jiangxi	850704	3.7%	5238	0.3%
Anhui	793149	3.4%	2882	0.2%
Hebei	789688	3.4%	5698	0.3%
Shanghai	767152	3.3%	8658	0.5%
Shaanxi	647476	2.8%	5755	0.3%
Zhejiang	643307	2.8%	8299	0.5%
Liaoning	630506	2.7%	6703	0.4%
Shanxi	621071	2.7%	1983	0.1%
Guangxi	618614	2.7%	2085	0.1%
Chongqing	526442	2.3%	5546	0.3%
Yunnan	509469	2.2%	5413	0.3%
Guizhou	504230	2.2%	3386	0.2%
Heilongjiang	465123	2.0%	3152	0.2%
Hainan	357137	1.5%	433	0.0%
Jilin	287676	1.2%	1579	0.1%
Gansu	286544	1.2%	1037	0.1%
Tianjin	200361	0.9%	1623	0.1%
Inner Mongolia	182955	0.8%	1263	0.1%
Xinjiang	64492	0.3%	965	0.1%
Ningxia	45193	0.2%	412	0.0%
Qinghai	16345	0.1%	184	0.0%
Tibet	14440	0.1%	439	0.0%
Others	803913	3.5%	14355	0.8%
Total	23044376	100.0%	1699426	100.0%

Data sources: APNIC and CNNIC

Note: The deadline for the above statistical data is Jun 30, 2020.

Appendix Three Supporting Organizations

We would like to express our heartfelt thanks to the following organizations that have supported the collection of data in this report. (Not listed in any particular order)

Ministry of Industry and Information Technology	National Bureau of Statistics
E-government Research Center, Party School of the Central Committee of CPC (National Academy of Governance)	National Computer Network Emergency Response Technical Team / Coordination Center of China（CNCERT）
Reporting Center for Illegal and Inappropriate Internet Information, Office of the Central Cyberspace Affairs Commission (Cyberspace Administration of China) (12377)	Computer Network Information Center, Chinese Academy of Sciences
China Telecom E-cloud Company	Beijing Ucap Information Techonology Co., Ltd.
Baidu Online Network Technology (Beijing) Co., Ltd.	Beijing Micro Dream Network Technology Co., Ltd. (Micro-blog)
Beijing Bytedance Technology Co., Ltd.（Toutiao）	

We also extend our sincere thanks to other organizations that have helped us in the course of compiling and revising the Report.

The copyright of this report is reserved by China Internet Network Information Center (CNNIC).

Please indicate the source in case of citation or reproduction.
